# Molecular targets and the emerging role of copper radionuclides in prostate cancer theranostics

**DOI:** 10.1186/s13550-025-01325-4

**Published:** 2025-11-18

**Authors:** Diana Rodrigues, Alexandra I. Fonseca, João N. Moreira, Célia Gomes, Antero Abrunhosa

**Affiliations:** 1 Coimbra Institute for Biomedical Engineering and Translational Research, Institute for Nuclear Sciences Applied to Health (CIBIT/ICNAS), University of Coimbra, Coimbra, 3000-548 Portugal; 2 CNC-UC — Center for Neuroscience and Cell Biology, Centre for Innovative Biomedicine and Biotechnology Consortium (CIBB), Faculty of Medicine (Pole 1), University of Coimbra, Coimbra, 3004-504 Portugal; 3 Univ Coimbra — CIBB, Faculty of Pharmacy, University of Coimbra, Coimbra, 3000-548 Portugal; 4 CIBB, University of Coimbra, Coimbra, 3000-548 Portugal; 5 Faculty of Medicine, Coimbra Institute for Clinical and Biomedical Research (iCBR), University of Coimbra, Coimbra, 3000-548 Portugal; 6 Clinical Academic Center of Coimbra (CACC), Coimbra, 3000-075 Portugal

**Keywords:** Prostate cancer, Copper radionuclides, Radiopharmaceuticals, Positron emission tomography, Targeted radionuclide therapy, Theranostics

## Abstract

**Background:**

Prostate cancer (PCa) remains one of the most commonly diagnosed malignancies and a leading cause of cancer-related morbidity and mortality among men worldwide. Despite considerable progress in diagnostic and therapeutic modalities, conventional approaches often fall short in capturing disease heterogeneity and managing advanced or treatment-resistant cases. Over the past years, molecular imaging and targeted radionuclide therapy within a theranostic framework have emerged as powerful tools to potentially overcome these limitations. In this context, copper radioisotopes—particularly copper-61 (T_1/2_ = 3.33 h; 61% β^+^), copper-64 (T_1/2_ = 12.7 h; 17% β^+^, 39% β^–^), and copper-67 (T_1/2_ = 2.58 d; 100% β^–^)—have garnered considerable attention due to their favorable half-lives, straightforward coordination chemistry, and optimal physical decay properties for both imaging and therapy.

**Main body:**

This review comprehensively examines the progress and prospects of copper-based radiopharmaceuticals for PCa theranostics, with particular emphasis on agents targeting prostate-specific membrane antigen (PSMA) and gastrin-releasing peptide receptor (GRPR), the two most extensively studied and clinically relevant molecular targets in this setting. Alternative markers are also discussed as promising avenues to tackle disease heterogeneity and expand the clinical applicability of these conjugates. By consolidating preclinical and clinical evidence, we aim to identify current challenges and opportunities in the development of copper-based radiopharmaceuticals, and contribute to a paradigm shift toward the widespread clinical adoption of these novel radiopharmaceutical platforms for improved patient care.

**Conclusion:**

Copper-based radiopharmaceuticals represent a promising class of agents with the potential to refine PCa management. As research advances, these compounds are poised to enhance diagnostic precision and therapeutic efficacy, paving the way for more personalized strategies and favorable clinical outcomes.

## Background

Prostate cancer (PCa) remains a major global health concern, ranking among the most frequently diagnosed malignancies and contributing significantly to cancer-related mortality in men [1]. Despite ongoing efforts to advance diagnostic and therapeutic strategies, the clinical management of PCa continues to pose critical challenges. Traditional diagnostic tools suffer from limited sensitivity and specificity, frequently leading to overdiagnosis, underestimation of tumor aggressiveness, or missed detection of high-risk disease [2–4]. Standard imaging modalities likewise offer suboptimal performance in staging and monitoring, especially in cases of biochemical recurrence or metastatic involvement [5, 6]. In addition, conventional therapeutic approaches often fall short in controlling disease progression, particularly in refractory or advanced stages [7, 8].

To overcome these limitations, molecular imaging and targeted radionuclide therapy (TRT) have recently emerged as transformative tools in the era of precision oncology. Prostate-specific membrane antigen (PSMA)-targeted radiopharmaceuticals have revolutionized the paradigm of PCa care by enabling highly specific imaging and selective delivery of cytotoxic radiation. Clinically validated agents such as [^68^Ga]Ga-PSMA-11 [9] for PET imaging and [^177^Lu]Lu-PSMA-617 [10, 11] or [^225^Ac]Ac-PSMA-617 [12] for TRT have demonstrated substantial improvements in diagnostic accuracy and therapeutic efficacy in patients with metastatic castration-resistant disease, with minimal toxicity.

The unique physical and chemical properties of copper radionuclides such as copper-61 (T_1/2_ = 3.33 h; 61% β^+^), copper-64 (T_1/2_ = 12.7 h; 17% β^+^, 39% β^–^), and copper-67 (T_1/2_ = 2.58 d; 100% β^–^) [13] have positioned them as promising agents for both diagnostic imaging and therapeutic applications in nuclear medicine, most notably within a theranostic framework. This review provides a comprehensive overview of preclinical and clinical studies investigating copper-labeled radiopharmaceuticals for PCa imaging and therapy. It also outlines the most relevant copper radioisotopes in this context, highlights key molecular targets, and acknowledges recent translational advances and their prospective role in enhancing clinical decision-making.

## Main text

## Relevant copper radionuclides for nuclear medicine applications

Copper radionuclides have attracted significant interest in nuclear medicine due to their versatile chemical properties, broad range of half-lives, and emission profiles well-suited for both diagnostic imaging and therapeutic applications.

The production of copper radionuclides typically relies on the use of nickel, zinc, or cobalt as target materials at nuclear facilities equipped with a cyclotron, reactor, or generator. Multiple production methods have been proposed and compared over the years, each offering distinct advantages and limitations [14, 15]. The selected production route and stipulated irradiation parameters—such as the energy of the incident particle beam, electrical current, and irradiation time—significantly influence the key characteristics of the resulting irradiated target. The isotopic enrichment of the target material and the presence of trace contaminants are also critical aspects to evaluate. These factors have a direct impact on subsequent radiolabeling yields and are ultimately determinative of the overall quality of the radiopharmaceutical. Therefore, several interrelated considerations must be addressed when producing copper radionuclides for routine clinical use. A primary concern is the practical feasibility and consistent availability of the required particle beam and irradiation infrastructure, whether based on accelerator or reactor technology. Equally important are the overall yield of the process, the radionuclidic purity of the produced radioisotope, and its specific activity—with particular emphasis on minimizing the presence of stable copper isotopes that could compromise therapeutic or diagnostic effectiveness of the final product. Additionally, the potential for scaling up production to support broader clinical demand or widespread commercial distribution is also an essential consideration when selecting the most appropriate nuclear pathway. After radionuclide production, the irradiated target material is post-processed and purified to remove “cold” (i.e., non-radioactive) elements and radionuclidic impurities co-produced during irradiation, ensuring that the radionuclide precursor complies with the required quality standards for human use. The detailed description of the different methodologies for post-processing of copper radionuclides falls beyond the scope of this review, but has been recently published by Fonseca et al. [16].

The most relevant copper radionuclides include copper-60, copper-61, copper-62, copper-64, and copper-67. Among these, copper-61, copper-64, and copper-67 stand out as particularly promising for theranostic use owing to their optimal half-lives and favorable decay properties. Table 1 provides a comparative overview of this triad, including key physical decay characteristics and the most common production routes.

### Copper-61

**Fig. 1:**
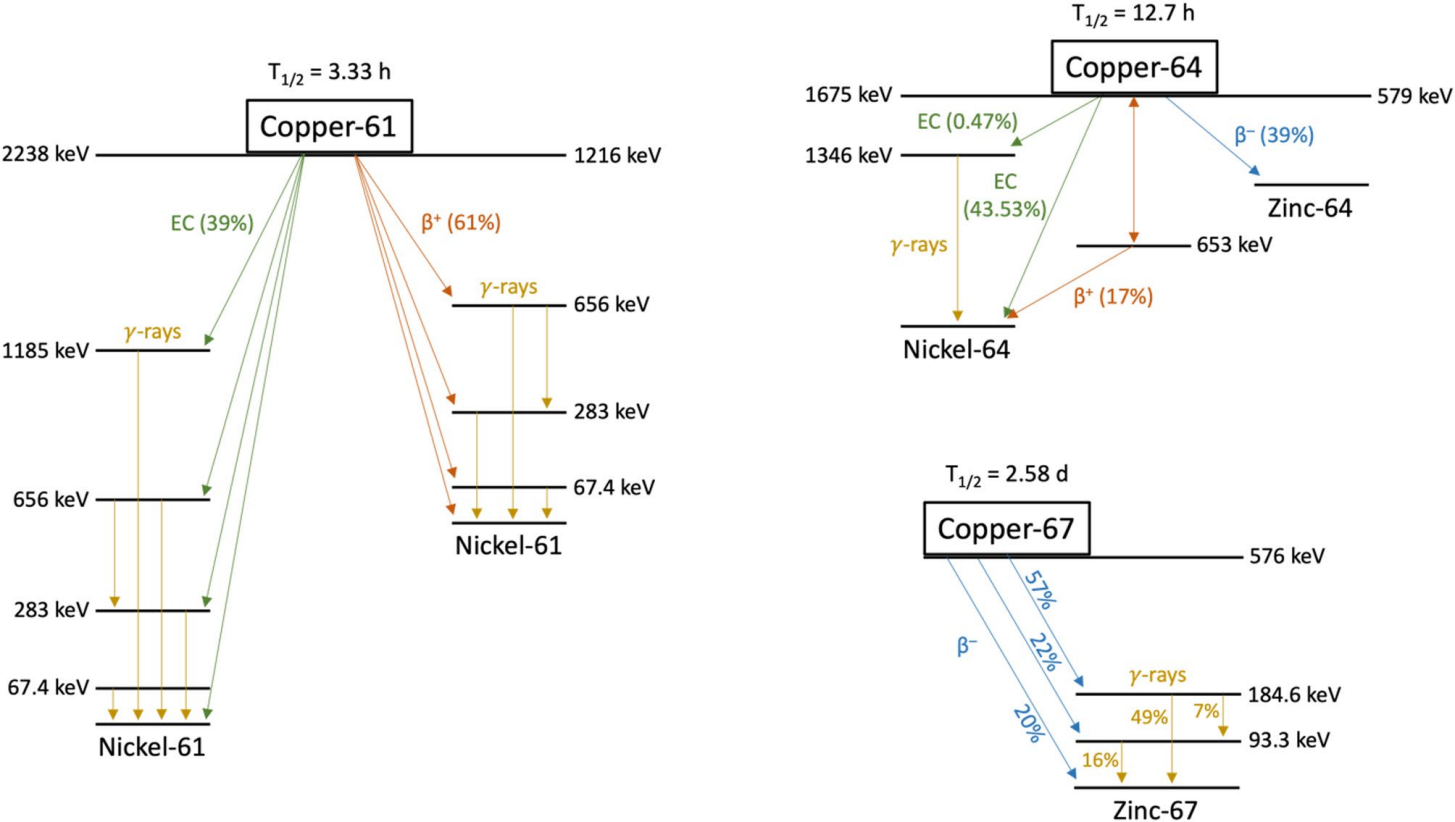
Decay schemes of copper-61, copper-64, and copper-67

Copper-61 is an emerging β^+^-emitter that has recently been gathering pace in the field of nuclear medicine due to its usefulness for PET imaging and favorable physical decay properties compared to the gold standard gallium-68, or its isotopic analogue copper-64. Copper-61 decays by β^+^ emission (61%; E_max β_+ = 1216 keV) and electron capture (39%) with a half-life of 3.33 h (Fig. 1), allowing for high-quality imaging with reduced radiation exposure to the patient, while still providing a sufficient time window for radiopharmaceutical synthesis and distribution, and biological targeting. Several cyclotron-based production routes have been proposed over the years for copper-61 production. Foremost among them is the proton irradiation of highly enriched nickel-61, or zinc-64—either natural or enriched, liquid or solid—targets. The ^61^Ni(p,n)^61^Cu nuclear reaction is a well-established method, mirroring the production process of copper-64, with the primary difference of using nickel-61 instead of nickel-64 as target material [17, 18]. Although this route grants higher production yields, it is hindered by the extremely high cost of enriched nickel targets. On the other hand, the ^64^Zn(p,α)^61^Cu nuclear reaction is a more cost-effective alternative—especially if natural or liquid, instead of enriched or solid targets are used—while also providing relatively high yields and radionuclidic purities, depending on the choice of the target material [19–22]. Copper-61 can also be produced by irradiating enriched nickel-60 or natural nickel targets with deuteron beams [17, 23], or natural nickel [24] and cobalt [25] targets with alpha particles, although the limited availability of deuteron and alpha beams makes these production routes less accessible and may contribute to higher production costs.

### Copper-64

Copper-64 is the most widely used copper isotope in nuclear medicine, mainly due to its unique decay characteristics, which make it a dual-purpose (theranostic) radionuclide suitable for both PET imaging and TRT, and well-established production routes. Copper-64 decays by β^+^ (17%; E_max β_+ = 653 keV) and β^–^ (39%; E_max β_– = 579 keV) emission, and electron capture (44%) (Fig. 1), while also concomitantly emitting Auger electrons, which contribute to its therapeutic potential. Its half-life of 12.7 h aligns well with the biological half-life of heavier targeting molecules, such as monoclonal antibodies (mAbs) or larger antibody fragments, which exhibit slower pharmacokinetics. This prolonged physical half-life not only allows for extended imaging protocols but also facilitates centralized radiopharmaceutical production and distribution across broader geographic areas. Despite its relatively low branching ratio, the β^+^ particles emitted by copper-64 are of low energy and short range, enabling high spatial resolution PET imaging. Additionally, the minimal associated γ-emissions reduce background noise, thereby enhancing overall image quality. The most widely adopted and clinically relevant method for copper-64 production is the cyclotron-based ^64^Ni(p,n)^64^Cu nuclear reaction, which offers high production yields with high specific activity and excellent radionuclidic purity, whether solid or liquid targets are used [26, 27]. However, enriched nickel-64 is tremendously expensive and must be efficiently recovered and recycled to make the process economically sustainable. Given the high cost of nickel-64, alternative cyclotron-based production routes involving the irradiation of zinc targets with proton or deuteron beams have also been documented [28–31]. These approaches aim to provide more cost-effective solutions, although they typically result in lower radionuclidic purity and often introduce additional byproducts that complicate radiochemical processing. Copper-64 can also be produced in nuclear reactors via the ^64^Zn(n,p)^64^Cu or ^63^Cu(n,γ)^64^Cu nuclear reaction by fast neutron capture on natural or enriched zinc targets [32–34], or thermal neutron capture on natural or enriched copper targets [33, 35], respectively. These methods are reportedly inexpensive and technically simple but either yield low specific activities or likewise result in the co-production of contaminants and radionuclidic impurities that further compound purification procedures.

In 2020, [^64^Cu]Cu-DOTATATE (Detectnet™) received U.S. Food and Drug Administration (FDA) approval for PET imaging of somatostatin receptor-positive neuroendocrine tumors [36]. This regulatory achievement marks a significant milestone in the field of nuclear medicine and demonstrates that copper-based radiopharmaceuticals are no longer a theoretical prospect but a practical reality, which has intensified interest in copper radionuclides and offered a strong foundation for continued research and innovation in order to expand their clinical applicability.

### Copper-67

Copper-67 is the longest-lived (T_1/2_ = 2.58 d) copper radioisotope and the most challenging to produce, which explains its limited availability and the reduced number of studies conducted to date. However, its favorable decay properties—closely comparable to those of the clinically established lutetium-177—as a promising therapeutic radionuclide along with the significant advantage of potentially being paired with its shorter-lived, positron-emitting isotopic counterparts for theranostic applications are expected to prompt an increase in demand, ultimately encouraging further efforts to make it readily accessible through commercial supply chains in the near future. Copper-67 decays exclusively by β^–^ emission (100%; E_max β_– = 576 keV), with concomitant emission of γ-rays at energies appropriate for SPECT imaging (Fig. 1). The most common method for producing copper-67 involves proton irradiation of enriched zinc-68 via the ^68^Zn(p,2p)^67^Cu reaction [37–39], which results in favorable yields and high specific activity but requires highly energetic proton beams, posing significant technical challenges. Another cyclotron-based production route relies on proton or deuteron bombardment of enriched zinc-70 targets through nuclear reactions such as ^70^Zn(p,α)^67^Cu [40] or ^70^Zn(d,αn)^67^Cu [41]. These reactions provide moderate yields with relatively lower energy requirements compared to the previously mentioned (p,2p) method. However, the need for highly enriched zinc-70 significantly raises production costs, making target recovery and reuse essential. Although the production of copper-67 requires high-energy cyclotrons, which restricts its use in research and standard clinical settings, its relatively long half-life offers the potential for centralized production and distribution from dedicated facilities equipped with the necessary infrastructure. Neutron-induced reactions have also been explored for copper-67 production. In nuclear reactors, it can be produced using fast fission neutrons via the ^67^Zn(n,p)^67^Cu reaction [42–44]. This approach requires highly enriched target material to minimize competing side reactions, as well as high neutron fluxes to achieve satisfactory yields. Alternatively, copper-67 can be obtained in accelerator-driven neutron generators (ADNG), where fast neutrons are produced by proton or deuteron bombardment of light-element targets such as carbon, lithium, or beryllium. The higher neutron energies available in these systems enable production through the ^68^Zn(n,np)^67^Cu and ^68^Zn(n,d)^67^Cu reactions, and also enhance the yields from the ^67^Zn(n,p)^67^Cu pathway [45–50]. Another promising strategy that has been proposed is the photonuclear production of copper-67 via the ^68^Zn(γ,p)^67^Cu reaction [51–54], which utilizes high-energy bremsstrahlung photons obtained from electron linear accelerators (LINACs). When isotopically enriched targets are used, this method can produce large amounts of copper-67 with high radionuclidic purity and specific activity. These advantages, combined with the widespread availability of LINACs, make this approach particularly attractive for scalable production.

**Table 1:** Physical and nuclear properties of copper radioisotopes most relevant for medical applications (i.e., copper-61/64/67), including their half-lives, decay modes, characteristic particle energies, primary clinical uses (imaging or therapy), and common production methods and corresponding nuclear reactions

Radionuclide	Half-life	Decay mode	Maximum β^+^ energy (keV)	Maximum β^–^ energy (keV)	Main γ energies (keV)	Application	Production route	Nuclear reactions
Copper-61	3.33 h	β^+^ (61%) EC (39%)	1216 (51%)	–	283 (12%) 511 (121%) 656 (11%)	PET imaging	Cyclotron	^61^Ni(p,n) ^64^Zn(p,α) ^nat^Zn(p,x) ^60^Ni(d,n) ^nat^Ni(d,x) ^nat^Ni(α,x) ^nat^Co(α,2n)
Copper-64	12.7 h	β^+^ (17%) β^–^ (39%) EC (44%)	653 (17%)	579 (39%)	511 (35%) 1346 (0.47%)	PET imaging & Therapy	Cyclotron	^64^Ni(p,n) ^66^Zn(p,2pn) ^67^Zn(p,α) ^nat^Zn(d,x) ^64^Zn(d,2p)
							Reactor	^64^Zn(n,p) ^63^Cu(n,γ)
Copper-67	2.58 d	β^–^ (100%)	–	576 (20%)	91 (7%) 93 (16%) 185 (49%)	SPECT imaging & Therapy	High-energy cyclotron	^68^Zn(p,2p) ^70^Zn(p,α) ^70^Zn(d,αn)
							Reactor/ADNG	^67^Zn(n,p) ^68^Zn(n,np) ^68^Zn(n,d)
							Photonuclear reaction	^68^Zn(γ,p)

## Copper coordination chemistry

Copper is a transition metal with versatile redox chemistry and essential biological roles. It exists predominantly in two oxidation states: Cu(I) (cuprous) and Cu(II) (cupric) [55]. For radiopharmaceutical synthesis, copper radioisotopes are preferred in Cu(II) oxidation state due to its greater kinetic stability, well-characterized coordination chemistry, and compatibility with aqueous radiolabeling conditions. While chelator-free use of the radiometal (i.e., as copper chloride) is possible in niche contexts, most applications involve the radiolabeling of biomolecules that lack intrinsic metal-binding sites, making it difficult to achieve stable and specific coordination without an external ligand. Therefore, chelating agents act as engineered binding pockets that tightly and selectively coordinate metal ions, allowing the radiometal to be stably attached to the targeting vector while preserving target specificity and favorable pharmacokinetics. An ideal chelator for copper-based radiopharmaceuticals should exhibit high thermodynamic stability and kinetic inertness to prevent in vivo transchelation [56], enable rapid and efficient radiolabeling under mild conditions, be compatible with a diverse array of biological vectors, and ensure minimal off-target interactions with desirable clearance profiles.

Copper(II) typically prefers square planar, distorted square planar, square pyramidal, trigonal bipyramidal, or distorted octahedral geometries [57, 58]. Chelators that enforce these geometries and provide strong donor atoms—primarily nitrogen, oxygen, and sulfur [58, 59]—tend to form highly stable complexes. Macrocyclic ligands, especially those with rigid frameworks or cross-bridged structures, are particularly effective at locking copper into kinetically inert configurations.

**Fig. 2:**
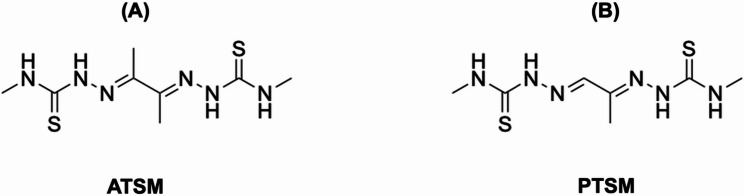
Schematic structure of the most commonly used bis(thiosemicarbazone) ligands with copper radionuclides. (**A**) ATSM, and (**B**) PTSM

The evolution of copper chelators reflects advances in coordination chemistry and radiopharmaceutical design, with each successive generation of ligands developed to address specific limitations of its predecessors. Bis(thiosemicarbazones) such as ATSM and PTSM (Fig. 2) were among the earliest copper chelators used in nuclear medicine, predominantly for imaging blood perfusion and hypoxia [60–63]. Their ability to undergo redox cycling between Cu(II) and Cu(I) allows intracellular trapping of copper in the target tissues under reducing conditions [64]. However, these ligands form relatively labile complexes, and their low kinetic stability leads to transchelation and off-target accumulation [65]. Moreover, their non-specific biodistribution and limited control over copper retention restrict their use in targeted imaging or therapy.

**Fig. 3:**
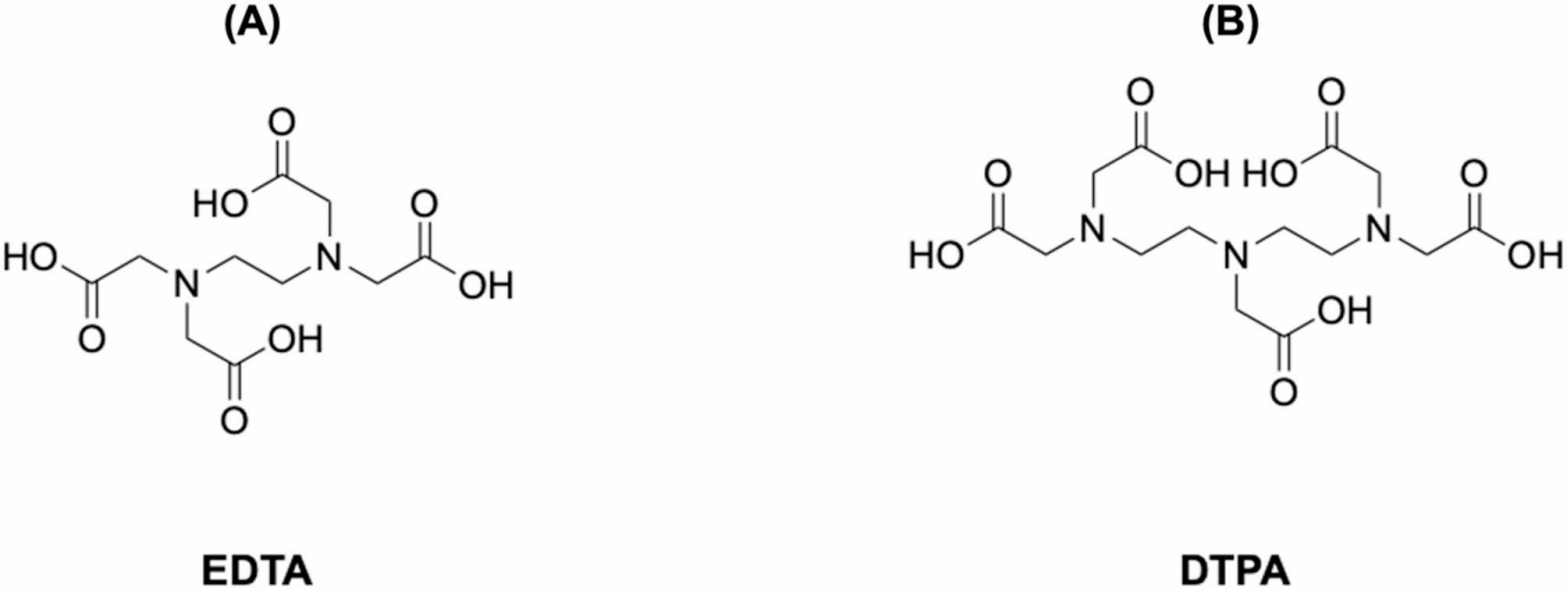
Schematic structure of classic acyclic polyaminecarboxylate chelators. (**A**) EDTA, and (**B**) DTPA

Another class of early copper chelators comprised acyclic polyaminecarboxylates, including EDTA and DTPA (Fig. 3). These ligands offer high thermodynamic stability due to multiple donor atoms and flexible coordination environments. However, their open-chain structures lack the rigidity needed to prevent metal dissociation [57, 66]. For copper radionuclides, which are prone to transchelation by endogenous proteins like albumin or superoxide dismutase, this insufficient kinetic inertness would potentially result in poor in vivo stability and rapid clearance from target tissues. This fact made them obsolete to integrate copper-based radiopharmaceuticals in recent years.

**Fig. 4:**
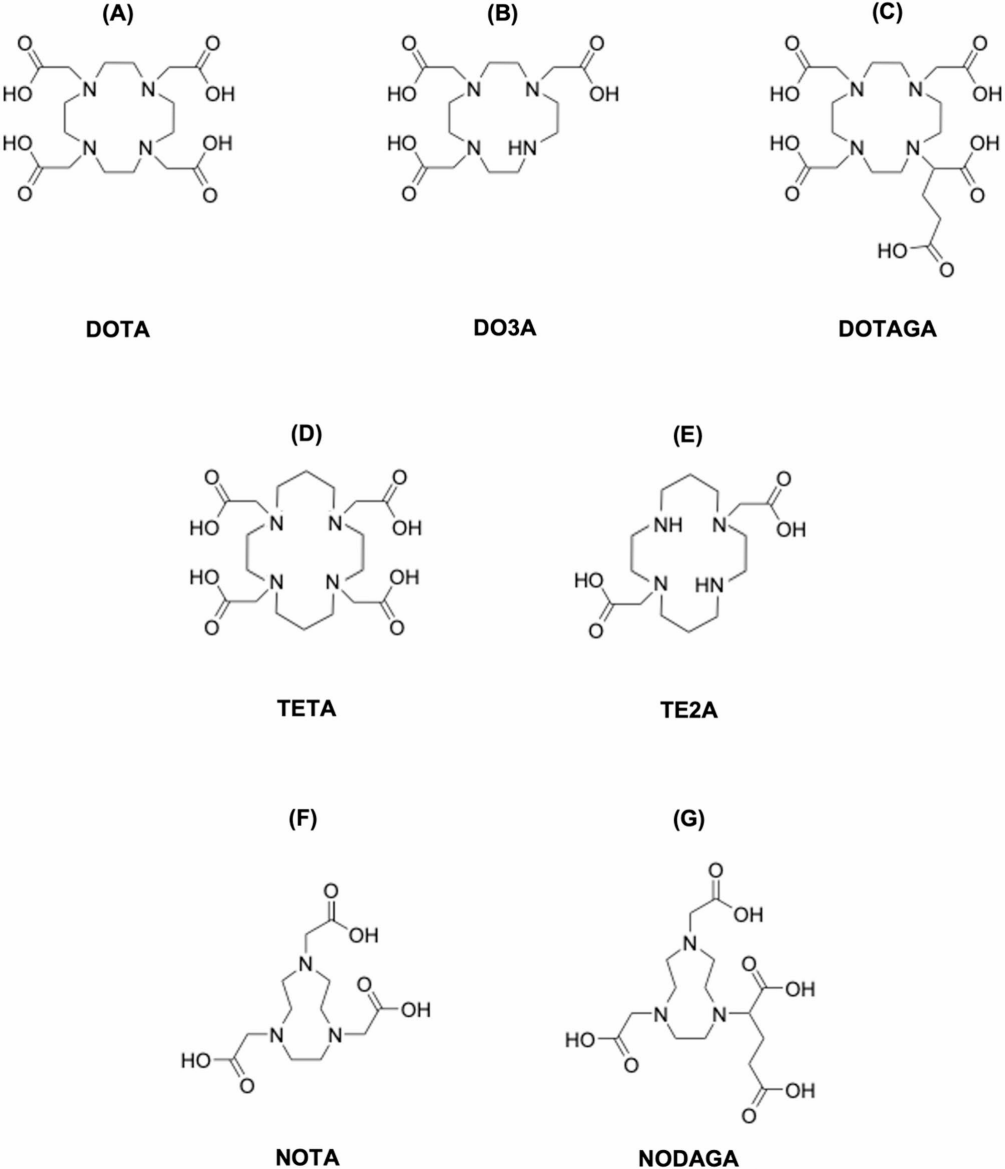
Schematic structure of classic macrocyclic polyaminecarboxylate chelators and their most common derivatives. (**A**) DOTA, (**B**) DO3A, (**C**) DOTAGA, (**D**) TETA, (**E**) TE2A, (**F**) NOTA, and (**G**) NODAGA

The shift toward macrocyclic polyaminecarboxylate chelators marked a significant improvement in radiometal complexation, with ligands such as DOTA, TETA, NOTA, and their derivatives (Fig. 4) providing preorganized donor frameworks that accommodate the coordination tendencies of Cu(II).

NOTA is characterized by a small, nine-membered macrocyclic cavity with three pendant acetate arms that is well-suited for accommodating small metal ions. However, in spite of its compact size, NOTA exhibits a degree of flexibility that further allows the coordination of larger ions [67]. In the case of Cu(II), the d^9^ electronic configuration induces a Jahn–Teller distortion, resulting in a distorted trigonal prismatic geometry and an irregular coordination sphere [67, 68]. This distortion leads to weak axial bonding, which facilitates protonation of the complex. Interestingly, the protonation of axial donor atoms enhances copper affinity, enabling rapid complexation even at room temperature and low pH [56, 67–70]. The formation of thermodynamically stable protonated species contributes to the robustness of the Cu(II)-NOTA complex. Compared to larger macrocycles, the cavity size and donor atom arrangement of NOTA better match the coordination requirements of Cu(II) [67], making it one of the most efficient chelators of its generation for integrating into copper-based radiopharmaceuticals.

On the other hand, DOTA, a member of the cyclen family, features a 12-membered macrocyclic ring with four pendant acetate arms. While its octadentate structure is highly effective for coordinating larger trivalent radiometals such as Lu(III) and Y(III) [71], its cavity is less compatible with smaller divalent ions, causing the copper atom to sit above the plane of the macrocyclic ring rather than within it [68, 72, 73]. This geometric mismatch leads to pseudotetrahedral or distorted octahedral geometries, reducing the overall stability of the complex [68, 72, 73]. As a result, DOTA requires thermal activation for efficient radiolabeling due to the slower coordination kinetics [68, 71], which may compromise the integrity of heat-sensitive biomolecules like antibodies and certain peptides. In addition, Cu(II) coordinates with two macrocyclic nitrogen atoms and two carboxylate oxygen atoms, leaving two pendant arms unbound. These uncomplexed arms may interact with competing Cu(II) ions, potentially leading to subsequent unwrapping of the chelator and transchelation of the radiometal [72, 73], which further contributes to the increased instability of DOTA-based radiopharmaceuticals.

TETA, part of the cyclam family, contains a 14-membered macrocyclic ring that offers a larger cavity than DOTA. This expanded core allows Cu(II) to sit more comfortably within the plane of the macrocycle, resulting in a more stable coordination geometry under milder conditions [68, 73]. Due to its in-core positioning, the metal ion is less exposed to external interactions in Cu(II)-TETA compared with Cu(II)-DOTA complexes. Nevertheless, despite its favorable geometry, Cu(II)-TETA complexes are still prone to relatively high copper dissociation in vivo owing to associative loss mechanisms sustained by the additional unbound carboxylate arms [68, 73, 74], limiting their long-term stability in biological systems.

**Fig. 5:**
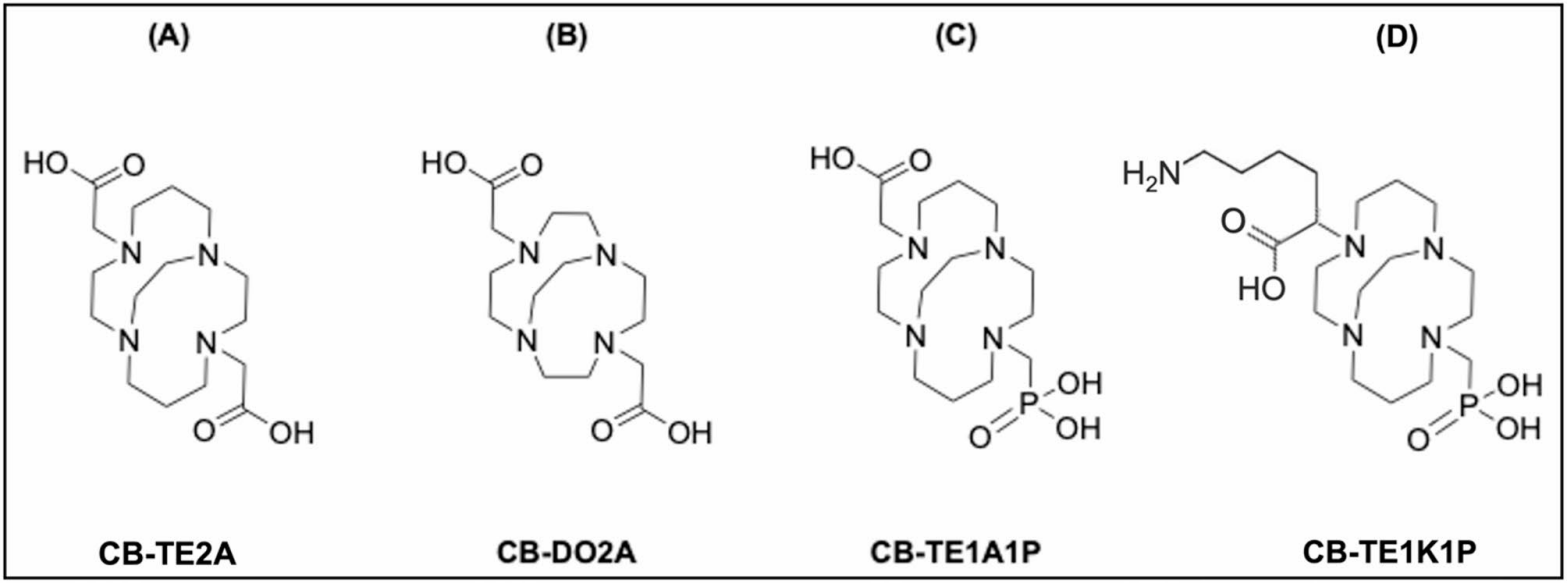
Schematic structure of cross-bridged macrocyclic chelators. (**A**) CB-TE2A, (**B**) CB-DO2A, (**C**) CB-TE1A1P, and (**D**) CB-TE1K1P

To further address these constraints, a third generation of cross-bridged macrocyclic chelators were developed, including CB-TE2A, CB-DO2A, CB-TE1A1P, and CB-TE1K1P (Fig. 5). These ligands feature rigid ethylene bridges across the macrocyclic ring that allow the cupric ion to be encapsulated within an exceptionally inert coordination sphere, significantly suppressing metal dissociation [75]. However, cross-bridged chelators are often disadvantaged by the harsh conditions (e.g., high temperature and long reaction times) required to achieve optimal radiolabeling yields [76], which can limit their applicability. CB-TE1A1P and CB-TE1K1P, featuring a phosphonate substitution for one carboxylic pendant arm, were subsequently introduced as an alternative to mitigate the challenges posed by the inconvenient radiolabeling demands of conventional cross-bridged systems [77–79].

**Fig. 6:**
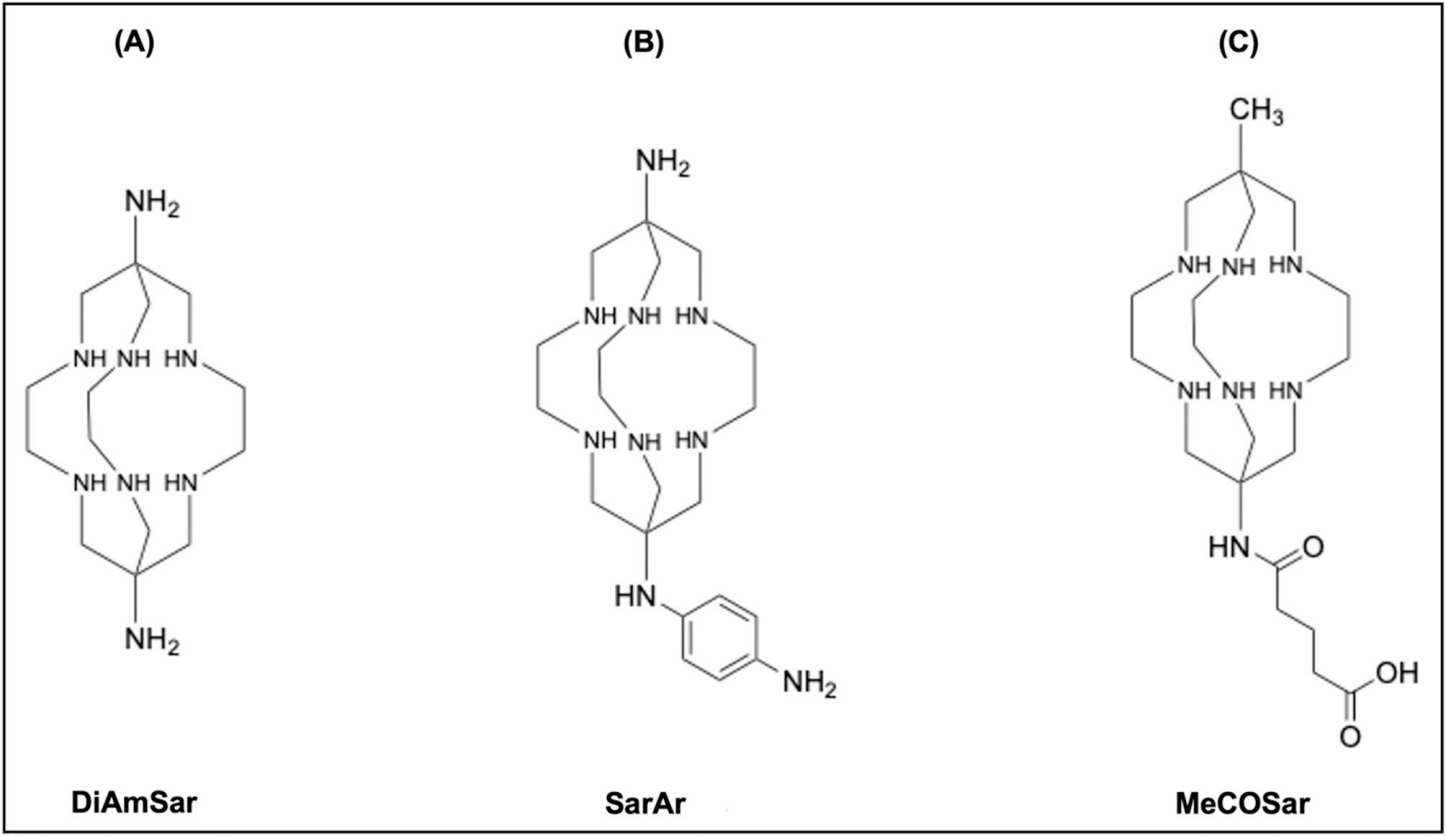
Schematic structure of sarcophagine-based chelators. (**A**) DiAmSar, (**B**) SarAr, and (**C**) MeCOSar

Recent innovations in copper chelation have driven the design of sarcophagine (SAR)-derived ligands, including DiAmSar, SarAr, and MeCOSar (Fig. 6), which incorporate a rigid hexamine cage structure that tightly encloses the copper ion. This architecture confers unparalleled kinetic inertness and thermodynamic stability, virtually eliminating transchelation under physiological conditions [72]. Another key advantage of sarcophagines lies in their extremely mild radiolabeling requirements, enabling rapid and efficient radiolabeling at room temperature [71].

## Molecular targets for prostate cancer theranostics

**Fig. 7:**
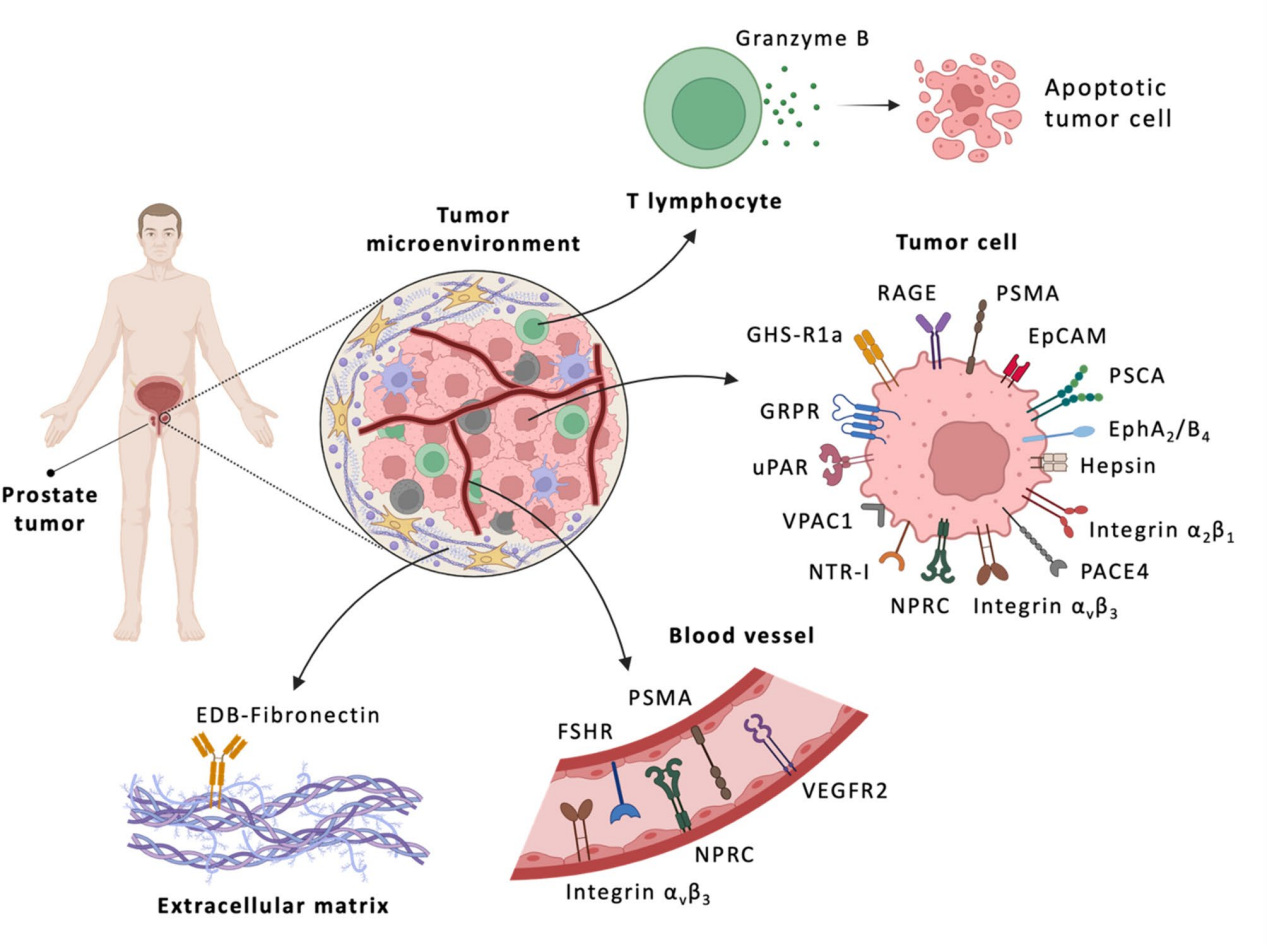
Schematic representation of key components of PCa tumor microenvironment (TME) and associated molecular targets. The figure illustrates major cellular and stromal structures within the TME that present a wide spectrum of promising avenues to be explored in the design of radiopharmaceuticals for both diagnostic and therapeutic intervention in PCa. Created with BioRender.com

As our understanding PCa biology continues to evolve, numerous molecular targets for imaging and therapy have been identified (Fig. 7), fueling the development of novel radiopharmaceuticals for PCa management. Among these, PSMA and gastrin-releasing peptide receptor (GRPR) are the most extensively studied. To date, only PSMA-targeted radiopharmaceuticals have received FDA approval, namely [^68^Ga]Ga-PSMA-11, [^18^F]DCFPyL (PYLARIFY^®^), and [^177^Lu]Lu-PSMA-617 (Pluvicto^®^). However, the challenges of tumor heterogeneity and therapeutic resistance emphasize the need to expand the molecular target repertoire and refine next-generation strategies for precision imaging and therapy.

This section provides a comprehensive overview of established and emerging targets for PCa theranostics, with particular focus on recent advances in copper-based radiopharmaceuticals, and summarizes key preclinical and clinical data.

### Prostate-specific membrane antigen

PSMA is a type II transmembrane glycoprotein that is consistently overexpressed in over 90% of PCa cases, particularly in advanced and metastatic stages, making it a prime target for imaging and therapy [80, 81]. Table 2 provides an overview of copper-based PSMA-targeted agents that have been evaluated in preclinical models or clinical settings, while Table 3 outlines related clinical trials.

The potential of PSMA as a molecular target for PCa was first established through immunoscintigraphy studies using the mAb 7E11 radiolabeled with indium-111, commercially known as ProstaScint^®^ and approved by the FDA in the late 1990s [82]. However, since ProstaScint^®^ binds to the intracellular domain of PSMA, only necrotic or apoptotic cells could be targeted, which significantly compromised its clinical utility. To overcome this limitation, mAbs that specifically recognize epitopes located within the extracellular domain of PSMA—including huJ591 [83], 3/A12, 3/F11, and 3E7 [84]—were developed and radiolabeled with copper-64 for preclinical PET imaging [85–88]. Interestingly, the ^64^Cu-labeled huJ591 and 3/F11 mAbs have also been investigated for Cerenkov luminescence imaging (CLI) in PCa xenografts as a low-cost alternative to immuno-PET [87, 88]. In addition, a Phase I clinical trial (NCT04726033) assessed the safety and dosimetry of [^64^Cu]Cu-TLX592 as a diagnostic surrogate to guide subsequent ^225^Ac-based targeted alpha therapy in early metastatic and late-stage PCa patients refractory to lutetium-177 treatment.

Full-length mAbs are often disadvantaged by prolonged circulation times and limited tumor penetration, which has led to an overall preference for smaller targeting constructs with improved pharmacokinetics. In this context, antibody fragments and low-molecular-weight ligands have also been investigated for PCa imaging and therapy in both preclinical and clinical settings. Some studies have explored the huJ591 minibody (Mb), and the 3/A12 F(ab)_2_ and Fab antibody fragments radiolabeled with copper-64 in preclinical models, comparing their pharmacokinetic profiles and tumor-targeting capacity to those of their corresponding parental mAbs [86, 87]. On a slightly different note, single chain fragment variable (scFv) and diabody fragments derived from the huJ591 mAb have been conjugated to lipid nanoparticles and radiolabeled with copper-64 for PET imaging, primarily to support nanoparticle-mediated targeted chemotherapy drug delivery [89, 90]. Conversely, Fletcher et al. designed a huJ591-based antibody construct with impaired FcRn recycling to reduce circulation time. When radiolabeled with copper-64, it retained PSMA affinity, and showed rapid blood clearance and selective tumor accumulation [91]. Two additional studies have reported the use of poly(amidoamine) dendrimer nanoparticles functionalized with Lys-urea-Glu scaffolds for PSMA targeting, NOTA chelators for copper-64 conjugation, and fluorescent dyes for optical imaging [92, 93] [93]. These ligands demonstrated selective tumor accumulation, efficient cellular internalization, and minimal off-target uptake. One of the constructs was further modified with the cytotoxic agent DM1 for therapeutic evaluation, leading to effective tumor growth inhibition with no signs of systemic toxicity [93]. A clinical trial (NCT04167969) is also ongoing to evaluate [^64^Cu]Cu-NOTA-PSMA-PEG-Cy5.5-C’, a nanoparticle-based radiopharmaceutical for PET/MR imaging and potential intraoperative use to enhance lesion detection.

Aside from the mentioned studies, most preclinical and clinical research has centered on small-molecule PSMA inhibitors, mainly Lys-urea-Glu peptidomimetic scaffolds, due to their favorable pharmacokinetic and biodistribution profiles, efficient tumor penetration, high specificity, low immunogenicity, and ease of chemical synthesis and radiolabeling.

As shown in Table 2, many copper-based PSMA-targeted radiopharmaceuticals employ DOTA or DOTAGA chelators, including the well-established urea-based ligands PSMA-617 and PSMA-I&T. However, as explained before, these chelators often exhibit suboptimal in vivo stability when complexed with copper, largely due to transchelation to serum proteins, leading to off-target accumulation, particularly in the liver.

**Fig. 8:**
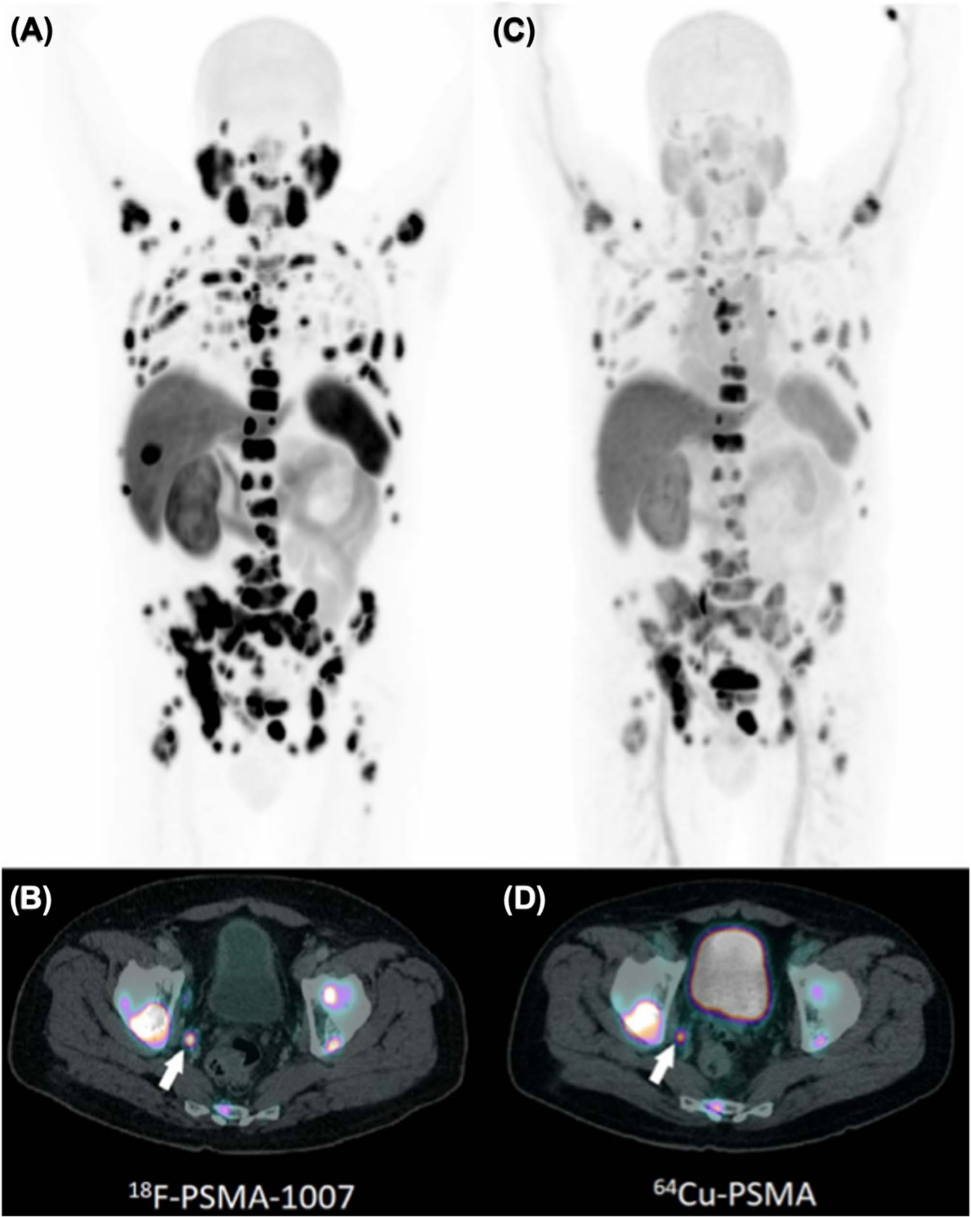
Comparison of PSMA-targeted PET/CT in metastatic PCa. Panels (**A**) (maximum-intensity projection) and (**B**) (fused axial PET/CT) show [^18^F]PSMA-1007 images of a 68-year-old man (Gleason score 9; PSA 1044 ng/mL). Panels (**C**) (maximum-intensity projection) and (**D**) (fused axial PET/CT) display corresponding [^64^Cu]Cu-PSMA images, revealing tracer uptake in identical skeletal metastases. High uptake in a right internal iliac lymph node is indicated by arrows in panels (B) and (D). Reproduced with permission from Cardoza-Ochoa et al. [101]

Notwithstanding the constraints related to copper chelation, [^64^Cu]Cu-PSMA-617 has demonstrated good imaging performance. In a first in-human study, PET scans enabled clear lesion delineation in patients with recurrent PCa or progressive local disease, with high tumor-to-background contrast as early as 1 h post-injection [94]. Additional clinical investigations have further substantiated its diagnostic potential across various PCa stages and clinical scenarios. In two studies by Cantiello et al., [^64^Cu]Cu-PSMA-617 PET/CT showed high accuracy for primary lymph node staging in intermediate- to high-risk patients scheduled for radical prostatectomy [95], and outperformed [^18^F]F-choline in restaging PCa after BCR—particularly in patients with low PSA levels [96]. Additional case reports include detection of a thyroid metastasis in castration-resistant PCa [97], and identification of a rare soft tissue penile metastasis [98]. The physiological behavior and pharmacokinetics of [^64^Cu]Cu-PSMA-617 have also been evaluated. Calabria et al. compared its biodistribution with that of [^68^Ga]Ga-PSMA-11, noting similar uptake patterns but distinct excretion pathways—biliary for [^64^Cu]Cu-PSMA-617 versus renal for [^68^Ga]Ga-PSMA-11 [99]. In another study, Hoberück et al. found that early imaging provided sufficient lesion visualization, with no added clinical benefit at delayed time points. They also observed a time-dependent redistribution of physiological uptake, with decreasing signal in the kidneys, bladder and salivary glands, and increasing hepatic and intestinal activity [100]. Finally, a small comparative study by Cardoza-Ochoa et al. [101] demonstrated that [^64^Cu]Cu-PSMA-617 and [^18^F]PSMA-1007 offered comparable image quality and lesion detection (Fig. 8). Complementing these clinical insights, a preclinical study introduced a modular probe structurally analogous to PSMA-617, designed for flexible labeling with fluorine-18 or radiometals such as copper-64 or lutetium-177. PET/CT imaging in PSMA-expressing xenografts showed consistent tumor uptake across tracers—^18^F/^nat^Cu and ^nat^F/^64^Cu isotopologs, and ^18^F/free—although the ^nat^F/^64^Cu-labeled version exhibited significant hepatobiliary accumulation, contrasting with the negligible liver uptake seen for the other variants [102].

**Fig. 9:**
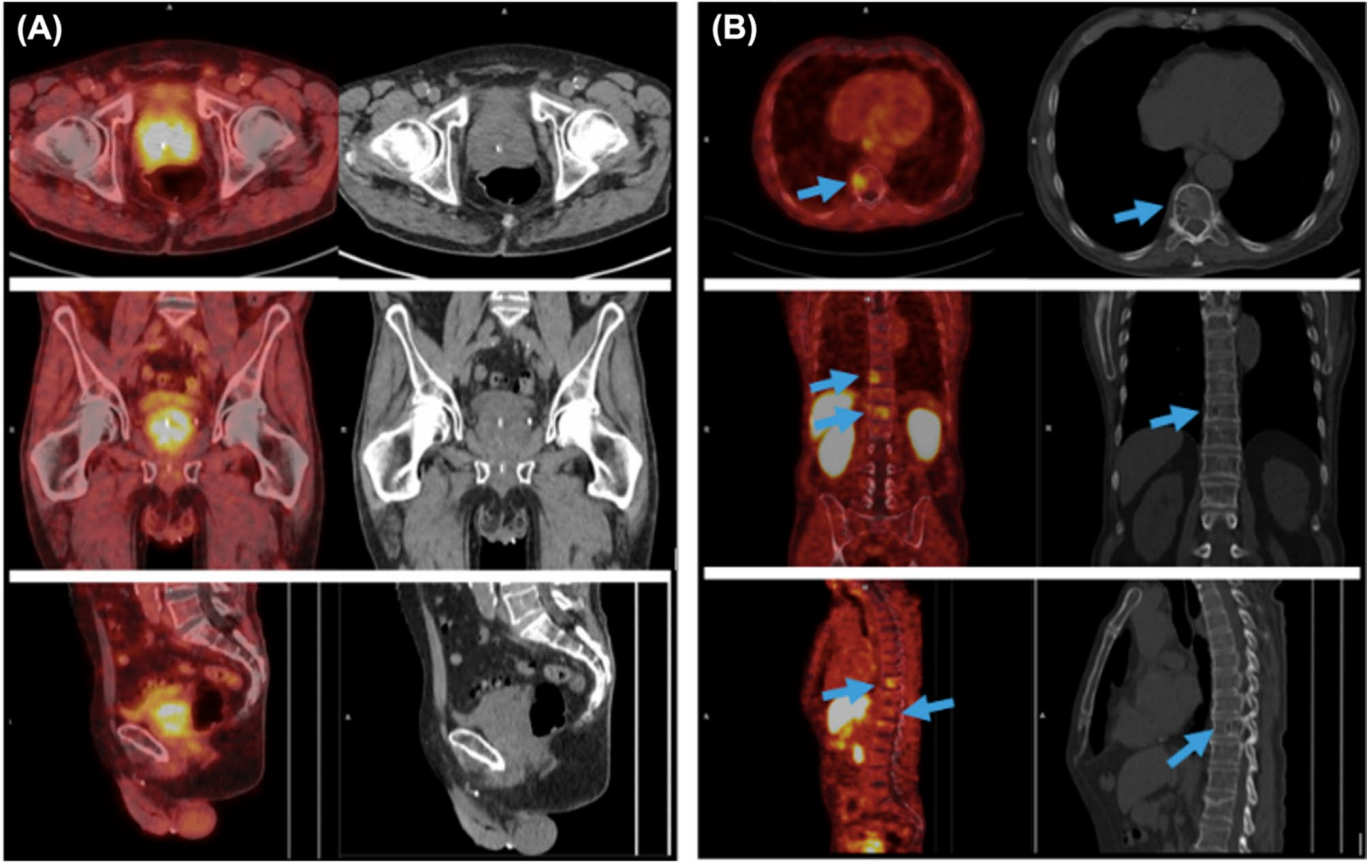
Primary staging of a treatment-naïve 75-year-old PCa patient with bone metastases, imaged using [^64^Cu]Cu-DOTAGA-PSMA PET/CT. At the time of imaging, PSA was 47 µg/L. (**A**) PSMA-positive primary tumor (SUV_max apical: 9.5; left base: 8.0). (**B**) PSMA-avid bone metastases in T10 and T12 (SUV_max: 5.3 and 4.6, respectively); arrow in low-dose CT image indicates the lesion in T10. Reproduced from Mirzaei et al. [103], licensed under CC BY 4.0 (https://creativecommons.org/licenses/by/4.0/)

**Fig. 10:**
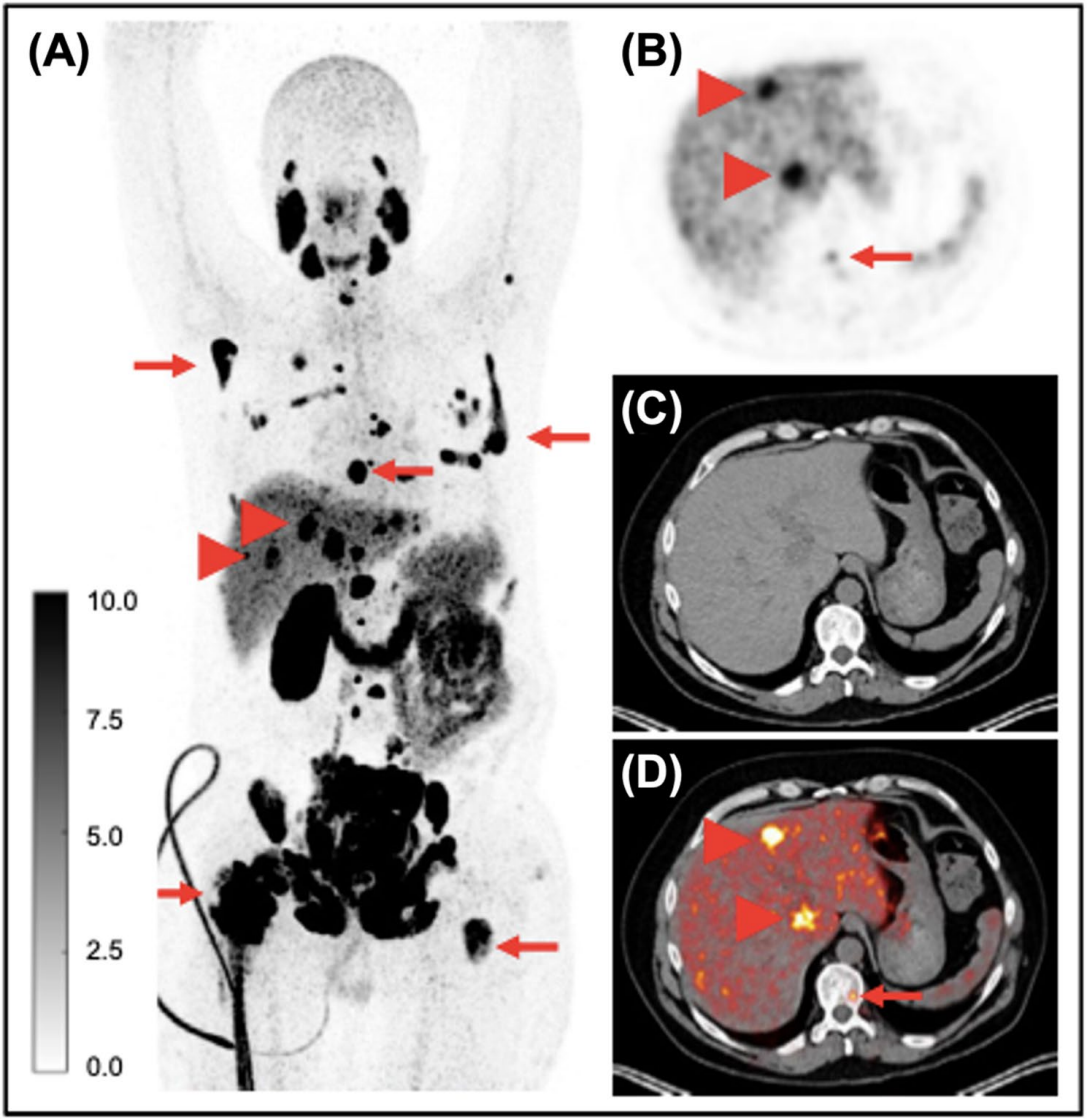
PET/CT images acquired 3 h after administration of [^61^Cu]Cu-NODAGA-PSMA-I&T in a 48-year-old patient with metastatic castration-resistant PCa, following disease progression after abiraterone and docetaxel treatment and prior to [^177^Lu]-PSMA radioligand therapy. Maximum-intensity projection (**A**), PET (**B**), CT (**C**), and fused PET/CT (**D**) images show multiple osseous metastases (arrows) and hepatic metastases (arrowheads). The patient had a history of left nephrectomy. Reproduced from Bernabeu et al. [106], licensed under CC BY 4.0 (https://creativecommons.org/licenses/by/4.0/)

Beyond PSMA-617, additional studies have focused on PSMA-I&T and related ligands. In a single-center clinical study, Mirzaei et al. compared [^64^Cu]Cu-DOTAGA-PSMA (Fig. 9) and [^18^F]PSMA PET/CT in patients with recurrent or locally advanced PCa [103]. Detection rates were similar for both radioconjugates, but, unexpectedly, [^18^F]PSMA exhibited higher liver uptake. The imaging performance of [^64^Cu]Cu-PSMA-I&T has also been evaluated in two preclinical studies. Lee et al. demonstrated high specificity and clear tumor visualization [104], while Nelson et al. reported stronger tumor uptake compared to [^68^Ga]Ga-PSMA-I&T, with signal retention up to 48 h post-injection [105]. Both studies reported significant liver accumulation. The newcomer copper-61 has also been used to radiolabel PSMA-targeted molecules, offering an alternative to copper-64 with potentially favorable imaging characteristics. A recent study compared [^61^Cu]Cu-NODAGA-PSMA-I&T and [^61^Cu]Cu-DOTAGA-PSMA-I&T, with the NODAGA-conjugated radioligand showing higher tumor uptake, lower background activity, and reduced nonspecific accumulation in the liver and abdominal region [106]. Its biodistribution closely aligned with that of [^68^Ga]Ga-PSMA-11 and [^18^F]PSMA-1007, while enabling delayed imaging for enhanced contrast. In a preliminary first in-human evaluation, [^61^Cu]Cu-NODAGA-PSMA-I&T successfully identified osseous and hepatic metastases in a patient with metastatic castration-resistant PCa (Fig. 10), supporting its clinical potential. Accordingly, these findings led to a Phase I clinical trial (NCT06736054) aiming to assess the safety and diagnostic performance of this novel radiotracer against the FDA-approved [^18^F]DCFPyL. The results obtained by Bernabeu et al. were further validated in our group, who reported the fully-automated, GMP-compliant synthesis and preclinical evaluation of [^61^Cu]Cu-NODAGA-PSMA-I&T and [^61^Cu]Cu-DOTAGA-PSMA-I&T [107]. Bunda et al. expanded the investigations on copper-61 by introducing [^61^Cu]Cu-KFTG-PSMA, a novel radioconjugate incorporating the recently developed KFTG chelator. In vivo PET imaging revealed progressive tumor uptake, low background activity, and improved contrast at later time points. Ex vivo biodistribution confirmed this time-dependent tumor accumulation, and blocking studies with cold PSMA demonstrated high target specificity [108].

Despite its well-known in vivo instability, [^64^Cu]Cu-PSMA-I&T has progressed to clinical evaluation through the SOLAR program, which includes multiple studies. SOLAR-Stage (NCT06235151) is investigating its diagnostic accuracy for primary staging in newly diagnosed intermediate- to high-risk PCa patients undergoing radical prostatectomy. SOLAR-Recur (NCT06235099) focuses on detecting BCR following definitive treatment. Meanwhile, the SOLAR Phase I/II study (NCT05653856) has already reported encouraging results, including safety, favorable biodistribution, and high detection rates in metastatic PCa patients.

**Fig. 11:**
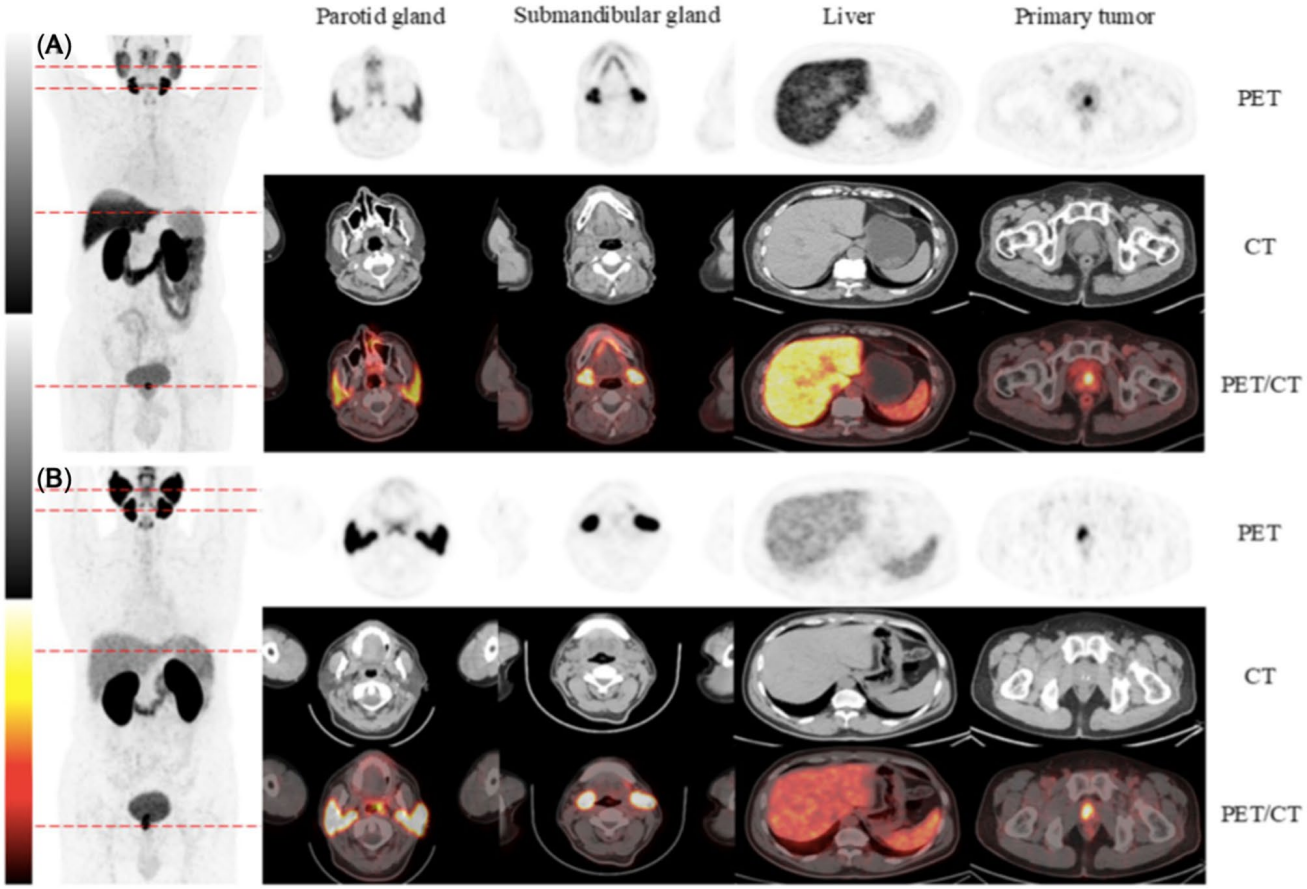
Representative PET/CT images acquired 2 h post-injection of ^64^Cu-labeled PSMA ligands in two patients with primary PCa. Panel (**A**) shows MIP, low-dose axial CT, and fused PET/CT images of an 80-year-old patient (PSA 10.3 ng/mL, Gleason score 4 + 3) after administration of [^64^Cu]Cu-DOTA-PSMA-3Q, with notable uptake in the parotid gland (SUV_max 7.49), submandibular gland (12.97), liver (8.09), spleen (4.36), and primary tumor (14.87). Panel (**B**) shows corresponding images from a 65-year-old patient (PSA 18.49 ng/mL, Gleason score 4 + 3) imaged with [^64^Cu]Cu-NOTA-PSMA-3Q, demonstrating uptake in the parotid gland (19.83), submandibular gland (24.97), liver (4.57), spleen (4.76), and primary lesion (14.46). Reproduced from Liu et al. [110], licensed under CC BY-NC-ND 4.0 (https://creativecommons.org/licenses/by-nc-nd/4.0/)

Two recent studies have investigated a novel radioligand, [^64^Cu]Cu-DOTA-PSMA-3Q, in preclinical and clinical settings. Zhang et al. introduced its use for both PET/CT imaging and radiation-guided prostate biopsy, where intraoperative gamma spectroscopy allowed real-time quantification of radioactivity in biopsy cores and immediate lesion verification, aiming to improve diagnostic accuracy. However, liver uptake was notable [109]. In light of this suboptimal hepatic accumulation, Liu et al. subsequently explored [^64^Cu]Cu-NOTA-PSMA-3Q, which showed comparable safety and diagnostic value but with significantly lower liver uptake compared to its DOTA analogue (Fig. 11) [110]. In a separate study, Chen et al. evaluated [^64^Cu]Cu-PSMA-Q, also using a DOTA chelator, and reported higher tumor-to-background ratios and superior lesion detection compared to [^18^F]FDG in a clinical cohort [111]. This enhanced sensitivity was further illustrated by Bartholomä et al., who reported a case of BCR where [^18^F]DCFPyL PET/CT showed only faint uptake near the bladder neck, while follow-up imaging with a ^64^Cu-labeled trimeric PSMA ligand revealed clear prostate bed uptake, confirming the presence of a small lesion and enabling targeted local therapy [112].

Lim et al. investigated the impact of molecular size on multivalent PSMA targeting by using a DOTA-conjugated DUPA scaffold linked to triazine dendrimers of varying generations. Among the constructs tested, G1-(DUPA)_4_ showed superior performance with high tumor-to-background ratios and clear delineation of PSMA-positive xenografts. In contrast, larger dendrimers exhibited increased off-target uptake in the liver and heart, likely due to prolonged circulation times [113].

As discussed throughout this review, the in vivo stability of copper-labeled conjugates largely depends on the strength of the copper-chelator coordination complex. To address the limitations of DOTA and DOTAGA, several alternative chelators have been evaluated, including cross-bridged frameworks, SAR-based systems, NOTA derivatives, and modified DOTA analogs.

Banerjee et al. demonstrated that ^64^Cu-labeled compounds using CB-TE2A and NOTA chelators provided superior stability and favorable biodistribution in preclinical models, with the CB-TE2A complex offering the best image contrast [114]. Similar conclusions were drawn by Gourni et al. [115] and Lappchen et al. [116], who evaluated R-NODAGA and NODIA-Me chelators, respectively, both yielding low liver uptake and efficient renal clearance. Umbricht et al. further confirmed these trends using albumin-binding NODAGA-functionalized ligands, reporting superior tumor uptake and markedly lower liver accumulation for [^64^Cu]Cu-PSMA-ALB-89 related to its DOTA-based counterpart [117]. Lee et al. also observed lower hepatic uptake and greater tumor accumulation with NOTA-conjugated PSMA ligands versus DOTA analogs [118]. Nedrow et al. investigated a CB-TE1K1P-chelated phosphoramidate inhibitor radiolabeled with copper-64, demonstrating high tumor uptake which correlated with PSMA expression, favorable pharmacokinetics, and excellent image contrast [79]. Lewis et al. introduced a bimodal PSMA-targeted agent radiolabeled with copper-64/67 and functionalized with a near-infrared dye for combined PET imaging, image-guided surgical navigation, and TRT. Based on the NODAGA chelator, the radiopharmaceutical showed high specificity for PSMA-positive tumors with negligible uptake in PSMA-negative models, supporting its utility for concomitant imaging and therapy [119]. In parallel, Milot et al. investigated the DOTHA_2_ chelator for both imaging [120] and therapeutic [121] applications. [^64^Cu]Cu-DOTHA_2_-PSMA demonstrated excellent tumor targeting and stability, with sustained uptake up to 24 h post-injection. Therapeutic studies showed efficacy comparable to [^177^Lu]Lu-PSMA-617, though hepatic and gastrointestinal irradiation may pose limitations.

Hao et al. focused on overcoming the phosphate-mediated interference that limits the in vivo performance of GPI-based PSMA inhibitors. Using a ^64^Cu-specific bifunctional chelator scaffold, they synthesized monomeric and dimeric conjugates, with the dimeric form, [^64^Cu]Cu-CBT2G, showing superior tumor uptake and retention. Both constructs demonstrated rapid renal clearance and minimal off-target accumulation, validating the multivalent approach as a strategy to restore the in vivo utility of otherwise compromised PSMA-targeting agents [122]. On the other hand, Harmatys et al. proposed an alternative theranostic approach using LC-Pyro, a ^64^Cu-labeled porphyrin-based PSMA-targeted agent designed for dual PET/fluorescence imaging and photodynamic therapy. The incorporation of a peptide linker prolonged circulation time, resulting in high tumor accumulation and excellent tumor-to-background ratios. LC-Pyro also enabled selective imaging of PSMA-positive lesions and significantly delayed tumor growth following light activation [123].

**Fig. 12:**
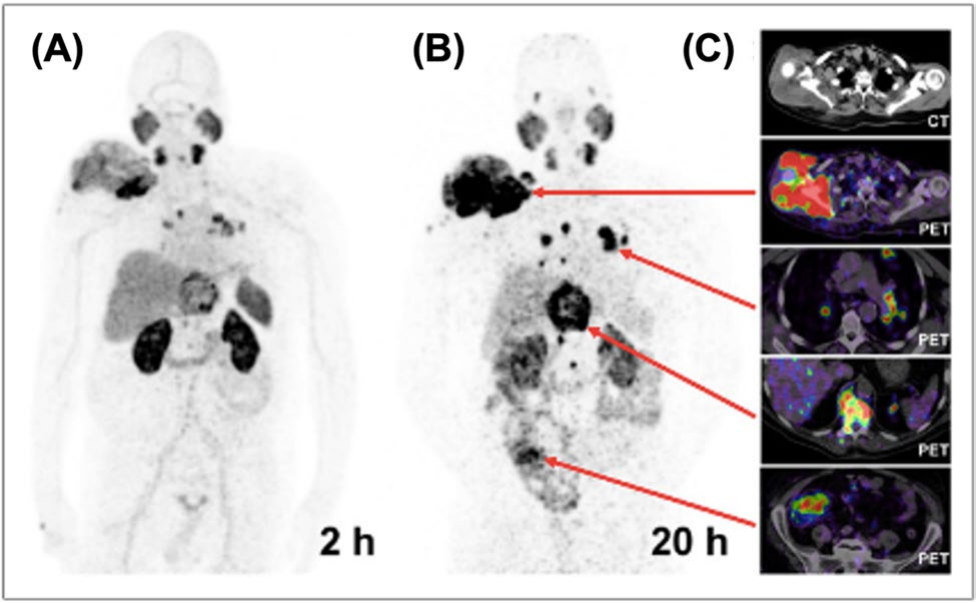
Maximum-intensity projection PET/CT images acquired at 2 h (**A**) and 20 h (**B**) post-injection of [^64^Cu]Cu-CA003 (200 MBq, 0.5 nmol) show uptake in right shoulder soft-tissue infiltration originating from the scapula, as well as in pulmonary, osseous, and nodal metastases, with increasing contrast over time. Hepatobiliary excretion results in visible intestinal activity in delayed imaging. Cross-sectional views (**C**) are essential to accurately interpret tracer distribution and prevent false-positive findings. Reproduced from Dos Santos et al. [125]

**Fig. 13:**
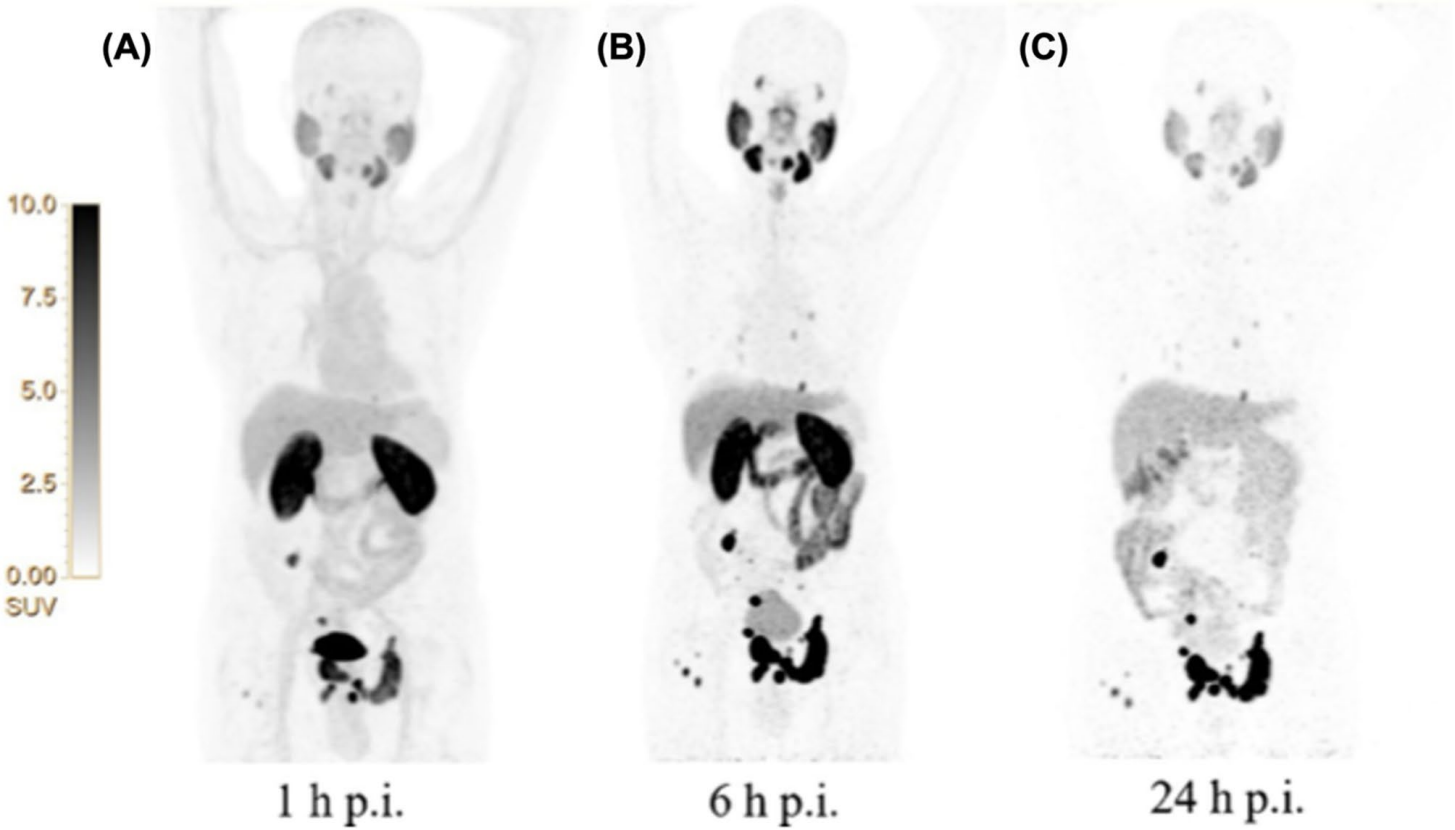
Maximum-intensity projection PET images acquired at 1 h (**A**), 6 h (**B**), and 24 h (**C**) after injection of [^64^Cu]Cu-PSMA-BCH in an 80-year-old patient with a PSA level of 11.80 ng/mL and Gleason score 4 + 4. Reproduced from Liu et al. [126], licensed under CC BY-NC-ND 4.0 (https://creativecommons.org/licenses/by-nc-nd/4.0/)

Notably, a growing number of studies have progressed to clinical evaluation. Sevcenco et al. retrospectively assessed [^64^Cu]Cu-NODAGA-PSMA PET/CT for primary staging of PCa patients. Uptake in the prostate bed, lymph nodes, and distant metastases correlated significantly with PSA levels and increased over time, indicating in vivo stability and potential for detecting residual or recurrent cancer [124]. Dos Santos et al. [125] reported encouraging first-in-human results with [^64^Cu]Cu-CA003, a TETA-derived PSMA ligand, which showed high tumor uptake and contrast with rapid renal clearance (Fig. 12). Likewise, Liu et al. [126] evaluated [^64^Cu]Cu-PSMA-BCH in four patients, showing high specificity, low liver uptake, and improved lesion detection on delayed imaging (Fig. 13).

Among the most impactful contributions to copper-based radiopharmaceutical development are the works led by Paul S. Donnelly, which laid the foundation for the use of SAR-based chelators in PCa imaging and therapy. In a pivotal preclinical study, Zia et al. compared monovalent and bivalent SAR-chelated ligands bearing Lys-urea-Glu moieties, showing that the bivalent construct exhibited superior tumor uptake and retention, with excellent image contrast up to 24 h post-injection [127]. These results prompted a subsequent study by McInnes et al., who demonstrated that [^67^Cu]Cu-SAR-bisPSMA achieved therapeutic outcomes comparable to [^177^Lu]Lu-PSMA-I&T, with both single and fractionated administrations leading to significant tumor growth inhibition and improved survival [128]. Building on this concept, Kelly et al. developed [^64^Cu]Cu-RPS-085, an agent that combines SAR-based chelation, PSMA targeting, and albumin-binding functionality. The radiopharmaceutical showed high and sustained tumor uptake, rapid renal clearance, minimal background activity, and excellent tumor-to-background ratios [129]. These robust preclinical findings have catalyzed a series of clinical trials. The PROPELLER trial (NCT04839367), a Phase I study in newly diagnosed PCa patients, confirmed safety and showed higher lesion uptake of [^64^Cu]Cu-SAR-bisPSMA compared to [^68^Ga]Ga-PSMA-11 [130]. This led to the Phase III CLARIFY trial (NCT06056830), designed to assess the ability of [^64^Cu]Cu-SAR-bisPSMA to detect nodal metastases in high-risk patients [131]. In the BCR setting, the Phase I/II COBRA trial (NCT05249127) reported detection in up to 80% of patients previously negative on standard imaging, with next-day scans nearly doubling the number of detected lesions compared to same-day imaging [132]. This body of work informed the AMPLIFY trial, a second Phase III study for BCR patients, with recruitment expected to begin in late 2025 [133]. In parallel, the SECuRE trial (NCT04868604) expanded the clinical program into the theranostic realm by combining [^64^Cu]Cu-SAR-bisPSMA for imaging and [^67^Cu]Cu-SAR-bisPSMA for therapy in metastatic castration-resistant PCa. In Phase I, [^67^Cu]Cu-SAR-bisPSMA was well tolerated with no dose-limiting toxicity [134]. Among 13 chemotherapy-naive patients, 12 experienced PSA reductions ≥ 35%, and nearly half achieved reductions ≥ 80%, most after a single dose [135]. Notably, a complete response was observed in the first patient to receive two 8 GBq cycles [136]. Phase II is currently ongoing [137]. Finally, the recently-initiated Phase II Co-PSMA trial (NCT06907641) is comparing [^64^Cu]Cu-SAR-bisPSMA and [^68^Ga]Ga-PSMA-11 for recurrence detection in patients eligible for salvage radiotherapy [138, 139].

**Table 2:** Overview of published studies on copper-based radiopharmaceuticals directed at PSMA for PCa imaging and therapy across preclinical and clinical settings, presented in chronological order

Radionuclide(s)	Chelator(s)	Targeting agent(s)	Target(s)	Study design	Cell line(s)	Mouse strain	Reference(s)	Year of publication
^64^Cu	DOTA	3/A12	PSMA	Preclinical	C4-2 and DU145	♂ SCID	Elsasser-Beile	2009
^64^Cu	DOTA	3/A12, 3/F11, 3/E7, 3/A12 F(ab)_2_, and 3/A12 Fab	PSMA	Preclinical	C4-2 and DU145	♂ SCID	Alt	2010
^64^Cu	CB-TE2A-based	GPI	PSMA	Preclinical	LNCaP, PC-3, and H2009	♂ SCID	Hao	2013
^64^Cu	NOTA, PCTA, Oxo-DO3A, CB-TE2A and DOTA	Lys-urea-Glu	PSMA	Preclinical	PC-3-PIP and PC-3-Flu	NOD/SCID	Banerjee	2014
^111^In, ^68^Ga, and ^64^Cu	(R)-NODAGA(tBu)_3_	Lys-urea-Glu	PSMA	Preclinical	LNCaP	♀ athymic Balb/C nude	Gourni	2015
^64^Cu	DOTA	PSMA-617	PSMA	Clinical	–	–	Grubmuller	2016
^64^Cu	CB-TE1K1P	CTT-1297	PSMA	Preclinical	LNCaP-LP, 22Rv1, C4-2B, LNCaP-EP, and PC-3	♂ NCr nude	Nedrow	2016
^64^Cu	DOTA	PSMA-617	PSMA	Clinical	–	–	Cantiello	2017
^68^Ga and ^64^Cu	DOTA	PSMA-617	PSMA	Preclinical	LNCaP and PC-3	♂ BALB/c *nu*/*nu*	Cui	2017
^64^Cu	NODAGA	huJ591 and huJ591 Mb	PSMA	Preclinical	22Rv1 and PC-3	♂ athymic NCr *nu/nu*	D’Souza	2017
^64^Cu	DOTA	huJ591 scFv-LNPs	PSMA	Preclinical	LNCaP	♂ NOD SCID	Wong	2017
^64^Cu	DOTA	huJ591 DB-LNPs	PSMA	Preclinical	LNCaP	♂ NOD/SCID	Wong	2017
^18^F and ^64^Cu	DOTA	PSMA-617	PSMA	Clinical	–	–	Cantiello	2018
^64^Cu	Porphyrin	Urea-based	PSMA	Preclinical	PC-3-PIP, PC-3-Flu, PC-3-ML-1124, and PC-3-ML-1117	♂ athymic nude	Harmatys	2018
^64^Cu	NODIA-Me	Lys-urea-Glu	PSMA	Preclinical	LNCaP	♀ athymic Balb/c nude	Lappchen	2018
^64^Cu	NODAGA	n.s.	PSMA	Clinical	–	–	Sevcenco	2018
^64^Cu	DOTA and NODAGA	PSMA-ALB-56 and PSMA-ALB-89	PSMA	Preclinical	PC-3-PIP and PC-3-Flu	♀ athymic Balb/c nude	Umbricht	2018
^64^Cu	n.s.	n.s.	PSMA	Clinical	–	–	Annovazzi	2019
^68^Ga and ^64^Cu	DOTA	PSMA-11 and PSMA-617	PSMA	Clinical	–	–	Calabria	2019
^64^Cu	DOTA	PSMA-617	PSMA	Clinical	–	–	Hoberuck	2019
^64^Cu	DOTA	G4(MP-KEU)	PSMA	Preclinical	PC-3-PIP and PC-3-Flu	♂ NOD-SCID	Lesniak	2019
^64^Cu	DOTA	DUPA	PSMA	Preclinical	PC-3-PIP and PC-3-Flu	♂ SCID	Lim	2019
^64^Cu	NODAGA	3/F11	PSMA	Preclinical	C4-2 and DU145	♂ SCID	Maier	2019
^64^Cu	MeCOSar and bisCOSar	Lys-urea-Glu	PSMA	Preclinical	LNCaP	♂ Balb/c nude	Zia	2019
^64^Cu	TETA-based and CB-TE2A-based	Lys-urea-Glu	PSMA	Preclinical and clinical	C4-2	♂ BALB/c *nu*/*nu*	Dos Santos	2020
^64^Cu	MeCOSar	Lys-urea-Glu	PSMA	Preclinical	LNCaP	♂ athymic Balb/c nu/nu	Kelly	2020
^18^F and ^64^Cu	DOTA	AMBF_3_-PSMA(−617)	PSMA	Preclinical	LNCaP	See Suppl	Lepage	2020
^64^Cu	DOTA	PSMA-617	PSMA	Clinical	–	–	Farhan	2021
^64^Cu	NOTA	PSMA-BCH	PSMA	Preclinical and clinical	22Rv1 and PC-3	♂ BALB/c nude	Liu	2021
^67^Cu and ^177^Lu	bisCOSar and DOTAGA	Lys-urea-Glu and PSMA-I&T	PSMA	Preclinical	LNCaP	♂ NSG	McInnes	2021
^18^F and ^64^Cu	DOTAGA	PSMA-1007 and PSMA-I&T	PSMA	Clinical	–	–	Mirzaei	2021
^18^F and ^64^Cu	DOTA	PSMA-1007 and PSMA-617	PSMA	Clinical	–	–	Cardoza-Ochoa	2022
^64^Cu	DOTAGA	PSMA-I&T	PSMA	Preclinical	22Rv1 and PC-3	n.s.	Lee	2022
^64^Cu	DOTHA_2_	Lys-urea-Glu	PSMA	Preclinical	LNCaP	NRG	Milot	2022
^64^Cu	DOTA and NOTA	Dipep	PSMA	Preclinical	PC-3-PIP and PC-3-Flu	♂ athymic nude BALB/c	Lee	2023
^64^Cu and ^177^Lu	DOTHA_2_ and DOTA	Lys-urea-Glu and PSMA-617	PSMA	Preclinical (Therapy)	LNCaP	NRG	Milot	2023
^61^Cu, ^68^Ga, and ^18^F	DOTAGA and NODAGA	PSMA-I&T, PSMA-11, and PSMA-1007	PSMA	Preclinical and clinical	LNCaP	♂ athymic nude *Foxn1*^*nu*^/*Foxn1*^*+*^	Bernabeu	2024
^64^Cu	NOTA	PT-DDC	PSMA	Preclinical (Imaging and Therapy)	PC-3-PIP and PC-3-Flu	♂ NOD-SCID	Lesniak	2024
^64^Cu and ^67^Cu	NODAGA	sCy7.5-PSMAi	PSMA	Preclinical	PC-3-PIP and PC-3	ICR or CB.17 SCID	Lewis	2024
^64^Cu	DOTAGA	PSMA-I&T	PSMA	Preclinical	LNCaP	♂ BALB/c nude	Nelson	2024
^18^F and ^64^Cu	n.s.	DCFPyL and trimeric PSMA ligand	PSMA	Clinical	–	–	Bartholomä	2025
^61^Cu	KFTG	Lys-urea-Glu	PSMA	Preclinical	LNCaP	♂ CB17 SCID	Bunda	2025
^64^Cu	DOTA	PSMA-Q	PSMA	Preclinical	LNCaP C4-2B and PC-3	Balb/c nude	Chen	2025
^64^Cu	DOTA	ANT4044	PSMA	Preclinical	LNCaP	♂ Balb/c nude	Fletcher	2025
^64^Cu	DOTA and NOTA	PSMA-3Q	PSMA	Preclinical and clinical	22Rv1	♂ BALB/c nude	Liu	2025
^61^Cu	DOTAGA and NODAGA	PSMA-I&T	PSMA	Preclinical	LNCaP	♂ athymic Swiss nude	Rodrigues	2025
^64^Cu	DOTA	PSMA-3Q	PSMA	Preclinical and clinical	22Rv1 and PC-3	♂ BALB/c nude	Zhang	2025

The coding distinguishes between mAbs, mAb fragments, peptidomimetic small molecules, phosphoramidate-based small molecules, and nanoparticles

**Table 3:** Summary of completed and ongoing clinical trials exploring PSMA-targeted, copper-based radiopharmaceuticals for PCa imaging and therapy, organized by the corresponding start date

Radiopharmaceutical	Intervention	Phase	Sponsor	Starting date	Status	Name (ID)
[^64^Cu]Cu-NOTA-PSMAi-PEG-Cy5.5-C’	Imaging	I	Memorial Sloan Kettering Cancer Center	02/2021	Recruiting	(NCT04167969)
[^64^Cu]Cu-Sar-bis-PSMA	Imaging	I	Clarity Pharmaceuticals Ltd	07/2021	Completed	PROPELLER (NCT04839367)
[^64^Cu]Cu-TLX592	Imaging	I	Telix Pharmaceuticals (Innovations) Pty Limited	08/2021	Completed	CUPID (NCT04726033)
[^64^Cu]Cu-Sar-bis-PSMA [^67^Cu]Cu-Sar-bis-PSMA	Imaging/Therapy	I/IIa	Clarity Pharmaceuticals Ltd	08/2021	Recruiting	SECuRE (NCT04868604)
[^64^Cu]Cu-Sar-bis-PSMA	Imaging	I/IIa	Luke Nordquist, MD	03/2022	n.s.	(NCT05286840)
[^64^Cu]Cu-Sar-bis-PSMA	Imaging	I/IIa	Clarity Pharmaceuticals Ltd	04/2022	Completed	COBRA (NCT05249127)
[^64^Cu]Cu-PSMA-I&T	Imaging	I/II	Curium US LLC	12/2022	Completed	(NCT05653856)
[^64^Cu]Cu-Sar-bis-PSMA	Imaging	III	Clarity Pharmaceuticals Ltd	12/2023	Recruiting	CLARIFY (NCT06056830)
[^64^Cu]Cu-PSMA-I&T	Imaging	III	Curium US LLC	04/2024	Active (not recruiting)	Solar-Recur (NCT06235099)
[^64^Cu]Cu-PSMA-I&T	Imaging	III	Curium US LLC	04/2024	Recruiting	Solar-Stage (NCT06235151)
[^61^Cu]Cu-NODAGA-PSMA	Imaging	I	Hoag Memorial Hospital Presbyterian	10/2024	Recruiting	(NCT06736054)
[^64^Cu]Cu-Sar-bis-PSMA [^68^Ga]Ga-PSMA-11	Imaging	II	St Vincent’s Hospital, Sydney	11/2024	Recruiting	Co-PSMA (NCT06907641)

Trial identifiers, sponsoring institutions, and names of the radioligands under investigation are also provided

### Gastrin-releasing peptide receptor

GRPR is a G protein-coupled receptor with seven transmembrane domains that binds gastrin-releasing peptide (GRP) in humans and bombesin (BBN) in animal models. GRPR and PSMA exhibit complementary expression profiles in PCa. While PSMA is overexpressed in high-grade, aggressive tumors, GRPR is typically upregulated in well-differentiated, androgen-sensitive, low-grade tumors [140]. This makes it a valuable target for non-invasive detection, staging, and treatment of PCa, particularly in cases with low or absent PSMA expression.

Several preclinical—and a few clinical—studies have investigated copper-based radiopharmaceuticals directed at GRPR (Tables 4 and 5), with copper-64 as the radionuclide of choice. The vast majority of these studies focus on GRPR agonists and antagonists derived from peptide-based BBN analogues, using either the full-length amphibian tetradecapeptide or truncated fragments such as BBN(7–14), which retain the receptor-binding bioactive region.

Similarly to what is observed with PSMA, DOTA chelators are widely used in GRPR-targeted copper-based radiopharmaceutical investigations. In an early study, Rogers et al. evaluated [^64^Cu]Cu-DOTA-Aoc-BBN(7–14), which enabled tumor visualization but exhibited high liver and intestinal uptake, as well as prolonged blood retention [141]. Chen et al. reported improved pharmacokinetics with [^64^Cu]Cu-DOTA-[Lys^3^]BBN, demonstrating rapid blood clearance and lower off-target uptake, with GRPR-expressing tumors being clearly visible. However, moderate liver uptake persisted over time [142]. In a subsequent study, Yang et al. compared this tracer to the truncated analogue [^64^Cu]Cu-DOTA-Aca-BBN(7–14), confirming superior tumor uptake and contrast with the full-length sequence, while the shorter version exhibited extremely poor metabolic stability. Liver accumulation remained a concern for both compounds [143]. Biddlecombe et al. evaluated the full-length BBN analogue MP2346 (DOTA-(Pro^1^,Tyr^4^)-BBN(1–14)) radiolabeled with copper-64 and yttrium-86. Both radioconjugates allowed tumor detection, but [^86^Y]Y-MP2346 showed higher image contrast due to lower uptake in clearance organs and reduced liver accumulation [144]. In an effort to mitigate this hepatic burden, Parry et al. replaced aliphatic linkers with amino acid sequences [145]. These modifications resulted in improved pharmacokinetics and reduced liver uptake compared to earlier constructs, while preserving tumor visualization [141, 143]. Nonetheless, the inherent limitations of DOTA chelation with copper remained a challenge.

**Fig. 14:**
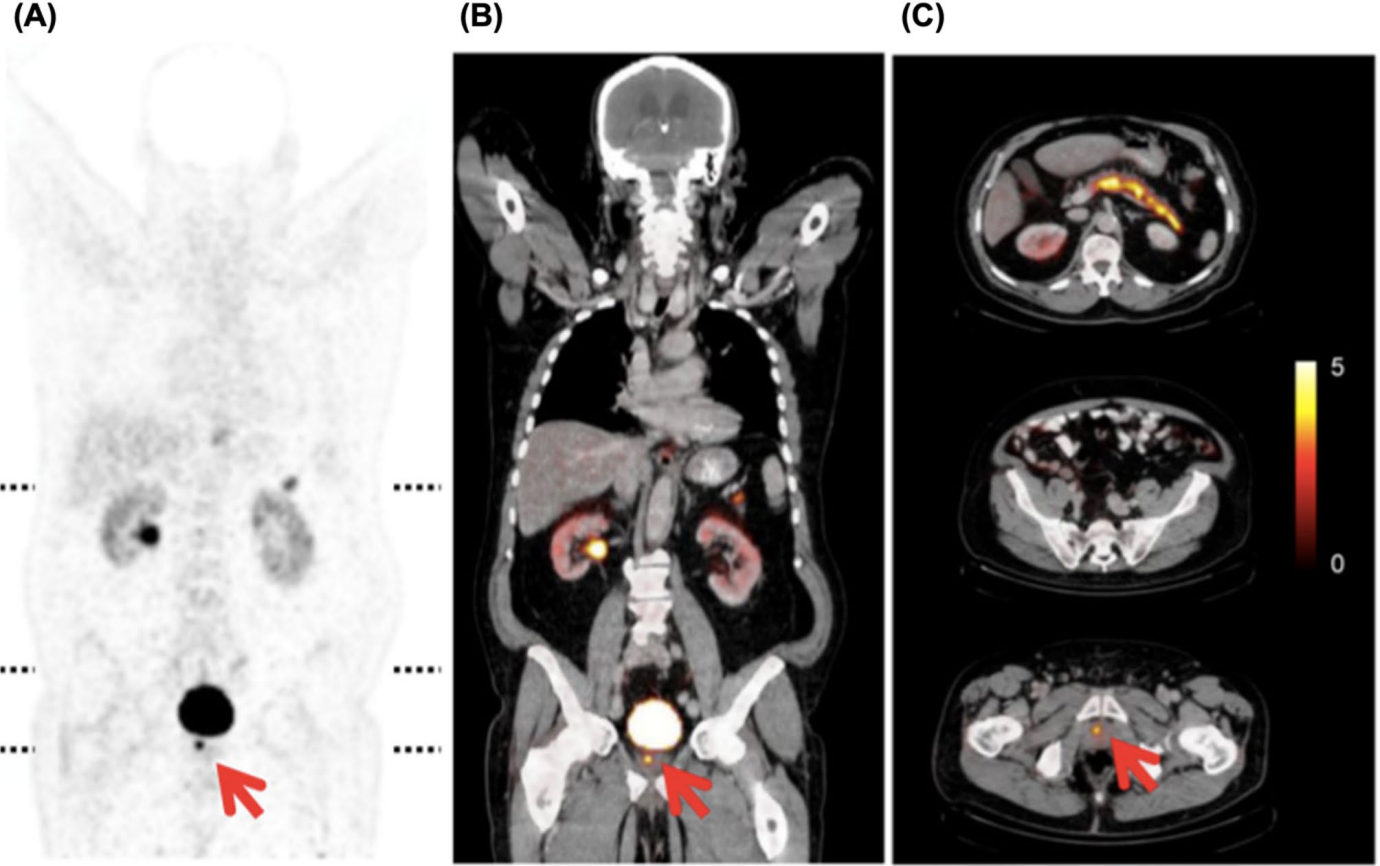
[^64^Cu]Cu-CB-TE2A-AR06 PET/CT images acquired 4 h post-injection. Coronal PET (**A**) and fused PET/CT (**B**) sections demonstrate intense tracer uptake in the prostate tumor (red arrows) and physiological uptake in the pancreas. Axial PET/CT fusion images (**C**) correspond to the levels indicated by the dotted lines in (**A**). Only low uptake is seen in the kidneys, liver, and intestines. PET images are scaled to a maximum SUV of 5 (color bar). Reproduced from Wieser et al. [148], licensed under CC BY-NC-ND 3.0 (https://creativecommons.org/licenses/by-nc-nd/3.0/)

To further address this concern, a noticeable shift toward the evaluation of alternative chelators is becoming evident. For example, Garrison et al. demonstrated that replacing DOTA with CB-TE2A in [^64^Cu]Cu-Aoc-BBN(7–14)NH_2_ significantly reduced liver and blood retention, thereby improving tumor-to-background contrast [146]. Similarly, Abiraj et al. investigated a CB-TE2A-based BBN-antagonist radioligand and reported high tumor uptake with excellent in vivo stability and favorable biodistribution [147]. These preclinical findings laid the groundwork for a first-in-human study by Wieser et al., who evaluated [^64^Cu]Cu-CB-TE2A-AR. Notably, the tracer demonstrated favorable dosimetry and high tumor contrast (Fig. 14) in three of four newly diagnosed PCa patients, with no adverse effects [148].

Another promising class of chelators which has shown improvements in the pharmacokinetics of GRPR-targeted copper-based radiopharmaceuticals includes NOTA and its derivatives. Prasanphanich et al. first demonstrated the feasibility of a [^64^Cu]Cu-NOTA-labeled BBN(7–14) analogue, which exhibited high receptor specificity, minimal liver accumulation, and fast renal clearance [149]. Based on these findings, Lane et al. synthesized a series of [^64^Cu]Cu-NO2A-(X)-BBN(7–14)NH_2_ conjugates with varied spacers to fine-tune pharmacokinetics and tumor retention [150]. Their investigations suggested that increased hydrophobicity often led to higher liver uptake, highlighting the importance of spacer design. Among the tested constructs, [^64^Cu]Cu-NO2A-AMBA-BBN(7–14)NH_2_ was the most promising candidate, combining high tumor uptake, minimal abdominal accumulation, and superior tumor-to-liver and tumor-to-kidney ratios relative to DOTA, CB-TE2A, and NOTA analogues [141, 146, 149]. Further refinement of NOTA chelation was explored by Craft et al., who designed a six-coordinate NOTA-BBN conjugate. While the radiopharmaceutical performed well at early imaging time points, the signal decreased significantly at later time points, denoting a trade-off between coordination geometry and tumor retention [151]. In parallel, Nanda et al. introduced a series of NO2A-based antagonists of the form [^64^Cu]Cu-NO2A-X-D-Phe-BBN(6–13)NHEt with increasing degrees of hydrophobicity. All variants showed high GRPR-specific tumor uptake, rapid blood clearance, and minimal liver accumulation, which correlated with the hydrophobicity of the X group [152]. Tumor retention at 4 h post-injection exceeded that of agonist analogs [150], reinforcing the value of antagonist frameworks. A follow-up study compared agonist ([^64^Cu]Cu-NODAGA-6-Ahx-BBN(7–14)NH_2_) and antagonist ([^64^Cu]Cu-NODAGA-6-Ahx-D-Phe-BBN(6–13)NHEt) variants. Interestingly, while the antagonist yielded higher early uptake and contrast, the agonist offered improved tumor-to-background ratios at later time points with minimal off-target accumulation and prolonged tumor retention [153]. These results underscore the importance of tracer selection based on intended imaging time points. Liu et al. compared NODAGA-RM1 and NODAGA-AMBA peptides radiolabeled with copper-64 or [^18^F]AlF, and observed superior tumor retention and lower kidney uptake for RM1 [154]. Baun et al. directly compared NOTA- and NODAGA-PEG2-RM26 antagonists radiolabeled with copper-64 or cobalt-55/57. Although the [^64^Cu]Cu-NOTA conjugate had higher liver uptake, it also showed superior tumor retention than its NODAGA counterpart. Despite slightly lower tumor-to-non-tumor ratios compared to cobalt analogs, copper-based radiopharmaceuticals still enabled clear tumor visualization [155]. These findings align with those from Gourni et al., who had previously reported improved pharmacokinetics and GRPR affinity for [^64^Cu]Cu-NOTA-MJ9 over [^64^Cu]Cu-NODAGA-MJ9 [156]. Makris et al. further validated this trend using NOTA and NODAGA conjugates of a statine-based antagonist. Both showed excellent GRPR-specific uptake and low off-target accumulation, but the NOTA derivative exhibited superior tumor retention at 24 h [157].

Studies evaluating linker effects have also provided valuable insights. Kim et al. incorporated a galactose moiety into [^64^Cu]Cu-NODAGA-BBN, resulting in improved tumor uptake, lower liver retention, and rapid renal excretion compared to the unmodified version [158]. In a separate study, Mansour et al. compared three NOTA-RM26-based antagonists with PEG, APCA, and AHDA linkers. All variants exhibited strong tumor uptake and fast washout from non-targeted organs, but the dicationic APCA conferred the most favorable hepatic clearance profile [159].

**Fig. 15:**
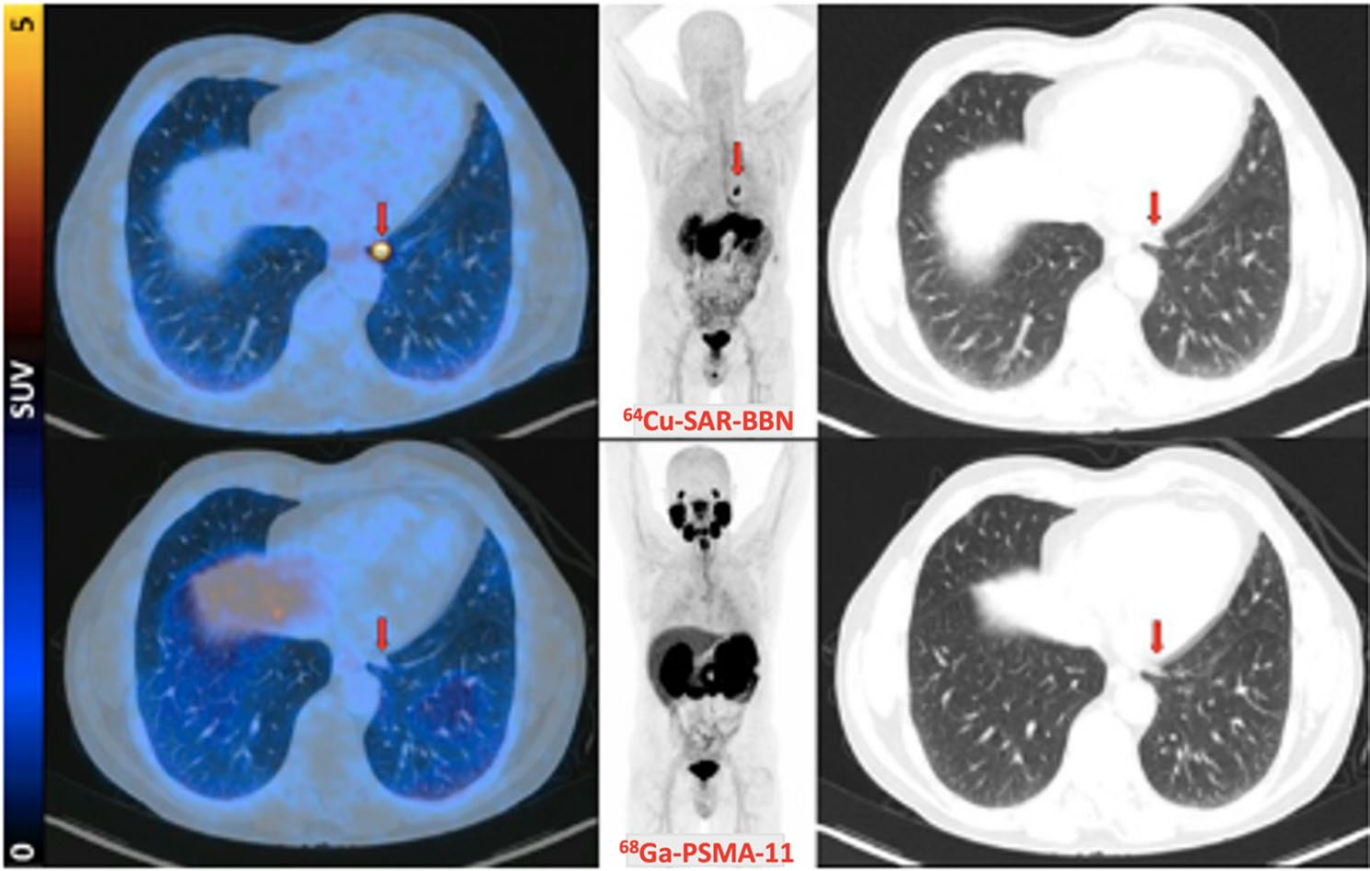
From left to right: fused PET/CT, maximum-intensity projection PET, and lung-windowed CT images acquired using [^64^Cu]Cu-SAR-BBN (top row) and [^68^Ga]Ga-PSMA-11 (bottom row). A left subpleural lesion (arrows) demonstrated uptake with [^64^Cu]Cu-SAR-BBN (SUV_max 10 at 1 h) but no uptake with [^68^Ga]Ga-PSMA-11. The patient had a PSA level of 1.84 ng/mL at the time of imaging and subsequently underwent lobectomy, with histopathology confirming metastatic PCa. Reproduced from Li et al. [140]

Lears et al. explored SarAr as a chelating strategy, conjugating it to BBN(7–14) via different linkers [160]. The radiotracers showed high GRPR-specific tumor uptake and faster blood clearance than DOTA-based analogs [145, 146]. However, liver accumulation remained significant, and SarAr-based conjugates did not offer clear advantages over CB-TE2A or NOTA-based compounds in terms of overall background reduction [146, 149]. Gourni et al. later incorporated MeCOSar into statine-based antagonists via PEG_2_ and PEG_4_ linkers, which resulted in low off-target accumulation and high tumor retention, particularly for the PEG_4_ variant [161]. These favorable properties prompted its clinical translation for PET imaging of GRPR-expressing malignancies, including PCa. Li et al. reported on the BOP clinical trial (NCT05613842), where [^64^Cu]Cu-SAR-BBN PET/CT detected lesions in 44% of patients with BCR and negative or equivocal PSMA PET (Fig. 15), influencing clinical management in 73% of cases [140]. Further validation is underway in the SABRE clinical trial (NCT05407311), a Phase II study in PSMA-negative BCR patients, which has already completed recruitment [162]. Building on these results, Huynh et al. investigated [^67^Cu]Cu-SAR-BBN in a preclinical model, showing significant tumor growth inhibition and survival benefit [163]. The COMBAT clinical trial (NCT05633160) is currently evaluating [^67^Cu]Cu-SAR-BBN for radioligand therapy in metastatic castration-resistant PCa patients ineligible for [^177^Lu]Lu-PSMA-617 due to low PSMA expression [164], offering a promising alternative for this underserved patient subset.

Different chelation strategies have also been investigated. Juran et al. evaluated bispidine-based chelators but found their BBN-constructs suffered from rapid tumor washout and high non-targeted accumulation, deeming them unsuitable for further development [165]. In contrast, Bergmann et al. assessed DMPTACN-conjugated agonists and reported high tumor uptake and favorable clearance profiles, with an extra glutamic acid spacer providing improved tumor-to-muscle ratios [166]. The use of the novel DOTHA_2_ chelator was explored by Mansour et al., who evaluated the GRPR antagonist [^64^Cu]Cu-DOTHA_2_-PEG-RM26 [167]. This tracer showed strong tumor accumulation, low uptake in non-targeted tissues, and excellent image contrast, outperforming its NOTA-based analogue in terms of sustained tumor retention and overall image quality [159]. On the other hand, Cai et al. pursued a nanoplatform approach with BBN-PEG-[^64^Cu]CuS nanoparticles for dual-mode imaging. While these constructs enabled clear orthotopic tumor visualization, prominent hepatic accumulation was observed, likely due to nanoparticle size and excretion pathways [168].

The optimization of GRPR-targeted radiopharmaceuticals has extended to the development of multimeric constructs, offering an alternative strategy to enhance receptor binding and tumor accumulation. Fournier et al. compared [^64^Cu]Cu-NOTA-labeled monomeric and dimeric BBN analogs. While the monomer exhibited faster renal clearance and better early contrast, the dimer provided longer and more sustained tumor uptake [169]. Bandari et al. expanded this concept by designing a bivalent vector incorporating both GRPR/PSMA-targeting motifs, demonstrating effective tumor uptake but also liver and gastrointestinal accumulation, highlighting the need for further refinement [170].

Innovative pharmacological strategies have also been explored to enhance image contrast. Matsumura et al. used a urokinase-cleavable linker in a PEGylated tetrameric BBN-construct, enabling triggered clearance of circulating radioconjugate without compromising tumor uptake [171]. This approach improved tumor-to-blood ratios and reduced background signal, illustrating a novel avenue for optimizing GRPR-targeted imaging.

Together, these studies reflect the multifaceted efforts to optimize GRPR-targeted copper-based radiopharmaceuticals. As these agents advance into clinical trials, including theranostic applications, they present promising prospects for improved management of GRPR-expressing PCa.

**Table 4:** Overview of published studies on copper-based radiopharmaceuticals directed at GRPR for PCa imaging and therapy in both preclinical and clinical contexts, arranged in chronological order

Radionuclide(s)	Chelator(s)	Targeting agent(s)	Target(s)	Study design	Cell line(s)	Mouse strain	Reference(s)	Year of publication
^64^Cu	DOTA	8-Aoc-BBN(7–14)NH_2_	GRPR	Preclinical	PC-3	♀ athymic nude	Rogers	2003
^64^Cu	DOTA	[Lys^3^]BBN	GRPR	Preclinical	PC-3 and CWR22	♂ athymic nude	Chen	2004
^64^Cu	DOTA	Aca-BBN(7–14) and [Lys^3^]BBN	GRPR	Preclinical	PC-3 and 22Rv1	♂ athymic *nu/nu*	Yang	2006
^64^Cu and ^86^Y	DOTA	MP2346	GRPR	Preclinical	PC-3	♂ athymic nude	Biddlecombe	2007
^64^Cu	CB-TE2A and DOTA	8-Aoc-BBN(7–14)NH_2_	GRPR	Preclinical	PC-3	♀ ICR SCID	Garrison	2007
^64^Cu	DOTA	BBN(7–14)NH_2_	GRPR	Preclinical	PC-3	♀ SCID	Parry	2007
^64^Cu	NOTA	8-Aoc-BBN(7–14)NH_2_	GRPR	Preclinical	PC-3	♀ ICR SCID	Prasanphanich	2007
^64^Cu	(3,7-diazabicyclo [3.3.1]nonane)-based	β*homo*-Glu- βAla-βAla-[Cha^13^,Nle^14^] BBN(7–14)	GRPR	Preclinical	PC-3	NMRI nu/nu	Juran	2009
^64^Cu	NO2A	(X)-BBN(7–14)NH_2_	GRPR	Preclinical	PC-3	♀ ICR SCID	Lane	2010
^111^In, ^99m^Tc, ^68^Ga, and ^64^Cu	DOTA, N4, NODAGA, and CB-TE2A	PEG_4_-D-Phe-BBN(7–12)-Sta-Leu-NH_2_	GRPR	Preclinical	PC-3	♀ nude	Abiraj	2011
^64^Cu	SarAr	SA-Aoc-BBN(7–14) and SA-Aoc-GSG-BBN(7–14)	GRPR	Preclinical	PC-3	♀ CB.17 SCID	Lears	2011
^64^Cu	NOTA	8-Aoc- BBN(7–14)	GRPR	Preclinical	PC-3	♀ SCID	Craft	2012
^64^Cu	NOTA	[D-Tyr^6^,βAla^11^, Thi^13^,Nle^14^] BBN(6–14)	GRPR	Preclinical	PC-3	♀ Balb/c nude	Fournier	2012
^64^Cu	NODAGA	6-Ahx-BBN(7–14)NH_2_ and 6-Ahx-*D*Phe^6^-BBN(6–13)NHEt	GRPR	Preclinical	PC-3	♀ ICR SCID	Nanda	2012
^64^Cu	NO2A	X-*D*Phe^6^-BBN(6–13)NHEt	GRPR	Preclinical	PC-3	♀ ICR SCID	Nanda	2012
^64^Cu	DMPTACN	βAla-βAla-[Cha^13^,Nle^14^] BBN(7–14) and β*homo*-Glu- βAla-βAla-[Cha^13^,Nle^14^] BBN(7–14)	GRPR	Preclinical	PC-3	♀ NMRI *nu/nu*	Bergmann	2013
^64^Cu and ^18^F-AlF	NODAGA	RM1 and AMBA	GRPR	Preclinical	PC-3	♂ nude	Liu	2013
^64^Cu	PPIX and DOTA	PEG_6_-BBN	GRPR	Preclinical	PC-3	♀ BALB/c nu/nu	Mukai	2013
^64^Cu	NODAGA	DUPA and BBN(7–14)NH_2_	PSMA and GRPR	Preclinical	LNCaP and PC-3	♂ athymic nude-*Fox1*^nu^ and ♀ ICR SCID	Bandari	2014
^68^Ga and ^64^Cu	NOTA and NODAGA	MJ9	GRPR	Preclinical	PC-3	♀ athymic BALB/C nude	Gourni	2014
^64^Cu	CB-TE2A	PEG_4_-D-Phe-BBN(7–12)-Sta-Leu-NH_2_	GRPR	Clinical	–	–	Wieser	2014
^64^Cu	MeCOSar	D-Phe-BBN(7–12)-Sta-Leu-NH_2_	GRPR	Preclinical	PC-3	♀ athymic nude	Gourni	2015
^64^Cu	NODAGA	BBN(7–14)NH_2_ and Galacto-BBN(7–14)NH_2_	GRPR	Preclinical	PC-3	♂ athymic Nu/Nu BALB/c	Kim	2015
^64^Cu	NOTA	(LK)-RM26	GRPR	Preclinical	PC-3	♀/♂ athymic nu/nu	Mansour	2017
[^64^Cu]CuS NPs	–	Lipoamide-dPEG_12_-BBN	GRPR	Preclinical	PC-3-KD1	♂ Nu/Nu	Cai	2018
^64^Cu	DOTHA_2_	PEG-RM26	GRPR	Preclinical	PC-3	♂ athymic nu/nu	Mansour	2018
^64^Cu	NODAGA	4arm-PEG-(USL-BBN)_4_	GRPR	Preclinical	PC-3	♂ BALB/c nu/nu	Matsumura	2018
^64^Cu and ^55^Co	NOTA and NODAGA	RM26	GRPR	Preclinical	PC-3	♂ NOD-scid	Baun	2020
^64^Cu	NOTA and NODAGA	D-Phe-BBN(7–12)-Sta-Leu-NH_2_	GRPR	Preclinical	PC-3	♂ ICR SCID	Makris	2021
^67^Cu	MeCOSar	BBN	GRPR	Preclinical	PC-3	Athymic nude	Huynh	2022
^64^Cu	Sar	BBN	GRPR	Clinical	–	–	Li	2024

The coding differentiates between peptides and peptidomimetic small molecules

**Table 5:** Summary of completed and ongoing clinical trials evaluating GRPR-targeted, copper-based radiopharmaceuticals for PCa imaging and therapy

Radiopharmaceutical	Intervention	Phase	Sponsor	Starting date	Status	Name (ID)
[^64^Cu]Cu-Sar-BBN	Imaging	IIa	St Vincent’s Hospital, Sydney	08/2022	Completed	BOP (NCT05613842)
[^64^Cu]Cu-Sar-BBN	Imaging	IIa	Clarity Pharmaceuticals Ltd	09/2022	Completed	SABRE (NCT05407311)
[^64^Cu]Cu-Sar-BBN [^67^Cu]Cu-Sar-BBN	Imaging/Therapy	I/IIa	Clarity Pharmaceuticals Ltd	06/2023	Recruiting	COMBAT (NCT05633160)

Studies are listed by starting date and include trial identifiers, sponsoring entities, and the name of the agents under investigation

### Other targets

In addition to PSMA and GRPR, numerous alternative molecular targets have been explored for PCa imaging and therapy using ^64^Cu-labeled radiopharmaceuticals, each offering unique biological rationales and translational potential (Tables 6 and 7).

Integrins, particularly α_v_β_3_ and α_2_β_1_, are widely expressed in tumors and play key roles in angiogenesis and metastasis, making them attractive targets for molecular imaging. Cai et al. used [^64^Cu]Cu-DOTA-Abegrin™, a mAb specific to integrin α_v_β_3_, achieving good tumor delineation, albeit with hepatic clearance due to its large size [172]. Several radioconjugates have since been developed to target integrin α_v_β_3_ using RGD peptides. Liu et al. pioneered the use of ^64^Cu-labeled RGD-BBN heterodimers for dual-receptor targeting of integrin α_v_β_3_ and GRPR, demonstrating improved tumor uptake over single-receptor monomers or their mixtures [173]. In a subsequent study, Jackson et al. further refined the chelator chemistry of the RGD-BBN scaffold using NO2A, aiming to reduce renal retention and optimize pharmacokinetics by modulating the overall charge of the complex [174]. Their comparison of neutral versus anionic [173] constructs highlighted the influence of chelator conjugation strategy on kidney uptake and overall image contrast. Building on this concept, Durkan et al. designed a structurally distinct heterodimeric radiopharmaceutical with a BBN antagonist motif, [^64^Cu]Cu-NO2A-RGD-RM2, achieving high and prolonged tumor retention with minimal abdominal background signal [175]. Later, Lucente et al. investigated the impact of a long polyglycine linker between the RGD and BBN targeting moieties, aiming to improve their spatial arrangement and binding efficiency. However, tumor uptake remained low, probably due to poor in vivo pharmacokinetics [176]. Yuan et al. used a polymeric RGD-conjugated scaffold (polyHPMA) for integrin α_v_β_3_-positive tumor localization and drug delivery [177]. On the other hand, Jadvar et al. evaluated a ^64^Cu-labeled disintegrin, vicrostatin (VCN), for targeting integrins in PCa xenografts. While [^64^Cu]Cu-Sar-RGD demonstrated slightly higher tumor uptake, the [^64^Cu]Cu-Sar-PEG-VCN radioconjugate provided superior tumor-to-background contrast with reduced off-target abdominal accumulation [178]. Hu et al. explored the α_2_β_1_ integrin with a DGEA-functionalized GdVO_4_ nanosheet for multimodal imaging, including microPET [179].

The Eph family of receptor tyrosine kinases is also implicated in tumor progression. EphB4 was targeted by Xiong et al. using the TNYL-RAW peptide radiolabeled with [^64^Cu]Cu-DOTA, yielding specific tumor uptake in EphB4-positive models [180]. For imaging EphA2, which is overexpressed in various cancers, Ullman et al. [181] applied [^64^Cu]Cu-MeCOSar-labeled Abdurins, novel antibody-like scaffolds, while Pyo et al. [182] used the [^64^Cu]Cu-NOTA-labeled monobody E1. Both radioconjugates showed specific tumor accumulation and exhibited favorable pharmacokinetics.

**Fig. 16:**
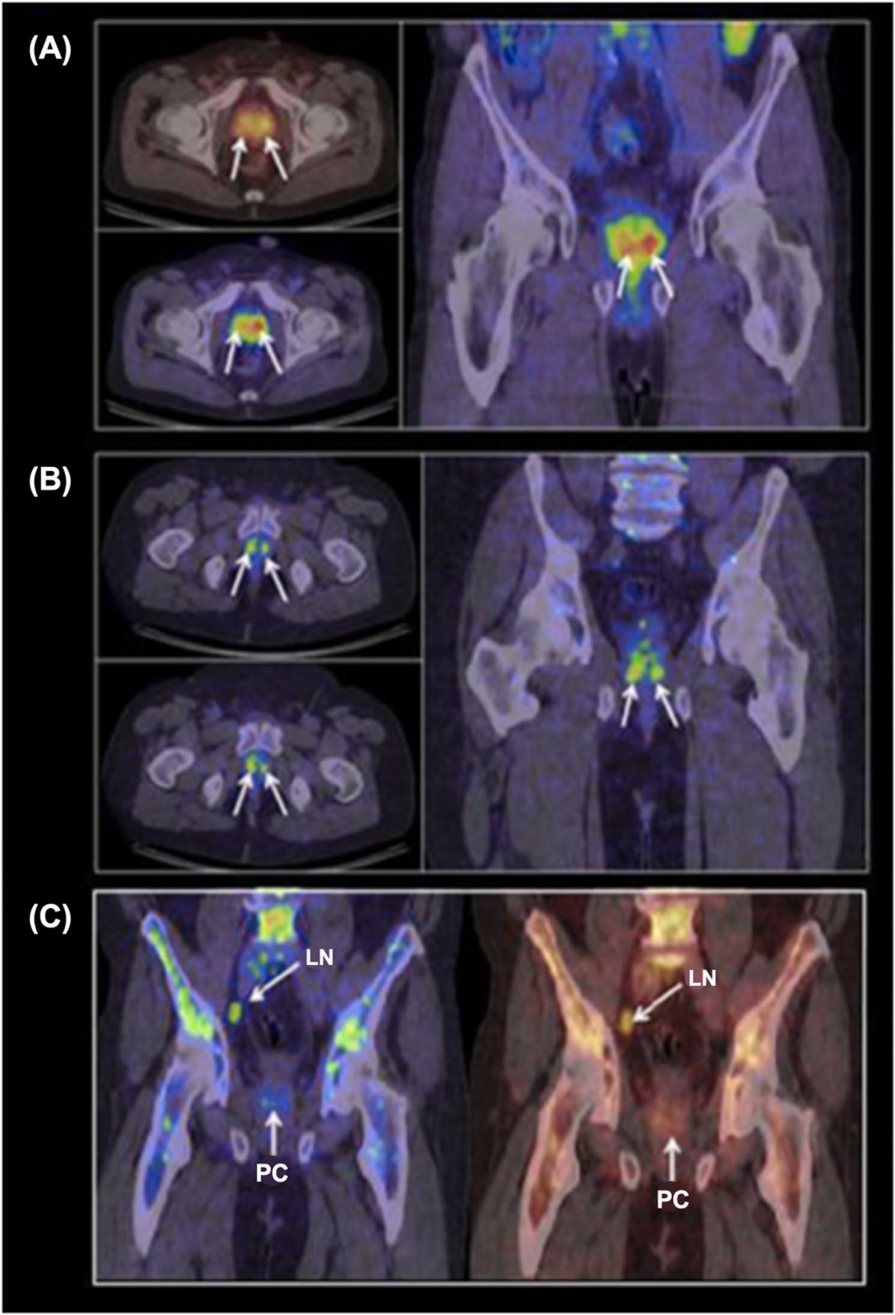
Cross-sectional and coronal PET/CT images acquired 30 min after injection of [^64^Cu]Cu-TP3805 in two patients with PCa (**A** and **B**) show multiple intraprostatic lesions (arrows), and minimal bladder and bone marrow uptake. In a third patient (**C**), PET/CT images show a primary lesion and a lymph node with marked tracer uptake (arrows), later confirmed as malignant by histology, along with more prominent bone marrow activity. [^99m^Tc]Tc-MDP bone scans were negative in all cases. Reproduced with permission from Tripathi et al. [184]

VPAC1, the receptor for vasoactive intestinal peptide (VIP), is overexpressed in PCa and contributes to tumor proliferation and progression. Zhang et al. [183] and Tripathi et al. [184] developed [^64^Cu]Cu-DDT-labeled VIP analogs—TP3939 and TP3805, respectively—to selectively bind to VPAC1-expressing cells. The work by Tripathi et al. advanced to clinical evaluation, demonstrating high specificity in identifying malignant prostate lesions (Fig. 16). Similarly, neurotensin receptor 1 (NTR-1), activated in androgen-independent PCa, was effectively targeted by Deng et al. with three ^64^Cu-labeled NT analogs, all achieving good tumor contrast in imaging studies [185].

Vascular endothelial growth factor receptor 2 (VEGFR2), a critical regulator of angiogenesis, was targeted by Hao et al. using [^64^Cu]Cu-DOTA-labeled GU40C4, a peptoid antagonist. PET imaging showed specific accumulation in VEGFR2-positive tumors [186]. Pressly et al. targeted another angiogenic marker, the natriuretic peptide clearance receptor (NPRC), with a [^64^Cu]Cu-CANF-Comb nanoparticle which demonstrated high tumor uptake and minimal hepatic retention [187], underscoring its specificity and favorable pharmacokinetics.

EpCAM, a cell adhesion molecule overexpressed in prostate and other epithelial cancers, was visualized by Hall et al. using dual-labeled antibodies (mAb-7 and mAb-153) conjugated with [^64^Cu]Cu-DOTA and IRDye800. These agents enabled PET and near-infrared fluorescence imaging, accurately detecting metastatic lymph nodes and outperforming commercial antibodies [188].

The protease PACE4, involved in proprotein processing and tumor progression, was targeted by Couture et al. using a multi-leucine peptide inhibitor radiolabeled with [^64^Cu]Cu-NOTA, which showed specific accumulation in PACE4-positive tumors and rapid renal clearance, allowing clear tumor visualization [189]. Park et al. targeted hepsin, another PCa-specific protease, with ^64^Cu-labeled Leu-Arg dipeptide derivatives, achieving successful imaging in hepsin-positive models [190].

Extradomain-B fibronectin (EDB-FN), commonly enriched in aggressive tumors, was targeted by Han et al. with the ZD2 peptide conjugated to [^64^Cu]Cu-DOTA. This probe enabled stratification of tumor aggressiveness, although moderate DOTA stability led to some hepatic uptake [191]. Another extracellular matrix protein, the receptor for advanced glycation end-products (RAGE), was visualized by Konopka et al. using dendrimer-based nanoparticles. [^64^Cu]Cu-Cy5-G4-CML exhibited tumor-specific uptake which correlated with Gleason scores [192], suggesting potential for disease grading.

Kumar et al. introduced a modular click-chemistry approach for labeling antibodies with copper-64 using CB-TE2A, enabling efficient radiolabeling of anti-PSMA and anti-phosphatidylserine antibodies. PET imaging with ^64^Cu-labeled bavituximab confirmed specific tumor accumulation and prolonged retention, illustrating the feasibility and versatility of this strategy for developing stable, targeted antibody-based radioconjugates [193]. Prostate stem cell antigen (PSCA), another surface marker overexpressed in PCa, has also been targeted by multiple radiopharmaceuticals. David et al. used a ^64^Cu-labeled anti-PSCA antibody with bis(phosphinate) cyclam chelators, achieving high tumor uptake and low off-target activity [194]. Arndt et al. developed a bispecific antibody (IgG4-TM) for both imaging and therapy, radiolabeled with copper-64 and actinium-225, respectively [195]. Kubeil et al. extended this work by using picolinate-based bispidine chelators (L1) in PSCA- and fibroblast activation protein (FAP)-targeted TMs, demonstrating specific tumor accumulation and prolonged circulation [196].

The follicle-stimulating hormone receptor (FSHR), selectively overexpressed in tumor vasculature, was targeted by Hong et al. with [^64^Cu]Cu-NOTA-labeled mAbs. Uptake in various tumors, including prostate tumors, correlated with FSHR expression, highlighting its angiogenic relevance [197]. Additionally, the growth hormone secretagogue receptor 1a (GHS-R1a), part of the ghrelin signaling axis, was addressed by Bergmann et al. using [^64^Cu]Cu-NODAGA-labeled inverse agonist peptides, successfully delineating PC-3 and DU145 xenografts [198].

**Fig. 17:**
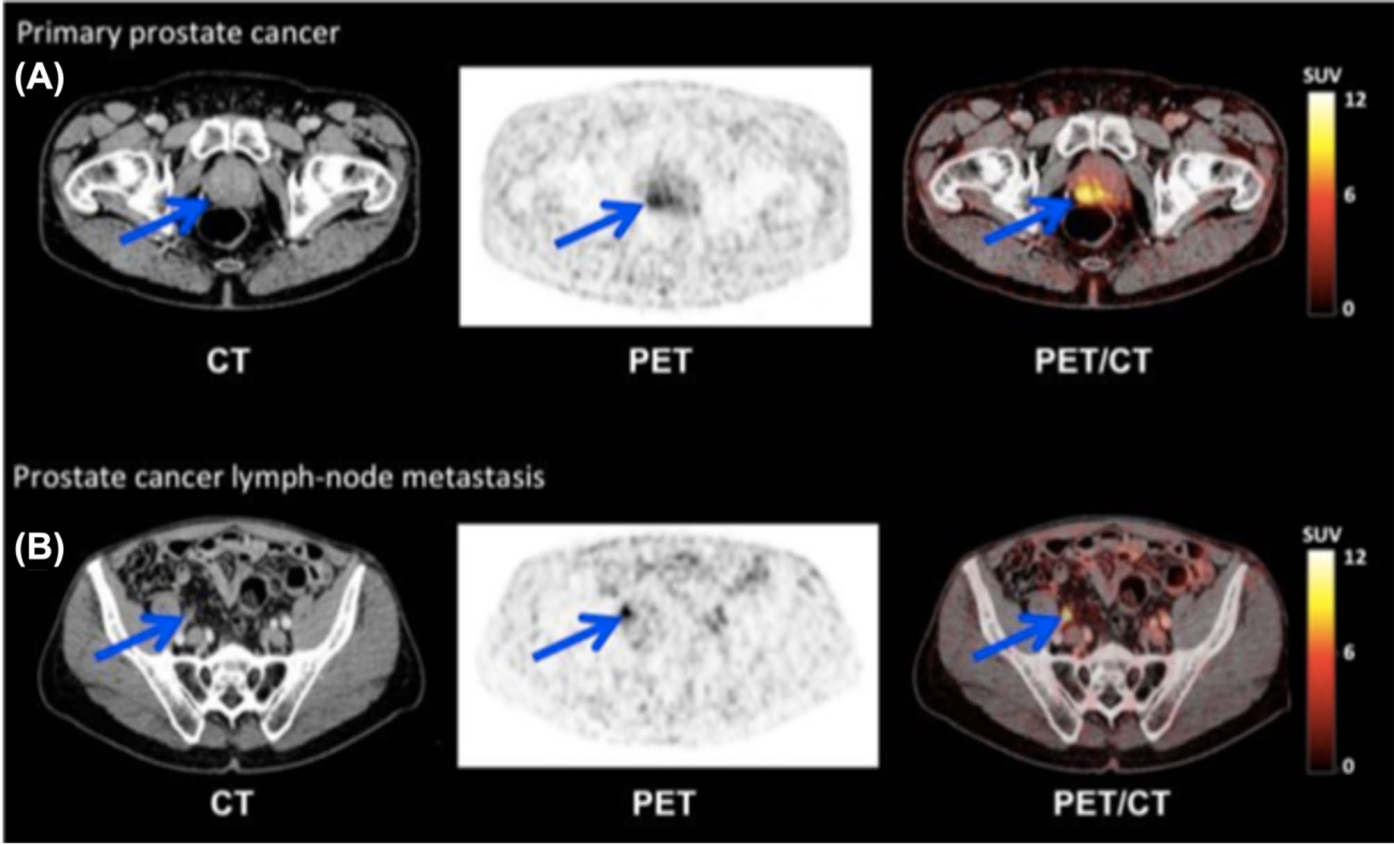
uPAR PET imaging in PCa. (**A**) Representative transverse CT, PET, and fused PET/CT images of a primary tumor lesion (blue arrows) showing high uptake of [^64^Cu]Cu-DOTA-AE105. (**B**) Images from another case depict a uPAR-positive regional lymph node metastasis (blue arrows) with similarly high tracer accumulation. Reproduced from Persson et al. [199], licensed under CC BY-NC-ND 3.0 (https://creativecommons.org/licenses/by-nc-nd/3.0/)

Clinical studies further illustrate the translational potential of these agents. A first-in-human study by Persson et al. [199] with uPAR-targeted [^64^Cu]Cu-DOTA-AE105 demonstrated its safety, with satisfactory in vivo stability and effective imaging of both primary tumors and lymph node metastases (Fig. 17). A follow-up trial (NCT06474806) is currently evaluating AE105 in low-risk PCa patients under active surveillance, aiming to support non-invasive monitoring [200]. Another innovative clinical trial (NCT05888532) is investigating [^64^Cu]Cu-GRIP-B, a granzyme B-targeted agent for imaging immune activation in genitourinary malignancies, including PCa.

In summary, the landscape of ^64^Cu-labeled radiopharmaceuticals for PCa has significantly expanded beyond traditional molecular targets. These advances underscore the versatility of molecular imaging and TRT in addressing disease heterogeneity. Emerging agents offer promising opportunities for improved disease characterization, patient stratification, and individualized treatment planning. As integration into clinical workflows steadily progresses, ongoing research and trials remain key to refining their safety, efficacy, and clinical impact—driving forward the evolution of precision oncology in PCa care.

**Table 6:** Overview of published preclinical and clinical studies reporting on distinct copper-based radiopharmaceuticals for targeting various molecular targets, aside from PSMA and GRPR, in PCa

Radionuclide(s)	Chelator(s)	Targeting agent(s)	Target(s)	Study design	Cell line(s)	Mouse strain	Reference(s)	Year of publication
^64^Cu	DOTA	Abegrin™	Integrin α_v_β_3_	Preclinical	U87MG, PC-3, MDA-MB-435, and GL-26	♀/♂ athymic nude	Cai	2006
^64^Cu	DOTA	Poly[AMPA]	–	Preclinical (intratumoral injection)	PC-3	♂ athymic	Yuan	2007
^64^Cu	DDT	TP3939	VPAC1	Preclinical	PC-3	♂ athymic nude	Zhang	2008
^64^Cu	DOTA and NOTA	RGD, BBN(7–14), and RGD-BBN(7–14)	Integrin α_v_β_3_ and GRPR	Preclinical	PC-3	♂ athymic nude	Liu	2009
^64^Cu	DOTA	GU40C4	VEGFR2	Preclinical	PC-3	SCID	Hao	2011
^64^Cu	DOTA	TNYL-RAW	EphB4	Preclinical	CT26, PC-3 M, and A549	♀/♂ athymic nude	Xiong	2011
^64^Cu	DOTA	MAB9601-IRDye800	EpCAM	Preclinical	PC-3	♂ Nu/Nu	Hall	2012
^64^Cu	DOTA	mAb-7-IRDye800 and mAb-153-IRDye800	EpCAM	Preclinical	PC-3	♂ nu/nu	Hall	2012
^64^Cu	NO2A	RGD-BBN(7–14)NH_2_	Integrin α_v_β_3_ and GRPR	Preclinical	PC-3	♀ CF-1 and SCID	Jackson	2012
^64^Cu	–	Porphyrin-NPs	–	Preclinical	PC-3	♂ athymic nude	Liu	2012
^64^Cu	DOTA	CANF-Comb	NPRC	Preclinical	CWR22	♂ athymic nu/nu	Pressly	2013
^64^Cu	DOTA	(RGDyK)c-poly(HPMA)	Integrin α_v_β_3_	Preclinical	PC-3	Athymic	Yuan	2013
^64^Cu	NOTA	Multi-leucine	PACE4	Preclinical	LNCaP and PC-3	♂ athymic nude	Couture	2014
^64^Cu	NO2A	RGD-RM2	Integrin α_v_β_3_ and GRPR	Preclinical	PC-3	♀ SCID	Durkan	2014
^64^Cu	DOTA	GdVO_4_:4%Eu-DGEA	Integrin α_2_β_1_	Preclinical	PC-3	♂ athymic BALB/c nu/nu	Hu	2014
^64^Cu	–	AuNCs-PEG350 and AuNCs-PEG1000	–	Preclinical	PC-3	♂ athymic nu/nu	Zhao	2014
^64^Cu	NOTA	FSHR-mAb	FSHR	Preclinical	CAOV-3, SKOV-3, MDA-MB-231, and PC-3	♂ nude	Hong	2015
^64^Cu	CB-TE2A-1 C	YPSMA-1 and Bavituximab	PSMA and Phospha- tidylserine	Preclinical	LNCaP	♂ SCID/Nude	Kumar	2015
^64^Cu	DOTA	AE105	uPAR	Clinical	–	–	Persson	2015
^64^Cu	MeCOSar	B6 and B11	EphA2	Preclinical	PC-3	♂ NUDE	Ullman	2015
^64^Cu	–	Porphyrin-NPs	–	Preclinical	PC-3 M-luc-C6	♂ athymic nude	Jin	2016
^64^Cu	DDT	TP3805	VPAC1	Clinical	–	–	Tripathi	2016
^64^Cu	DOTA, NOTA, and AmBaSar	NT20.3	NTR-1	Preclinical	HT-29, PC-3, and LNCaP	♀/♂ BALB/c nude, and NSG	Deng	2017
^64^Cu	L^5^ and NODAGA	7F5	PSCA	Preclinical	PC-3-PSCA	♂ nude	David	2018
^64^Cu	DOTA and NODAGA	RGD_2_-BBN(7–14)	Integrin α_v_β_3_ and GRPR	Preclinical	PC-3	♂ nude	Lucente	2018
^64^Cu	DOTA	ZD2	EDB-FN	Preclinical	LNCaP and PC-3	♂ athymic nude	Han	2019
^64^Cu	DiAmSar	PEG-VCN and RGD	Integrins	Preclinical	PC-3	♂ athymic nude	Jadvar	2019
^64^Cu	NOTA	Cy5-G4-CML	RAGE	Preclinical	LNCaP and DU145	NU/J	Konopka	2020
^64^Cu	NOTA	E1	EphA2	Preclinical	PC-3	nude	Pyo	2020
^64^Cu	NODAGA	KKD	GHS-R1a	Preclinical	PC-3 and DU145	♂ NMRI nu/nu	Bergmann	2021
^64^Cu and ^225^Ac	DOTAGA	IgG4-TM	PSCA	Preclinical	PC-3-PSCA/PSMA	♂ Rj: NMRI-Foxn1^nu/nu^	Arndt	2022
^64^Cu	DOTA	Leu-Arg	Hepsin	Preclinical	PC-3 and 22Rv1	♂ BALB/c nude	Park	2022
^64^Cu	L1	IgG4-TM	PSCA	Preclinical	PC-3, PC-3-PSCA/PSMA, HT1080, and HT1080 hFAP	♀ NMRI nude	Kubeil	2024

The coding is used to distinguish between different classes of targeting agents, including mAbs, monobodies, peptides, peptidomimetic small molecules, nanoparticles, polymers, luminescent and paramagnetic nanomaterials, nanoclusters, and engineered/native proteins

**Table 7:** Summary of completed and ongoing clinical trials investigating copper-based radiopharmaceuticals directed at alternative PCa molecular targets, namely, VPAC1, uPAR, and granzyme B. Studies are organized by initiation date and the table includes trial identifiers, sponsoring organizations, and the agents evaluated

Radiopharmaceutical	Target	Intervention	Phase	Sponsor	Starting date	Status	Name (ID)
[^64^Cu]Cu-TP3805	VPAC1	Imaging	I	Sydney Kimmel Cancer Center	08/2013	Completed	(NCT02603965)
[^64^Cu]Cu-DOTA-AE105	uPAR	Imaging	I	Rigshospitalet	05/2014	Completed	(NCT02139371)
[^64^Cu]Cu-TP3805	VPAC1	Imaging	I	Sydney Kimmel Cancer Center	09/2015	Completed	(NCT02989623)
[^64^Cu]Cu-GRIP-B	Granzyme B	Imaging	I/II	Rahul Aggarwal	05/2023	Recruiting	(NCT05888532)
[^64^Cu]Cu-DOTA-AE105	uPAR	Imaging	II	Curasight	06/2024	Recruiting	uTRACE-101 (NCT06474806)

## Conclusions

Copper radionuclides have emerged as versatile and powerful tools in the evolving field of PCa theranostics, offering unique advantages for both molecular imaging and TRT. Their favorable physical properties, diverse decay profiles, and compatibility with a broad range of chelators have enabled the development of a growing array of radiopharmaceuticals targeting key molecular features of PCa. Among these, PSMA and GRPR have been the most extensively explored, yielding promising clinical and preclinical results. At the same time, expanding interest in alternative molecular targets is broadening the landscape of potential applications and addressing critical gaps posed by disease heterogeneity.

Advancements in chelation chemistry, radiopharmaceutical design, and imaging protocols have collectively strengthened the translational potential of copper-labeled agents. From first-in-human studies to advanced-phase clinical trials, these compounds are increasingly demonstrating their value in improving patient stratification, guiding therapeutic decisions, and enabling more individualized care. As such, copper radionuclides are no longer experimental curiosities but are gradually becoming integral components of the modern oncologic toolkit. Nevertheless, the broader clinical translation of copper-based radiopharmaceuticals remains partially constrained by the limited availability of copper-67, owing to its complex production routes and current supply challenges. Addressing these logistical hurdles will be important to fully harness the theranostic potential of copper radionuclides. On the other hand, the FDA approval of [^64^Cu]Cu-DOTATATE has further strengthened interest in the development of novel and effective copper-based agents for diagnostic, therapeutic, and theranostic applications in oncology, including in the context of PCa.

Taken together, these advances underscore the ever-growing medical relevance of copper-based radiopharmaceuticals and their capacity to reshape the management of PCa. By consolidating current knowledge, highlighting ongoing challenges, and outlining future directions, this review aims to support continued research and foster the integration of copper-labeled agents into standard clinical practice. As the field moves forward, these tools are well positioned to contribute meaningfully to the next generation of personalized cancer care.

## Data Availability

Not applicable.

## Abbreviations

ADNG: Accelerator-driven neutron generators
ADT: Androgen deprivation therapy
BBN: Bombesin
BCR: Biochemical recurrence
CT: Computed tomography
FAP: Fibroblast activation protein
FDA: U.S. Food and Drug Administration
FSHR: Follicle-stimulating hormone receptor
GRP: Gastrin-releasing peptide
GRPR: Gastrin-releasing peptide receptor
LINAC: Linear accelerator
mAb: Monoclonal antibody
MRI: Magnetic resonance imaging
PCa: Prostate cancer
PET: Positron emission tomography
PSA: Prostate-specific antigen
PSCA: Prostate stem cell antigen
PSMA: Prostate-specific membrane antigen
Sar/SAR: Sarcophagine
SPECT: Single-photon emission computed tomography
TM: Target module
TME: Tumor microenvironment
TRT: Targeted radionuclide therapy
VCN: Vicrostatin
VEGFR: Vascular endothelial growth factor receptor

## References

[1] Bray F, Laversanne M, Sung H, Ferlay J, Siegel RL, Soerjomataram I, et al. Global cancer statistics 2022: GLOBOCAN estimates of incidence and mortality worldwide for 36 cancers in 185 countries. CA Cancer J Clin. 2024;74(3):229–63. 38572751 10.3322/caac.21834

[2] Sharma S, Zapatero-Rodríguez J, O’Kennedy R. Prostate cancer diagnostics: clinical challenges and the ongoing need for disruptive and effective diagnostic tools. Biotechnol Adv. 2017;35(2):135–49. 27939303 10.1016/j.biotechadv.2016.11.009

[3] Dushimova Z, Iztleuov Y, Chingayeva G, Shepetov A, Mustapayeva N, Shatkovskaya O, et al. Overdiagnosis and overtreatment in prostate cancer. Diseases. 2025;13(6):167. 10.3390/diseases13060167 40558578 10.3390/diseases13060167 PMC12191725

[4] Kania E, Janica M, Nesterowicz M, Modzelewski W, Cybulski M, Janica J. Advances and challenges in prostate cancer diagnosis: a comprehensive review. Cancers (Basel). 2025;17:13. 10.3390/cancers17132137 PMC12248846 40647437

[5] Manafi-Farid R, Ranjbar S, Araghi ZJ, Pilz J, Schweighofer-Zwink G, Pirich C, et al. Molecular imaging in primary staging of prostate cancer patients: current aspects and future trends. Cancers (Basel). 2021;13:21. 10.3390/cancers13215360 PMC8582501 34771523

[6] Wang L, Wang L, Wang X, Wu D. The evolving role of PSMA-PET/CT in prostate cancer management: an umbrella review of diagnostic Restaging, therapeutic Redirection, and survival impact. Curr Oncol Rep. 2025;27(6):774–87. 40366535 10.1007/s11912-025-01682-2 PMC12227496

[7] Teo MY, Rathkopf DE, Kantoff P. Treatment of advanced prostate cancer. Annu Rev Med. 2019;70:479–99. 30691365 10.1146/annurev-med-051517-011947 PMC6441973

[8] Varaprasad GL, Gupta VK, Prasad K, Kim E, Tej MB, Mohanty P et al. Recent advances and future perspectives in the therapeutics of prostate cancer. Exp Hematol Oncol. 2023;12(80). 10.1186/s40164-023-00444-9 PMC10517568 37740236

[9] Fendler WP, Ferdinandus J, Czernin J, Eiber M, Flavell RR, Behr SC, et al. Impact of ^68^Ga-PSMA-11 PET on the management of recurrent prostate cancer in a prospective single-arm clinical trial. J Nucl Med. 2020;61(12):1793–9. 32358094 10.2967/jnumed.120.242180 PMC9364898

[10] Sartor O, de Bono J, Chi KN, Fizazi K, Herrmann K, Rahbar K, et al. Lutetium-177–PSMA-617 for metastatic Castration-Resistant prostate cancer. N Engl J Med. 2021;385(12):1091–103. 34161051 10.1056/NEJMoa2107322 PMC8446332

[11] Hofman MS, Emmett L, Sandhu S, Iravani A, Joshua AM, Goh JC, et al. [177Lu]Lu-PSMA-617 versus cabazitaxel in patients with metastatic castration-resistant prostate cancer (TheraP): a randomised, open-label, phase 2 trial. Lancet. 2021;397(10276):797–804. 33581798 10.1016/S0140-6736(21)00237-3

[12] Sathekge MM, Lawal IO, Bal C, Bruchertseifer F, Ballal S, Cardaci G, et al. Actinium-225-PSMA radioligand therapy of metastatic castration-resistant prostate cancer (WARMTH act): a multicentre, retrospective study. Lancet Oncol. 2024;25(2):175–83. 38218192 10.1016/S1470-2045(23)00638-1

[13] Boschi A, Martini P, Janevik-Ivanovska E, Duatti A. The emerging role of copper-64 radiopharmaceuticals as cancer theranostics. Drug Discov Today. 2018;23(8):1489–501. 29635027 10.1016/j.drudis.2018.04.002

[14] do Carmo SJC, Scott PJH, Alves F. Production of radiometals in liquid targets. EJNMMI Radiopharm Chem. 2020;5(1):2. 10.1186/s41181-019-0088-x 31925619 10.1186/s41181-019-0088-x PMC6954154

[15] Mou L, Martini P, Pupillo G, Cieszykowska I, Cutler CS, Mikołajczak R. 67Cu production capabilities: a mini review. Molecules. 2022;27(5):1501. 10.3390/molecules27051501 10.3390/molecules27051501 PMC8912090 35268600

[16] Fonseca AI, Do Carmo SJC, Hrynchak I, Alves V, Alves F, Abrunhosa AJ. Purification of copper radioisotopes for medical applications: chromatographic methods and challenges. Sep Purif Rev. 2024;53(3):289–310.

[17] Mccarthy DW, Bass LA, Cutler PD, Shefer RE, Klinkowstein RE, Herrero P, et al. High purity production and potential applications of Copper-60 and Copper-61. Nucl Med Biol. 1999;26(4):351–8. 10382836 10.1016/s0969-8051(98)00113-9

[18] Szelecsfinyi F, Blessing G, Qaiw SM. Excitation functions of proton induced nuclear reactions on enriched 61Ni and 64Ni: possibility of production of no-carrier-added 61Cu and 64Cu at a small cyclotron. Appl Radiat Isot. 1993;44(3):575–80.

[19] Asad AH, V. Smith S, Chan S, Jeffery CM, Morandeau L, Price RI. Cyclotron production of 61Cu using natural Zn & enriched 64Zn targets. AIP Conf Proc. 2012;1509:91–5.

[20] Do Carmo SJC, Alves VHP, Alves F, Abrunhosa AJ. Fast and cost-effective cyclotron production of 61Cu using a NatZn liquid target: an opportunity for radiopharmaceutical production and R&d. Dalton Trans. 2017;46(42):14556–60. 28702664 10.1039/c7dt01836c

[21] Dellepiane G, Casolaro P, Mateu I, Scampoli P, Voeten N, Braccini S. Cross-section measurement for an optimized ^61^Cu production at an 18 MeV medical cyclotron from natural Zn and enriched ^64^Zn solid targets. Appl Radiat Isot. 2022;190:110466. 10.1016/j.apradiso.2022.110466 36174333 10.1016/j.apradiso.2022.110466

[22] Fonseca AI, Alves VH, Do Carmo SJC, Silva M, Hrynchak I, Alves F, et al. Production of GMP-compliant clinical amounts of Copper-61 radiopharmaceuticals from liquid targets. Pharmaceuticals. 2022;15(6):723. 10.3390/ph15060723 35745642 10.3390/ph15060723 PMC9231368

[23] Svedjehed J, Kutyreff CJ, Engle JW, Gagnon K. Automated, cassette-based isolation and formulation of high-purity [^61^Cu]CuCl_2_ from solid Ni targets. EJNMMI Radiopharm Chem. 2020;5(1):21. 10.1186/s41181-020-00108-7 33151400 10.1186/s41181-020-00108-7 PMC7644601

[24] Muramatsu H, Shirai E, Nakahara H, Murakami Y. Alpha particle bombardment of natural nickel target for the production of 61Cu. Int J Appl Radiat Isot. 1978;29:611–4.

[25] Fukumura T, Okada K, Szelecsényi F, Kovács Z, Suzuki K. Practical production of 61Cu using natural Co target and its simple purification with a chelating resin for 61Cu-ATSM. Radiochim Acta. 2004;92:209–14.

[26] Avila-Rodriguez MA, Nye JA, Nickles RJ. Simultaneous production of high specific activity ^64^Cu and ^61^Co with 11.4 MeV protons on enriched ^64^Ni nuclei. Appl Radiat Isot. 2007;65(10):1115–20. 17669663 10.1016/j.apradiso.2007.05.012

[27] Alves F, Alves VHP, Do Carmo SJC, Neves ACB, Silva M, Abrunhosa AJ. Production of copper-64 and gallium-68 with a medical cyclotron using liquid targets. Mod Phys Lett A. 2017;32(17):1740013. 10.1142/S0217732317400132

[28] Szelecsényi F, Steyn GF, Kovács Z, Vermeulen C, Van Der Meulen NP, Dolley SG, et al. Investigation of the 66Zn(p,2pn)64Cu and 68Zn(p,x)64Cu nuclear processes up to 100 MeV: production of 64Cu. Nucl Instrum Methods Phys Res B. 2005;240(3):625–37.

[29] Szelecsényi F, Kovács Z, Nagatsu K, Zhang MR, Suzuki K. Excitation function of (p,α) nuclear reaction on enriched 67Zn: possibility of production of 64Cu at low energy cyclotron. Radiochim Acta. 2014;102(6):465–72.

[30] Bonardi ML, Groppi F, Birattari C, Gini L, Mainardi C, Ghioni A, et al. Thin-target excitation functions and optimization of simultaneous production of NCA copper-64 and gallium-66,67 by deuteron induced nuclear reactions on a natural zinc target. J Radioanal Nucl Chem. 2003;257(1):229–41.

[31] Kozempel J, Abbas K, Simonelli F, Zampese M, Holzwarth U, Gibson N, et al. A novel method for n.c.a. ^64^Cu production by the ^64^Zn(d,2p)^64^Cu reaction and dual ion-exchange column chromatography. Radiochim Acta. 2007;95(2):75–80.

[32] Abbasi IA, Zaidi JH, Arif M, Subhani MS. Measurement of fission neutron spectrum averaged cross sections of some threshold reactions on zinc: small-scale production of no-carrier-added 64Cu in a nuclear reactor. Radiochim Acta. 2006;94(2):63–7.

[33] Bokhari TH, Mushtaq A, Khan IU. Production of low and high specific activity 64Cu in a reactor. J Radioanal Nucl Chem. 2010;284(2):265–71.

[34] Johnsen AM, Heidrich BJ, Durrant CB, Bascom AJ, Ünlü K. Reactor production of 64Cu and 67Cu using enriched zinc target material. J Radioanal Nucl Chem. 2015;305(1):61–71.

[35] Chakravarty R, Chakraborty S, Vimalnath KV, Shetty P, Sarma HD, Hassan PA, et al. 64CuCl2 produced by direct neutron activation route as a cost-effective probe for cancer imaging: the journey has begun. RSC Adv. 2015;5(111):91723–33.

[36] RadioMedix, Curium Announce FDA. Approval of Detectnet (copper Cu 64 dotatate injection) in the U.S. [Internet]. 2020 [cited 2025 Jun 22]. Available from: https://www.curiumpharma.com/2020/09/08/radiomedix-and-curium-announce-fda-approval-of-detectnet-copper-cu-64-dotatate-injection-in-the-u-s/

[37] Katabuchi T, Watanabe S, Ishioka NS, Iida Y, Hanaoka H, Endo K, et al. Production of 67Cu via the 68Zn(p,2p)67Cu reaction and recovery of 68Zn target. J Radioanal Nucl Chem. 2008;277(2):467–70.

[38] Szelecsényi F, Steyn GF, Dolley SG, Kovács Z, Vermeulen C, van der Walt TN. Investigation of the ^68^Zn(p,2p)^67^Cu nuclear reaction: new measurements up to 40 MeV and compilation up to 100 MeV. Nucl Instrum Methods Phys Res B. 2009;267(11):1877–81.

[39] Medvedev DG, Mausner LF, Meinken GE, Kurczak SO, Schnakenberg H, Dodge CJ, et al. Development of a large scale production of 67Cu from 68Zn at the high energy proton accelerator: closing the 68Zn cycle. Appl Radiat Isot. 2012;70(3):423–9. 22142633 10.1016/j.apradiso.2011.10.007

[40] Kastleiner S, Coenen HH, Qaim SM. Possibility of production of 67Cu at a Small-Sized cyclotron via the (p,a)-Reaction on enriched 70Zn. Radiochim Acta. 1999;84(2):107–10.

[41] Kozempel J, Abbas K, Simonelli F, Bulgheroni A, Holzwarth U, Gibson N. Preparation of 67Cu via deuteron irradiation of 70Zn. Radiochim Acta. 2012;100(7):419–24.

[42] Shikata E. Research of radioisotope production with fast neutrons, (VI). J Nucl Sci Technol. 1964;1(5):177–80.

[43] O’brien HA. The preparation of 67Cu from 67Zn in a nuclear reactor. Int J Appl Radiat Isot. 1969;20:121–4.

[44] Mirzadeh S, Mausner LF, Srivastava SC. Production of no-carrier added 67Cu. Int J Rad Appl Instrum A. 1986;37(1):29–36. 3019924 10.1016/0883-2889(86)90192-9

[45] Spahn I, Coenen HH, Qaim SM. Enhanced production possibility of the therapeutic radionuclides 64Cu, 67Cu and 89Sr via (n,p) reactions induced by fast spectral neutrons. Radiochim Acta. 2004;92:183–6.

[46] Kin T, Nagai Y, Iwamoto N, Minato F, Iwamoto O, Hatsukawa Y, et al. New production routes for medical isotopes 64Cu and 67Cu using accelerator neutrons. J Phys Soc Jpn. 2013;82(3):034201. 10.7566/JPSJ.82.034201

[47] Sato N, Tsukada K, Watanabe S, Ishioka NS, Kawabata M, Saeki H, et al. First measurement of the radionuclide purity of the therapeutic isotope ^67^Cu produced by ^68^Zn(n,x) reaction using natC(d,n) neutrons. J Phys Soc Jpn. 2014;83(7):073201. 10.7566/JPSJ.83.073201

[48] Kawabata M, Hashimoto K, Saeki H, Sato N, Motoishi S, Takakura K, et al. Production and separation of 64Cu and 67Cu using 14 MeV neutrons. J Radioanal Nucl Chem. 2015;303(2):1205–9.

[49] Gopalakrishna A, Suryanarayana SV, Naik H, DIxit TS, Nayak BK, Kumar A, et al. Production, separation and supply prospects of 67Cu with the development of fast neutron sources and photonuclear technology. Radiochim Acta. 2018;106(7):549–57.

[50] Kawabata M, Motoishi S, Ohta A, Motomura A, Saeki H, Tsukada K, et al. Large scale production of 64Cu and 67Cu via the 64Zn(n,p)64Cu and 68Zn(n,np/d)67Cu reactions using accelerator neutrons. J Radioanal Nucl Chem. 2021;330(3):913–22.

[51] Marceau N, Kruck TPA, Mcconnell DB, Aspin N. The production of copper 67 from natural zinc using a linear accelerator. Int J Appl Radiat Isot. 1970;21:667–9. 5505004 10.1016/0020-708x(70)90121-3

[52] Stoner J, Gardner T, Gardner T. A comparison of DOTA and DiamSar chelates of high specific activity eLINAC produced ^67^Cu. J Nucl Med. 2016;57(supplement 2):1107.

[53] Hovhannisyan GH, Bakhshiyan TM, Dallakyan RK. Photonuclear production of the medical isotope 67Cu. Nucl Instrum Methods Phys Res B. 2021;498:48–51.

[54] Merrick MJ, Rotsch DA, Tiwari A, Nolen J, Brossard T, Song J, et al. Imaging and dosimetric characteristics of 67Cu. Phys Med Biol. 2021;66(3):035002. 10.1088/1361-6560/abca52 33496267 10.1088/1361-6560/abca52

[55] Conry RR, Copper. Inorganic & coordination chemistry. Encyclopedia of inorganic chemistry. John Wiley & Sons, Ltd.; 2006.

[56] Wadas TJ, Wong EH, Weisman GR, Anderson CJ. Coordinating radiometals of copper, gallium, indium, yttrium, and zirconium for PET and SPECT imaging of disease. Chem Rev. 2010;110(5):2858–902. 20415480 10.1021/cr900325h PMC2874951

[57] Wadas TJ, Wong EH, Weisman GR, Anderson CJ. Copper chelation chemistry and its role in copper radiopharmaceuticals. Curr Pharm Des. 2007;13(1):3–16. 17266585 10.2174/138161207779313768

[58] Zeglis BM, Lewis JS. A practical guide to the construction of radiometallated bioconjugates for positron emission tomography. Dalton Transactions [Internet]. 2011 May 31 [cited 2025 Aug 17];40(23):6168–95. Available from: https://pubs.rsc.org/en/content/articlehtml/2011/dt/c0dt01595d 10.1039/c0dt01595d PMC3773488 21442098

[59] Wu N, Kang CS, Sin I, Ren S, Liu D, Ruthengael VC, et al. Promising bifunctional chelators for copper 64-PET imaging: practical 64Cu radiolabeling and high in vitro and in vivo complex stability. J Biol Inorg Chem. 2016;21(2):177–84. 26666778 10.1007/s00775-015-1318-7 PMC5116241

[60] Fujibayashi Y, Taniuchi H, Yonekura Y, Ohtani H, Konishi J, Yokoyama A. Copper-62-ATSM: a new hypoxia imaging agent with high membrane permeability and low redox potential. J Nucl Med. 1997;38(7):1155–60. 9225812

[61] Wallhaus TR, Lacy J, Whang J, Green MA, Nickles RJ, Stone CK. Human biodistribution and dosimetry of the PET perfusion agent Copper-62-PTSM. J Nucl Med. 1998;39(11):1958–64. 9829589

[62] Follacchio GA, De Feo MS, De Vincentis G, Monteleone F, Liberatore M. Radiopharmaceuticals labelled with copper radionuclides: clinical results in human beings. Curr Radiopharm. 2017;11(1):22–33. 10.2174/1874471011666171211161851 29231149

[63] Xie F, Wei W. [64Cu]Cu-ATSM: an emerging theranostic agent for cancer and neuroinflammation. Eur J Nucl Med Mol Imaging. 2022;49(12):3964–72. 35918492 10.1007/s00259-022-05887-6

[64] Brown OC, Baguña Torres J, Holt KB, Blower PJ, Went MJ. Copper complexes with dissymmetrically substituted bis(thiosemicarbazone) ligands as a basis for PET radiopharmaceuticals: control of redox potential and lipophilicity. Dalton Trans. 2017;46(42):14612–30. 28703233 10.1039/c7dt02008b

[65] Boros E, Holland JP. Chemical aspects of metal ion chelation in the synthesis and application antibody-based radiotracers. J Labelled Comp Radiopharm. 2018;61(9):652–71. 29230857 10.1002/jlcr.3590 PMC5997514

[66] De Silva RA, Jain S, Lears KA, Chong HS, Kang CS, Sun X, et al. Copper-64 radiolabeling and biological evaluation of bifunctional chelators for radiopharmaceutical development. Nucl Med Biol. 2012;39(8):1099–104. 22743158 10.1016/j.nucmedbio.2012.05.009 PMC3470735

[67] Kubíček V, Böhmová Z, Ševčíková R, Vaněk J, Lubal P, Poláková Z, et al. NOTA complexes with Copper(II) and divalent metal ions: kinetic and thermodynamic studies. Inorg Chem. 2018;57(6):3061–72. 29488748 10.1021/acs.inorgchem.7b02929

[68] Kręcisz P, Stefańska K, Studziński J, Pitucha M, Czylkowska A, Szymański P. Radiocopper in radiopharmacy and medical use: current status and perspective. J Med Chem. 2025;68(3):2356–76. 39895089 10.1021/acs.jmedchem.4c02885 PMC11831595

[69] Boros E, Packard AB. Radioactive transition metals for imaging and therapy. Chem Rev. 2019;119(2):870–901. 30299088 10.1021/acs.chemrev.8b00281

[70] Tosato M, Franchi S, Isse AA, Del Vecchio A, Zanoni G, Alker A, et al. Is smaller better? Cu2+/Cu + coordination chemistry and Copper-64 radiochemical investigation of a 1,4,7-triazacyclononane-based sulfur-rich chelator. Inorg Chem. 2023;62(50):20621–33. 37115633 10.1021/acs.inorgchem.3c00621 PMC10731632

[71] Price EW, Orvig C. Matching chelators to radiometals for radiopharmaceuticals. Chem Soc Rev. 2014;43(1):260–90. 24173525 10.1039/c3cs60304k

[72] Smith SV. Molecular imaging with copper-64. J Inorg Biochem. 2004;98(11):1874–901. 15522415 10.1016/j.jinorgbio.2004.06.009

[73] Maheshwari V, Dearling JLJ, Treves ST, Packard AB. Measurement of the rate of copper(II) exchange for ^64^Cu complexes of bifunctional chelators. Inorg Chim Acta. 2012;393:318–23.

[74] Szymański P, Fraczek T, Markowicz M, Mikiciuk-Olasik E. Development of copper based drugs, radiopharmaceuticals and medical materials. Biometals. 2012;25(6):1089–112. 22914969 10.1007/s10534-012-9578-y PMC3496555

[75] Boswell CA, Sun X, Niu W, Weisman GR, Wong EH, Rheingold AL, et al. Comparative in vivo stability of Copper-64-labeled cross-bridged and conventional tetraazamacrocyclic complexes. J Med Chem. 2004;47(6):1465–74. 14998334 10.1021/jm030383m

[76] Silversides JD, Burke BP, Archibald SJ. Rapid synthesis of cross-bridged cyclam chelators for copper(II) complex formation. CR Chim. 2013;16(6):524–30.

[77] Jiang M, Ferdani R, Shokeen M, Anderson CJ. Comparison of two cross-bridged macrocyclic chelators for the evaluation of ^64^Cu-labeled-LLP2A, a peptidomimetic ligand targeting VLA-4-positive tumors. Nucl Med Biol. 2013;40(2):245–51. 23265977 10.1016/j.nucmedbio.2012.10.010 PMC3563241

[78] Zeng D, Ouyang Q, Cai Z, Xie XQ, Anderson CJ. New cross-bridged cyclam derivative CB-TE1K1P, an improved bifunctional chelator for copper radionuclides. Chem Commun. 2014;50(1):43–5. 10.1039/c3cc45928d PMC4277816 24141371

[79] Nedrow JR, Latoche JD, Day KE, Modi J, Ganguly T, Zeng D, et al. Targeting PSMA with a Cu-64 labeled phosphoramidate inhibitor for PET/CT imaging of variant PSMA-expressing xenografts in mouse models of prostate cancer. Mol Imaging Biol. 2016;18(3):402–10. 26552656 10.1007/s11307-015-0908-7

[80] Hyväkkä A, Virtanen V, Kemppainen J, Grönroos TJ, Minn H, Sundvall M. More than meets the eye: scientific rationale behind molecular imaging and therapeutic targeting of prostate-specific membrane antigen (PSMA) in metastatic prostate cancer and beyond. Cancers (Basel). 2021;13(9):2244. 34067046 10.3390/cancers13092244 PMC8125679

[81] Calais J, Czernin J. PSMA expression assessed by PET imaging is a required biomarker for selecting patients for any PSMA-targeted therapy. J Nucl Med. 2021;62(11):1489–91. 34725231 10.2967/jnumed.121.263159 PMC8612346

[82] Kuppermann D, Calais J, Marks LS. Imaging prostate cancer: clinical utility of prostate-specific membrane antigen. J Urol. 2022;207(4):769–78. 35085002 10.1097/JU.0000000000002457

[83] Nanus DM, Milowsky MI, Kostakoglu L, Smith-Jones PM, Vallabahajosula S, Goldsmith SJ et al. Clinical use of monoclonal antibody HuJ591 therapy: targeting prostate specific membrane antigen. J Urol. 2003;170(6 II). 10.1097/01.ju.0000095151.97404.7c 14610416

[84] Elsässer-Beile U, Wolf P, Gierschner D, Bühler P, Schultze-Seemann WG, Wetterauer U. A new generation of monoclonal and recombinant antibodies against cell-adherent prostate specific membrane antigen for diagnostic and therapeutic targeting of prostate cancer. Prostate. 2006;66(13):1359–70. 16894535 10.1002/pros.20367

[85] Elsässer-Beile U, Reischl G, Wiehr S, Bühler P, Wolf P, Alt K, et al. Pet imaging of prostate cancer xenografts with a highly specific antibody against the prostate-specific membrane antigen. J Nucl Med. 2009;50(4):606–11. 19289418 10.2967/jnumed.108.058487

[86] Alt K, Wiehr S, Ehrlichmann W, Reischl G, Wolf P, Pichler BJ, et al. High-resolution animal PET imaging of prostate cancer xenografts with three different ^64^Cu-labeled antibodies against native cell-adherent PSMA. Prostate. 2010;70(13):1413–21. 20687214 10.1002/pros.21176

[87] D’Souza JW, Hensley H, Doss M, Beigarten C, Torgov M, Olafsen T, et al. Cerenkov luminescence imaging as a modality to evaluate antibody-based PET radiotracers. J Nucl Med. 2017;58(1):175–80. 27539844 10.2967/jnumed.116.178780 PMC5209641

[88] Maier FC, Wild AM, Kirchen N, Holm F, Fuchs K, Schwenck J, et al. Comparative immuno-Cerenkov luminescence and -PET imaging enables detection of PSMA + tumors in mice using 64Cu-radiolabeled monoclonal antibodies. Appl Radiat Isot. 2019;143:149–55. 30445280 10.1016/j.apradiso.2018.09.006

[89] Wong P, Li L, Chea J, Delgado MK, Crow D, Poku E, et al. PET imaging of 64Cu-DOTA-scFv-anti-PSMA lipid nanoparticles (LNPs): enhanced tumor targeting over anti-PSMA ScFv or untargeted LNPs. Nucl Med Biol. 2017;47:62–8. 28126683 10.1016/j.nucmedbio.2017.01.004 PMC5348925

[90] Wong P, Li L, Chea J, Delgado MK, Poku E, Szpikowska B, et al. Synthesis, positron emission tomography imaging, and therapy of Diabody targeted drug lipid nanoparticles in a prostate cancer murine model. Cancer Biother Radiopharm. 2017;32(7):247–57. 28910151 10.1089/cbr.2017.2253 PMC5646751

[91] Fletcher NL, Houston ZH, Chandler PG, Yan E, Holgate R, Wheatcroft M, et al. Development of a novel engineered antibody format for PSMA-targeted radionuclide therapy. Mol Pharm. 2025;22(7):3666–3678. 10.1021/acs.molpharmaceut.4c01193 40403225 10.1021/acs.molpharmaceut.4c01193 PMC12670495

[92] Lesniak WG, Boinapally S, Banerjee SR, Behnam Azad B, Foss CA, Shen C, et al. Evaluation of PSMA-targeted PAMAM dendrimer nanoparticles in a murine model of prostate cancer. Mol Pharm. 2019;16(6):2590–604. 31002252 10.1021/acs.molpharmaceut.9b00181

[93] Lesniak WG, Boinapally S, Lofland G, Jiang Z, Foss CA, Azad BB, et al. Multimodal, PSMA-Targeted, PAMAM Dendrimer-Drug conjugates for treatment of prostate cancer: preclinical evaluation. Int J Nanomed. 2024;19:4995–5010. 10.2147/IJN.S454128 PMC11146619 38832336

[94] Grubmüller B, Baum RP, Capasso E, Singh A, Ahmadi Y, Knoll P, et al. 64Cu-PSMA-617 PET/CT imaging of prostate adenocarcinoma: first in-human studies. Cancer Biother Radiopharm. 2016;31(8):277–86. 27715146 10.1089/cbr.2015.1964

[95] Cantiello F, Gangemi V, Cascini GL, Calabria F, Moschini M, Ferro M, et al. Diagnostic accuracy of 64Copper prostate-specific membrane antigen positron emission tomography/computed tomography for primary lymph node staging of intermediate- to high-risk prostate cancer: our preliminary experience. Urology. 2017;106:139–45. 28438628 10.1016/j.urology.2017.04.019

[96] Cantiello F, Crocerossa F, Russo GI, Gangemi V, Ferro M, Vartolomei MD, et al. Comparison between 64Cu-PSMA-617 PET/CT and 18F-choline PET/CT imaging in early diagnosis of prostate cancer biochemical recurrence. Clin Genitourin Cancer. 2018;16(5):385–91. 29937067 10.1016/j.clgc.2018.05.014

[97] Annovazzi A, Faiella A, Pescarmona E, Sanguineti G, Sciuto R. Asymptomatic metastasis to thyroid cartilage detected by 18F-choline and 64Cu-PSMA PET/CT as a single site of disease relapse in a patient with castration-resistant prostate carcinoma. Clin Nucl Med. 2020;45(3):214–6. 31652161 10.1097/RLU.0000000000002786

[98] Farhan C, Mirzaei S. Soft tissue metastasis of the penis detected by copper-64 labeled prostate-specific membrane antigen positron emission tomography (64Cu–PSMA PET/CT) in a patient with prostate cancer. Asia Ocean J Nucl Med Biol. 2021;9(2):180–2. 34250148 10.22038/AOJNMB.2021.53922.1371 PMC8255521

[99] Calabria F, Pichler R, Leporace M, Wolfsgruber J, Coscarelli P, Dunzinger A, et al. 68Ga/64Cu PSMA Bio-Distribution in prostate cancer patients: potential pitfalls for different tracers. Curr Radiopharm. 2019;12(3):238–46. 31113354 10.2174/1874471012666190515090755

[100] Hoberück S, Wunderlich G, Michler E, Hölscher T, Walther M, Seppelt D, et al. Dual-time-point 64Cu-PSMA-617-PET/CT in patients suffering from prostate cancer. J Labelled Comp Radiopharm. 2019;62(8):523–32. 31042811 10.1002/jlcr.3745

[101] Cardoza-Ochoa DR, Rivera-Bravo B. A comparison of 18F-PSMA-1007 and 64Cu-PSMA in 2 patients with metastatic prostate cancer. Clin Nucl Med. 2022;47(2):E120–2. 34115708 10.1097/RLU.0000000000003758

[102] Lepage ML, Kuo HT, Roxin Á, Huh S, Zhang Z, Kandasamy R, et al. Toward 18F-labeled theranostics: a single agent that can be labeled with 18F, 64Cu, or 177Lu. Chembiochem. 2020;21(7):943–7. 31621172 10.1002/cbic.201900632

[103] Mirzaei S, Lipp R, Zandieh S, Leisser A. Single-center comparison of [64Cu]-DOTAGA-PSMA and [18F]-PSMA PET–CT for imaging prostate cancer. Curr Oncol. 2021;28(5):4167–73. 34677271 10.3390/curroncol28050353 PMC8534892

[104] Lee CH, Lim I, Woo SK, Kim K, Il, Lee KC, Song K, et al. The feasibility of 64Cu-PSMA I&T PET for prostate cancer. Cancer Biother Radiopharm. 2022;37(6):417–23. 33434438 10.1089/cbr.2020.4189

[105] Nelson BJB, Leier S, Wilson J, Wuest M, Doupe J, Andersson JD et al. 64Cu production via the 68Zn(p,nα)64Cu nuclear reaction: an untapped, cost-effective and high energy production route. Nucl Med Biol. 2024;128–9. 10.1016/j.nucmedbio.2024.108875 38199184

[106] Bernabeu TB, Mansi R, Del Pozzo L, Zanger S, Gaonkar RH, McDougall L, et al. 61Cu-PSMA–targeted PET for prostate cancer: from radiotracer development to first-in-human imaging. J Nucl Med. 2024;65(9):1427–34. 39025646 10.2967/jnumed.123.267126 PMC11372264

[107] Rodrigues D, Fonseca AI, do Carmo S, Sereno J, Hrynchak I, Moreira JN, et al. Is copper-61 the new Gallium-68? Automation and preclinical proof-of-concept of 61Cu-based radiopharmaceuticals for prostate cancer imaging. Pharmaceuticals. 2025;18(4):469. 40283906 10.3390/ph18040469 PMC12030277

[108] Bunda S, Kálmán-Szabó I, Szikra D, Fekete A, Szücs D, Szabó JP, et al. In vivo evaluation of Copper-61-labeled Prostate-specific membrane antigen targeting novel radiopharmaceutical: first steps toward clinical implementation. ACS Pharmacol Transl Sci. 2025;8:1580–90. 40534678 10.1021/acsptsci.4c00685 PMC12171877

[109] Zhang J, Niu S, Liu Y, Zhang X, Luan X, Liu H, et al. Real-time diagnosis of sampled lesions in targeted biopsy of prostate cancer using a novel tracer [64Cu]Cu-DOTA-PSMA-3Q: a pilot preclinical study. Eur J Nucl Med Mol Imaging. 2024;52(4):1257–1270. 10.1007/s00259-024-07000-5 10.1007/s00259-024-07000-5 PMC11839696 39614910

[110] Liu H, Zhang X, Zhang J, Pan Y, Wen H, Xu X, et al. Comparison of 64Cu-DOTA-PSMA-3Q and 64Cu-NOTA-PSMA-3Q utilizing NOTA and DOTA as bifunctional chelators in prostate cancer: preclinical assessment and preliminary clinical PET/CT imaging. Eur J Nucl Med Mol Imaging. 2025;52(8):2792–2803. 10.1007/s00259-025-07131-3 10.1007/s00259-025-07131-3 PMC12162759 39954062

[111] Chen F, Zhang H, Zhan Y, Huang X, He Z, Ma D, et al. Preclinical and clinical evaluation of [64Cu]Cu-PSMA-Q PET/CT for prostate cancer detection and its comparison with [18F]FDG imaging. Sci Rep. 2025;15(1):14431. 10.1038/s41598-025-98757-8 10.1038/s41598-025-98757-8 PMC12032344 40281230

[112] Bartholomä M, Rosar F, Burgard C, Maus S, Schreck MV, Ezziddin S. Novel 64Cu-labeled trimeric PSMA ligand unequivocally identifies local recurrence of prostate cancer ambiguously imaged by 18F-DCFPyL PSMA PET/CT. Clin Nucl Med. 2025. 10.1097/RLU.0000000000006010 40472262

[113] Lim J, Guan B, Nham K, Hao G, Sun X, Simanek EE. Tumor uptake of triazine dendrimers decorated with four, sixteen, and sixty-four PSMA-targeted ligands: passive versus active tumor targeting. Biomolecules. 2019;9(9):421. 10.3390/biom9090421 10.3390/biom9090421 PMC6770530 31466360

[114] Banerjee SR, Pullambhatla M, Foss CA, Nimmagadda S, Ferdani R, Anderson CJ, et al. ^64^Cu-labeled inhibitors of prostate-specific membrane antigen for PET imaging of prostate cancer. J Med Chem. 2014;57(6):2657–69. 24533799 10.1021/jm401921j PMC3983358

[115] Gourni E, Canovas C, Goncalves V, Denat F, Meyer PT, Maecke HR. (R)-NODAGA-PSMA: a versatile precursor for radiometal labeling and nuclear imaging of PSMA-positive tumors. PLoS ONE. 2015;10(12). 10.1371/journal.pone.0145755 10.1371/journal.pone.0145755 PMC4689406 26700033

[116] Läppchen T, Kiefer Y, Holland JP, Bartholomä MD. In vitro and in vivo evaluation of the bifunctional chelator NODIA-Me in combination with a prostate-specific membrane antigen targeting vector. Nucl Med Biol. 2018;60:45–54. 29571066 10.1016/j.nucmedbio.2018.03.002

[117] Umbricht CA, Benešová M, Hasler R, Schibli R, Van Der Meulen NP, Müller C. Design and preclinical evaluation of an albumin-binding PSMA ligand for 64Cu-based PET imaging. Mol Pharm. 2018;15(12):5556–64. 30376344 10.1021/acs.molpharmaceut.8b00712

[118] Lee I, Kim MH, Lee K, Oh K, Lim H, Ahn JH, et al. Comparison of the effects of DOTA and NOTA chelators on ^64^Cu-Cudotadipep and ^64^Cu-Cunotadipep for prostate cancer. Diagnostics. 2023;13:16. 10.3390/diagnostics13162649 PMC10453766 37627908

[119] Lewis MR, Schaedler AW, Ho K, Van, Golzy M, Mathur A, Pun M et al. Evaluation of a bimodal, matched pair theranostic agent targeting prostate-specific membrane antigen. Nucl Med Biol. 2024;136–7. 10.1016/j.nucmedbio.2024.108938 39032262

[120] Milot MC, Benesty OB, Dumulon-Perreault V, Ait-Mohand S, Richard PO, Rousseau É, et al. 64Cu-DOTHA2-PSMA, a novel PSMA PET radiotracer for prostate cancer with a long imaging time window. Pharmaceuticals. 2022;15(8):996. 10.3390/ph15080996 10.3390/ph15080996 PMC9412875 36015144

[121] Milot MC, Bélissant-Benesty O, Dumulon-Perreault V, Ait-Mohand S, Geha S, Richard PO, et al. Theranostic ^64^Cu-DOTHA2-PSMA allows low toxicity radioligand therapy in mice prostate cancer model. Front Oncol. 2023;13:1073491. 10.3389/fonc.2023.1073491 36741017 10.3389/fonc.2023.1073491 PMC9889868

[122] Hao G, Kumar A, Dobin T, Öz OK, Hsieh JT, Sun X. A multivalent approach of imaging probe design to overcome an endogenous anion binding competition for noninvasive assessment of prostate specific membrane antigen. Mol Pharm. 2013;10(8):2975–85. 23768233 10.1021/mp4000844 PMC3757929

[123] Harmatys KM, Overchuk M, Chen J, Ding L, Chen Y, Pomper MG, et al. Tuning pharmacokinetics to improve tumor accumulation of a Prostate-Specific membrane Antigen-Targeted phototheranostic agent. Bioconjug Chem. 2018;29(11):3746–56. 30350576 10.1021/acs.bioconjchem.8b00636 PMC6813806

[124] Sevcenco S, Klingler HC, Eredics K, Friedl A, Schneeweiss J, Knoll P, et al. Application of Cu-64 NODAGA-PSMA PET in prostate cancer. Adv Ther. 2018;35(6):779–84. 10.1007/s12325-018-0711-3 29777523 10.1007/s12325-018-0711-3

[125] dos Santos JC, Beijer B, Bauder-Wüst U, Schäfer M, Leotta K, Eder M, et al. Development of novel PSMA ligands for imaging and therapy with copper isotopes. J Nucl Med. 2020;61(1):70–9. 31541034 10.2967/jnumed.119.229054

[126] Liu T, Liu C, Zhang Z, Zhang N, Guo X, Xia L, et al. 64Cu-PSMA-BCH: a new radiotracer for delayed PET imaging of prostate cancer. Eur J Nucl Med Mol Imaging. 2021;48(13):4508–16. 34170361 10.1007/s00259-021-05426-9

[127] Zia NA, Cullinane C, Van Zuylekom JK, Waldeck K, McInnes LE, Buncic G, et al. A bivalent inhibitor of prostate specific membrane antigen radiolabeled with copper-64 with high tumor uptake and retention. Angew Chem. 2019;131(42):15133–6. 10.1002/anie.201908964 31437347

[128] McInnes LE, Cullinane C, Roselt PD, Jackson S, Blyth BJ, van Dam EM, et al. Therapeutic efficacy of a bivalent inhibitor of Prostate-Specific membrane antigen labeled with 67Cu. J Nucl Med. 2021;62(6):829–32. 33067341 10.2967/jnumed.120.251579 PMC8729863

[129] Kelly JM, Ponnala S, Amor-Coarasa A, Zia NA, Nikolopoulou A, Nikolopoulou A, et al. Preclinical evaluation of a high-affinity sarcophagine-containing PSMA ligand for 64Cu/67Cu-based theranostics in prostate cancer. Mol Pharm. 2020;17(6):1954–62. 32286841 10.1021/acs.molpharmaceut.0c00060

[130] PROPELLER Trial Results – SAR-bisPSMA, Safe. Well Tolerated and Efficacious in the Detection of Prostate Cancer [Internet]. 2023 [cited 2025 May 18]. Available from: https://www.claritypharmaceuticals.com/news/propeller_results/

[131] Registrational Phase III, CLARIFY trial in prostate cancer commences [Internet]. 2023 [cited 2025 May 18]. Available from: https://www.claritypharmaceuticals.com/news/clarifyphase3/

[132] Initial COBRA. results: Clarity’s SAR-bisPSMA is safe and highly effective in detecting tumours in prostate cancer patients. Phase 3 planning underway. 2024 Feb 15 [cited 2025 May 18]; Available from: https://www.claritypharmaceuticals.com/news/cobra_results/

[133] Positive guidance from the U.S. FDA on 64Cu-SAR-bisPSMA Phase III trial in patients with recurrence of prostate cancer [Internet]. 2024 [cited 2025 May 18]. Available from: https://www.claritypharmaceuticals.com/news/fda_amplify/

[134] SECuRE trial advances. No dose limiting toxicities and strong preliminary efficacy data in first multi-dose cohort [Internet]. 2024 [cited 2025 May 18]. Available from: https://www.claritypharmaceuticals.com/news/c4_update/

[135] SECuRE trial update. 92% of pre-chemo participants experience greater than 35% drop in PSA levels across all cohorts. Cohort Expansion Phase commences. [Internet]. 2025 [cited 2025 May 18]. Available from: https://www.claritypharmaceuticals.com/news/secure-update/

[136] Clarity Update. Complete response in first patient ever treated with 2 doses of Cu-67 SAR-bisPSMA at 8GBq [Internet]. 2024 [cited 2025 May 18]. Available from: https://www.claritypharmaceuticals.com/news/complete_response/

[137] SECuRE trial update. First patient treated in the Phase II Cohort Expansion [Internet]. 2025 [cited 2025 May 18]. Available from: https://www.claritypharmaceuticals.com/news/secure-fp-phase2/

[138] St Vincent’. s Hospital to conduct head-to-head trial with Clarity’s SAR-bisPSMA diagnostic product [Internet]. 2024 [cited 2025 May 18]. Available from: https://www.claritypharmaceuticals.com/news/co-psma/

[139] First 2 participants dosed with Cu. -64 SAR-bisPSMA in Co-PSMA trial [Internet]. 2024 [cited 2025 May 18]. Available from: https://www.claritypharmaceuticals.com/news/co-psma-fp/

[140] Li S, Nguyen A, Counter W, John NC, De Leon J, Hruby G, et al. Utility of ^64^Cu-sarcophagine-bombesin PET/CT in men with biochemically recurrent prostate cancer and negative or equivocal findings on ^68^Ga-PSMA-11 PET/CT. J Nucl Med. 2024;65(9):1371–5. 39089814 10.2967/jnumed.124.267881

[141] Rogers BE, Bigott HM, McCarthy DW, Manna D, Della, Kim J, Sharp TL, et al. MicroPET imaging of a Gastrin-Releasing peptide Receptor-Positive tumor in a mouse model of human prostate cancer using a 64Cu-Labeled Bombesin analogue. Bioconjug Chem. 2003;14(4):756–63. 12862428 10.1021/bc034018l

[142] Chen X, Park R, Hou Y, Tohme M, Shahinian AH, Bading JR, et al. MicroPET and autoradiographic imaging of GRP receptor expression with 64Cu-DOTA-[Lys3]Bombesin in human prostate adenocarcinoma xenografts. J Nucl Med. 2004;45(8):1390–7. 15299066

[143] Yang YS, Zhang X, Xiong Z, Chen X. Comparative in vitro and in vivo evaluation of two 64Cu-labeled Bombesin analogs in a mouse model of human prostate adenocarcinoma. Nucl Med Biol. 2006;33(3):371–80. 16631086 10.1016/j.nucmedbio.2005.12.011

[144] Biddlecombe GB, Rogers BE, De Visser M, Parry JJ, De Jong M, Erion JL, et al. Molecular imaging of gastrin-releasing peptide receptor-positive tumors in mice using 64Cu- and 86Y-DOTA-(Pro1,Tyr4)-bombesin(1–14). Bioconjug Chem. 2007;18(3):724–30. 17378600 10.1021/bc060281l

[145] Parry JJ, Kelly TS, Andrews R, Rogers BE. In vitro and in vivo evaluation of 64Cu-labeled DOTA-linker-bombesin(7–14) analogues containing different amino acid linker moieties. Bioconjug Chem. 2007;18(4):1110–7. 17503761 10.1021/bc0603788

[146] Garrison JC, Rold TL, Sieckman GL, Figueroa SD, Volkert WA, Jurisson SS, et al. In vivo evaluation and small-animal PET/CT of a prostate cancer mouse model using ^64^Cu bombesin analogs: side-by-side comparison of the CB-TE2A and DOTA chelation systems. J Nucl Med. 2007;48(8):1327–37. 17631556 10.2967/jnumed.107.039487

[147] Abiraj K, Mansi R, Tamma ML, Fani M, Forrer F, Nicolas G, et al. Bombesin antagonist-based radioligands for translational nuclear imaging of gastrin-releasing peptide receptor-positive tumors. J Nucl Med. 2011;52(12):1970–8. 22080443 10.2967/jnumed.111.094375

[148] Wieser G, Mansi R, Grosu AL, Schultze-Seemann W, Dumont-Walter RA, Meyer PT, et al. Positron emission tomography (PET) imaging of prostate cancer with a Gastrin releasing peptide receptor antagonist - from mice to men. Theranostics. 2014;4(4):412–9. 24578724 10.7150/thno.7324 PMC3936293

[149] Prasanphanich AF, Nanda PK, Rold TL, Ma L, Lewis MR, Garrison JC et al. [64Cu-NOTA-8-Aoc-BBN(7–14)NH2] targeting vector for positron-emission tomography imaging of gastrin-releasing peptide receptor-expressing tissues. Proc Natl Acad Sci U S A [Internet]. 2007;104(30):12462–7. Available from: www.pnas.orgcgidoi10.1073pnas.0705347104. 10.1073/pnas.0705347104 PMC1914305 17626788

[150] Lane SR, Nanda P, Rold TL, Sieckman GL, Figueroa SD, Hoffman TJ, et al. Optimization, biological evaluation and micropet imaging of copper-64-labeled Bombesin agonists, [64Cu-NO2A-(X)-BBN(7–14)NH2], in a prostate tumor xenografted mouse model. Nucl Med Biol. 2010;37(7):751–61. 20870150 10.1016/j.nucmedbio.2010.04.016

[151] Craft JM, De Silva RA, Lears KA, Andrews R, Liang K, Achilefu S, et al. In vitro and in vivo evaluation of a ^64^Cu-labeled NOTA-Bn-SCN-Aoc-bombesin analogue in gastrin-releasing peptide receptor expressing prostate cancer. Nucl Med Biol. 2012;39(5):609–16. 22261146 10.1016/j.nucmedbio.2011.12.004 PMC3341490

[152] Nanda PK, Pandey U, Bottenus BN, Rold TL, Sieckman GL, Szczodroski AF, et al. Bombesin analogues for gastrin-releasing peptide receptor imaging. Nucl Med Biol. 2012;39(4):461–71. 22261143 10.1016/j.nucmedbio.2011.10.009

[153] Nanda PK, Wienhoff BE, Rold TL, Sieckman GL, Szczodroski AF, Hoffman TJ, et al. Positron-emission tomography (PET) imaging agents for diagnosis of human prostate cancer: agonist vs. Antagonist ligands. Vivo (Brooklyn). 2012;26(4):583–92. 22773572

[154] Liu Y, Hu X, Liu H, Bu L, Ma X, Cheng K, et al. A comparative study of radiolabeled Bombesin analogs for the PET imaging of prostate cancer. J Nucl Med. 2013;54(12):2132–8. 24198391 10.2967/jnumed.113.121533 PMC4215198

[155] Baun C, Mitran B, Rinne SS, Dam JH, Olsen BB, Tolmachev V, et al. Preclinical evaluation of the copper-64 labeled GRPR-antagonist RM26 in comparison with the cobalt-55 labeled counterpart for PET-imaging of prostate cancer. Molecules. 2020;25(24):5993. 10.3390/molecules25245993 33352838 10.3390/molecules25245993 PMC7766840

[156] Gourni E, Mansi R, Jamous M, Waser B, Smerling C, Burian A, et al. N-terminal modifications improve the receptor affinity and pharmacokinetics of radiolabeled peptidic gastrin-releasing peptide receptor antagonists: examples of 68Ga- and 64Cu-labeled peptides for PET imaging. J Nucl Med. 2014;55(10):1719–25. 25146125 10.2967/jnumed.114.141242

[157] Makris G, Shegani A, Kankanamalage PHA, Kuchuk M, Bandari RP, Smith CJ, et al. Preclinical evaluation of novel 64Cu-labeled gastrin-releasing peptide receptor bioconjugates for PET imaging of prostate cancer. Bioconjug Chem. 2021;32(7):1290–7. 33434428 10.1021/acs.bioconjchem.0c00656

[158] Kim MH, Park JA, Woo SK, Lee KC, An G, Il, Kim BS, et al. Evaluation of a 64Cu-labeled 1,4,7-triazacyclononane, 1-glutaric acid-4,7 acetic acid (NODAGA)-galactose-bombesin analogue as a PET imaging probe in a gastrin-releasing peptide receptor-expressing prostate cancer xenograft model. Int J Oncol. 2015;46(3):1159–68. 25586565 10.3892/ijo.2015.2832

[159] Mansour N, Dumulon-Perreault V, Ait-Mohand S, Paquette M, Lecomte R, Guérin B. Impact of dianionic and dicationic linkers on tumor uptake and biodistribution of [64Cu]Cu/NOTA peptide-based gastrin-releasing peptide receptors antagonists. J Labelled Comp Radiopharm. 2017;60(4):200–12. 28129446 10.1002/jlcr.3491

[160] Lears KA, Ferdani R, Liang K, Zheleznyak A, Andrews R, Sherman CD, et al. In vitro and in vivo evaluation of 64Cu-labeled SarAr-bombesin analogs in gastrin-releasing peptide receptor-expressing prostate cancer. J Nucl Med. 2011;52(3):470–7. 21321264 10.2967/jnumed.110.082826 PMC3088991

[161] Gourni E, Del Pozzo L, Kheirallah E, Smerling C, Waser B, Reubi JC, et al. Copper-64 labeled macrobicyclic sarcophagine coupled to a GRP receptor antagonist shows great promise for PET imaging of prostate cancer. Mol Pharm. 2015;12(8):2781–90. 26132879 10.1021/mp500671j

[162] Recruitment target achieved for Phase II SAR-Bombesin prostate cancer trial [Internet]. 2023 [cited 2025 Jun 12]. Available from: https://www.claritypharmaceuticals.com/news/sabre_closed/

[163] Huynh TT, van Dam EM, Sreekumar S, Mpoy C, Blyth BJ, Muntz F, et al. Copper-67-labeled bombesin peptide for targeted radionuclide therapy of prostate cancer. Pharmaceuticals. 2022;15(6):728. 10.3390/ph15060728 35745647 10.3390/ph15060728 PMC9229378

[164] Clarity commences COMBAT theranostic prostate cancer trial in the US [Internet]. 2023 [cited 2025 Jun 12]. Available from: https://www.claritypharmaceuticals.com/news/combat_commences-us/

[165] Juran S, Walther M, Stephan H, Bergmann R, Steinbach J, Kraus W, et al. Hexadentate bispidine derivatives as versatile bifunctional chelate agents for copper(II) radioisotopes. Bioconjug Chem. 2009;20(2):347–59. 19173600 10.1021/bc800461e

[166] Bergmann R, Ruffani A, Graham B, Spiccia L, Steinbach J, Pietzsch J, et al. Synthesis and radiopharmacological evaluation of ^64^Cu-labeled Bombesin analogs featuring a bis(2-pyridylmethyl)-1,4,7-triazacyclononane chelator. Eur J Med Chem. 2013;70:434–46. 24184988 10.1016/j.ejmech.2013.10.013

[167] Mansour N, Paquette M, Ait-Mohand S, Dumulon-Perreault V, Guérin B. Evaluation of a novel GRPR antagonist for prostate cancer PET imaging: [64Cu]-DOTHA2-PEG-RM26. Nucl Med Biol. 2018;56:31–8. 29154145 10.1016/j.nucmedbio.2017.10.006

[168] Cai H, Xie F, Mulgaonkar A, Chen L, Sun X, Hsieh JT, et al. Bombesin functionalized ^64^Cu-copper sulfide nanoparticles for targeted imaging of orthotopic prostate cancer. Nanomedicine. 2018;13(14):1695–1705. 10.2217/nnm-2018-0062 29786467 10.2217/nnm-2018-0062

[169] Fournier P, Dumulon-Perreault V, Ait-Mohand S, Langlois R, Bénard F, Lecomte R et al. Comparative study of 64Cu/NOTA-[D-Tyr6,bAla11,Thi13,Nle14]BBN(6–14) monomer and dimers for prostate cancer PET imaging. EJNMMI Res [Internet]. 2012;2(8). Available from: http://www.ejnmmires.com/content/2/1/8 10.1186/2191-219X-2-8 PMC3323469 22333272

[170] Bandari RP, Jiang Z, Reynolds TS, Bernskoetter NE, Szczodroski AF, Bassuner KJ, et al. Synthesis and biological evaluation of copper-64 radiolabeled [DUPA-6-Ahx-(NODAGA)-5-Ava-BBN(7–14)NH2], a novel bivalent targeting vector having affinity for two distinct biomarkers (GRPr/PSMA) of prostate cancer. Nucl Med Biol. 2014;41(4):355–63. 24508213 10.1016/j.nucmedbio.2014.01.001 PMC4041584

[171] Matsumura K, Zouda M, Wada Y, Yamashita F, Hashida M, Watanabe Y, et al. Urokinase injection-triggered clearance enhancement of a 4-arm PEG-conjugated ^64^Cu-bombesin analog tetramer: a novel approach for the improvement of PET imaging contrast. Int J Pharm. 2018;545(1–2):206–14. 29746998 10.1016/j.ijpharm.2018.05.014

[172] Cai W, Wu Y, Chen K, Cao Q, Tice DA, Chen X. In vitro and in vivo characterization of 64Cu-labeled Abegrin™, a humanized monoclonal antibody against integrin αvβ3. Cancer Res. 2006;66(19):9673–81. 17018625 10.1158/0008-5472.CAN-06-1480

[173] Liu Z, Li ZB, Cao Q, Liu S, Wang F, Chen X. Small-animal PET of tumors with ^64^Cu-labeled RGD-bombesin heterodimer. J Nucl Med. 2009;50(7):1168–77. 19525469 10.2967/jnumed.108.061739

[174] Jackson AB, Nanda PK, Rold TL, Sieckman GL, Szczodroski AF, Hoffman TJ, et al. 64Cu-NO2A-RGD-Glu-6-Ahx-BBN(7–14)NH2: a heterodimeric targeting vector for positron emission tomography imaging of prostate cancer. Nucl Med Biol. 2012;39(3):377–87. 22226021 10.1016/j.nucmedbio.2011.10.004 PMC3629973

[175] Durkan K, Jiang Z, Rold TL, Sieckman GL, Hoffman TJ, Bandari RP, et al. A heterodimeric [RGD-Glu-[64Cu-NO2A]-6-Ahx-RM2] αvβ3/GRPr-targeting antagonist radiotracer for PET imaging of prostate tumors. Nucl Med Biol. 2014;41(2):133–9. 24480266 10.1016/j.nucmedbio.2013.11.006 PMC4022290

[176] Lucente E, Liu H, Liu Y, Hu X, Lacivita E, Leopoldo M, et al. Novel ^64^Cu labeled RGD2-BBN heterotrimers for PET imaging of prostate cancer. Bioconjug Chem. 2018;29(5):1595–604. 29587479 10.1021/acs.bioconjchem.8b00113

[177] Yuan J, Zhang H, Kaur H, Oupicky D, Peng F. Synthesis and characterization of theranostic poly(HPMA)-c(RGDyK)-DOTA-64Cu copolymer targeting tumor angiogenesis: tumor localization visualized by positron emission tomography. Mol Imaging. 2013;12(3):203–12. 10.2310/7290.2012.00038 23490439

[178] Jadvar H, Chen K, Park R, Yap LP, Vorobyova I, Swenson S, et al. Preclinical evaluation of a ^64^Cu-labeled disintegrin for PET imaging of prostate cancer. Amino Acids. 2019;51(10–12):1569–75. 31621030 10.1007/s00726-019-02794-3 PMC6881555

[179] Hu H, Li D, Liu S, Wang M, Moats R, Conti PS, et al. Integrin α2β1 targeted GdVO4:Eu ultrathin nanosheet for multimodal PET/MR imaging. Biomaterials. 2014;35(30):8649–58. 25043573 10.1016/j.biomaterials.2014.06.059

[180] Xiong C, Huang M, Zhang R, Song S, Lu W, Flores L, et al. In vivo small-animal PET/CT of EphB4 receptors using 64Cu-labeled peptide. J Nucl Med. 2011;52(2):241–8. 21233177 10.2967/jnumed.110.081943

[181] Ullman C, Mathonet P, Oleksy A, Diamandakis A, Tomei L, Demartis A, et al. High affinity binders to EphA2 isolated from abdurin scaffold libraries; characterization, binding and tumor targeting. PLoS ONE. 2015;10(8). 10.1371/journal.pone.0135278 10.1371/journal.pone.0135278 PMC4552014 26313909

[182] Pyo A, You SH, Sik Kim H, Young Kim J, Min JJ, Kim DY, et al. Production of ^64^Cu-labeled monobody for imaging of human EphA2-expressing tumors. Bioorg Med Chem Lett. 2020;30(14):127262. 10.1016/j.bmcl.2020.127262 32527560 10.1016/j.bmcl.2020.127262

[183] Zhang K, Aruva MR, Shanthly N, Cardi CA, Rattan S, Patel C, et al. PET imaging of VPAC1 expression in experimental and spontaneous prostate cancer. J Nucl Med. 2008;49(1):112–21. 18077536 10.2967/jnumed.107.043703 PMC5850935

[184] Tripathi S, Trabulsi EJ, Gomella L, Kim S, McCue P, Intenzo C, et al. VPAC1 targeted 64Cu-TP3805 positron emission tomography imaging of prostate cancer: preliminary evaluation in man. Urology. 2016;88:111–8. 26519886 10.1016/j.urology.2015.10.012 PMC4788593

[185] Deng H, Wang H, Zhang H, Wang M, Giglio B, Ma X, et al. Imaging neurotensin receptor in prostate cancer with ^64^Cu-labeled neurotensin analogs. Mol Imaging. 2017;16:1536012117711369. 10.1177/1536012117711369 28849698 10.1177/1536012117711369 PMC6081756

[186] Hao G, Hajibeigi A, León-Rodríguez LM, De, Öz OK, Sun X. Peptoid-based PET imaging of vascular endothelial growth factor receptor (VEGFR) expression. Am J Nucl Med Mol Imaging. 2011;1(1):65–75. 23133797 PMC3477717

[187] Pressly ED, Pierce RA, Connal LA, Hawker CJ, Liu Y. Nanoparticle PET/CT imaging of natriuretic peptide clearance receptor in prostate cancer. Bioconjug Chem. 2013;24(2):196–204. 23272904 10.1021/bc300473x PMC3578065

[188] Hall MA, Pinkston KL, Wilganowski N, Robinson H, Ghosh P, Azhdarinia A, et al. Comparison of mAbs targeting epithelial cell adhesion molecule for the detection of prostate cancer lymph node metastases with multimodal contrast agents: quantitative small-animal PET/CT and NIRF. J Nucl Med. 2012;53(9):1427–37. 22872743 10.2967/jnumed.112.106302

[189] Couture F, Levesque C, Dumulon-Perreault V, Ait-Mohand S, D’Anjou F, Day R, et al. PACE4-based molecular targeting of prostate cancer using an engineered 64Cu-radiolabeled peptide inhibitor. Neoplasia. 2014;16(8):634–43. 25220591 10.1016/j.neo.2014.07.010 PMC4235008

[190] Park JH, Zhang X, Ha H, Kim JY, Choi JY, Lee KH, et al. A high-affinity ^64^Cu-labeled ligand for PET imaging of hepsin: design, synthesis, and characterization. Pharmaceuticals. 2022;15(9):1109. 10.3390/ph15091109 36145330 10.3390/ph15091109 PMC9503212

[191] Han Z, Sergeeva O, Roelle S, Cheng H, Gao S, Li Y, et al. Preparation and evaluation of ZD2 peptide 64Cu-DOTA conjugate as a positron emission tomography probe for detection and characterization of prostate cancer. ACS Omega. 2019;4(1):1185–90. 30729224 10.1021/acsomega.8b02729 PMC6356864

[192] Konopka CJ, Woźniak M, Hedhli J, Siekierzycka A, Skokowski J, Pęksa R, et al. Quantitative imaging of the receptor for advanced glycation end-products in prostate cancer. Eur J Nucl Med Mol Imaging. 2020;47:2562–76. 10.1007/s00259-020-04721-1. 32166512 10.1007/s00259-020-04721-1

[193] Kumar A, Hao G, Liu L, Ramezani S, Hsieh JT, Öz OK, et al. Click-chemistry strategy for labeling antibodies with copper-64 via a cross-bridged tetraazamacrocyclic chelator scaffold. Bioconjug Chem. 2015;26(4):782–9. 25760776 10.1021/acs.bioconjchem.5b00102 PMC8374950

[194] David T, Hlinová V, Kubíček V, Bergmann R, Striese F, Berndt N, et al. Improved conjugation, 64-Cu radiolabeling, *in vivo* stability, and imaging using nonprotected bifunctional macrocyclic ligands: bis(Phosphinate) cyclam (BPC) chelators. J Med Chem. 2018;61(19):8774–96. 30180567 10.1021/acs.jmedchem.8b00932

[195] Arndt C, Bergmann R, Striese F, Merkel K, Máthé D, Loureiro LR, et al. Development and functional characterization of a versatile radio-/immunotheranostic tool for prostate cancer management. Cancers (Basel). 2022;14(8):1996. 10.3390/cancers14081996 10.3390/cancers14081996 PMC9027777 35454902

[196] Kubeil M, Neuber C, Starke M, Arndt C, Rodrigues Loureiro L, Hoffmann L, et al. 64Cu tumor labeling with hexadentate picolinic acid-based bispidine immunoconjugates. Chem Eur J. 2024;30(32). 10.1002/chem.202400366 38506263 10.1002/chem.202400366

[197] Hong H, Yan Y, Shi S, Graves SA, Krasteva LK, Nickles RJ, et al. PET of follicle-stimulating hormone receptor: broad applicability to cancer imaging. Mol Pharm. 2015;12(2):403–10. 25581441 10.1021/mp500766x PMC4351720

[198] Bergmann R, Chollet C, Els-Heindl S, Ullrich M, Berndt N, Pietzsch J, et al. Development of a Ghrelin receptor inverse agonist for positron emission tomography. Oncotarget [Internet]. 2021;12(5):450–74. Available from: www.oncotarget.com. 33747360 10.18632/oncotarget.27895 PMC7939532

[199] Persson M, Skovgaard D, Brandt-Larsen M, Christensen C, Madsen J, Nielsen CH, et al. First-in-human uPAR PET: imaging of cancer aggressiveness. Theranostics. 2015;5(12):1303–16. 26516369 10.7150/thno.12956 PMC4615734

[200] Curasight announces approval of clinical trial application (CTA). for phase 2 trial with uTRACE[^®^] in prostate cancer patients [Internet]. 2024 [cited 2025 Jun 12]. Available from: https://www.curasight.com/news/press-releases/2024/curasight-announces-approval-of-clinical-trial-application-cta-for-phase-2-trial-with-utrace-in-prostate-cancer-patients/

